# Dual Photochemistry of Benzimidazole

**DOI:** 10.1021/acs.joc.2c02560

**Published:** 2023-02-16

**Authors:** José P. L. Roque, Mário T. S. Rosado, Rui Fausto, Igor Reva

**Affiliations:** † CQC-IMS, Department of Chemistry, University of Coimbra, Coimbra 3004-535, Portugal; ‡ CIEPQPF, Department of Chemical Engineering, University of Coimbra, Coimbra 3030-790, Portugal

## Abstract

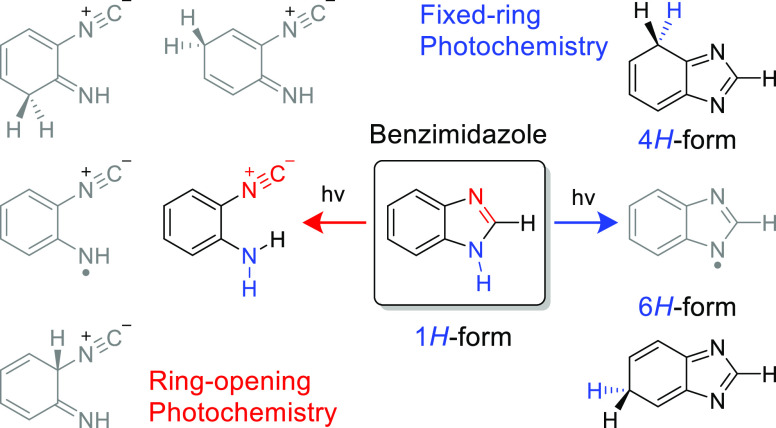

Monomers of benzimidazole trapped in an argon matrix at 15 K were characterized by vibrational spectroscopy and identified as 1*H*-tautomers exclusively. The photochemistry of matrix-isolated 1*H*-benzimidazole was induced by excitations with a frequency-tunable narrowband UV light and followed spectroscopically. Hitherto unobserved photoproducts were identified as 4*H*- and 6*H*-tautomers. Simultaneously, a family of photoproducts bearing the isocyano moiety was identified. Thereby, the photochemistry of benzimidazole was hypothesized to follow two reaction pathways: the fixed-ring and the ring-opening isomerizations. The former reaction channel results in the cleavage of the NH bond and formation of a benzimidazolyl radical and an H-atom. The latter reaction channel involves the cleavage of the five-membered ring and concomitant shift of the H-atom from the CH bond of the imidazole moiety to the neighboring NH group, leading to 2-isocyanoaniline and subsequently to the isocyanoanilinyl radical. The mechanistic analysis of the observed photochemistry suggests that detached H-atoms, in both cases, recombine with the benzimidazolyl or isocyanoanilinyl radicals, predominantly at the positions with the largest spin density (revealed using the natural bond analysis computations). The photochemistry of benzimidazole therefore occupies an intermediate position between the earlier studied prototype cases of indole and benzoxazole, which exhibit exclusively the fixed-ring and the ring-opening photochemistries, respectively.

## 1 Introduction

Benzimidazole (BzIm) derivatives are the structural bio-isosteres of naturally occurring purine bases, and the fact that benzimidazole produced biological effects has been recognized long ago.^1^ The research on benzimidazole chemistry goes back over a century.^2−4^ Benzimidazole has gained considerable attention in medicinal and pharmaceutical chemistry.^5^ BzIm derivatives interact with the biopolymers of the living system such as proteins, enzymes, and receptors and are frequently used in the development of new therapeutic agents against multiple diseases.^6−16^ The benzimidazole scaffold has also been reported as an organic corrosion inhibitor^17−22^ for different metals in different media and as a multifunctional unit in heteroaromatic molecular systems for optoelectronics, nonlinear optics, photovoltaics, optical sensing, and bioimaging.^23^ The structural and spectroscopic features of BzIm are well established. An overview of the most important reports devoted to the characterization of electronic and vibrational spectra of BzIm is provided in the Supporting Information.

**Scheme 1:**
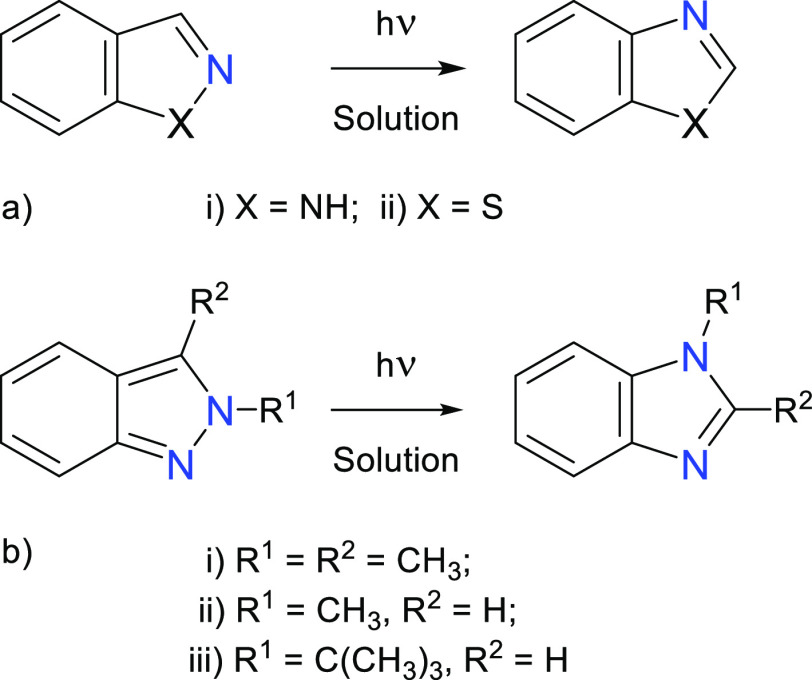
Photoreactivity of Benzoannulated Azoles in Solution^27−32^

Research on the photochemistry of BzIm derivatives has been addressed in solutions (Scheme 1). For instance, Cole *et al.* reported that photolysis of benzimidazole in various solvents with free access to air gives the unsymmetrical dehydrodimers.^24^ Similarly, Smitka *et al.* reported that in aqueous solution, the phototransformation of the free BzIm leads to dehydrodimerization,^25^ while inside cucurbit[8]uril, BzIm undergoes photohydrolysis to yield 2-amino-formanilide.^25^ The photochemistry of 2-iodo-substituted BzIm yields aromatic ring-fused benzimidazoles *via* substitutions of the benzimidazol-2-yl radical.^26^ The latter finding agrees with the report of Cole *et al.*,^24^ who found that 2-alkylbenzimidazoles do not give dimers but produce a mixture of acyl derivatives. N1-protonated benzopyrazoles^27,28^ and benzisothiazoles were found to undergo isomerizations to the corresponding benzimidazoles and benzothiazoles (Scheme 1a).^29^ Similar transformations occur with 2-alkyl-indazoles which undergo photoisomerization to 1-alkyl-benzimidazoles (Scheme 1b).^30−32^ Interestingly, photoisomerizations opposite to those shown in Scheme 1 have not been reported. Although some reports about the photochemistry of benzimidazole in condensed media have already been published,^24−26,33^ to the best of our knowledge, there are no reports on its photoinduced unimolecular transformations.

**Chart 1:**
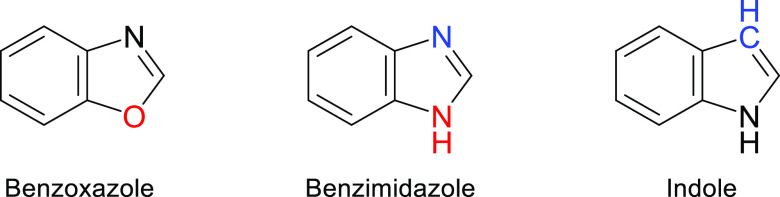
Molecular Structures of Benzoxazole, Benzimidazole, and Indole

Recently, we and others have successfully employed the matrix-isolation technique to study the photochemistry of monomeric benzoannulated azoles.^34^ In the context of this work, it is important to highlight the photochemistry of benzoxazole (BzOx) and indole (see Chart 1).

**Scheme 2:**
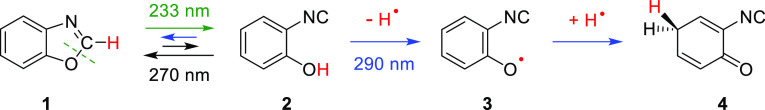
Photoinduced Reactivity of Monomeric Benzoxazole^35^^,^ Note color codes for the irradiations at different wavelengths.

Despite their structural similarity with BzIm, the photochemistry of monomeric BzOx and of monomeric indole was found to be rather different. For example, the photochemistry of BzOx **1** is initiated by the cleavage of the CO bond (λ = 233 nm), leading to quantitative formation of isomeric 2-iso-cyanophenol **2** (Scheme 2). Upon irradiation at longer wavelengths (λ = 270 nm), the open-ring 2-isocyanophenol photoisomerizes back to BzOx or undergoes photoinduced cleavage of the OH bond, leading to the formation of 2-isocyano-phenoxyl radical **3** and an H atom (also a radical). The subsequent recombination of the radical pair at different positions of **3** leads, among other products, to isocyano-substituted cyclohexadienone **4** (Scheme 2).

**Scheme 3:**
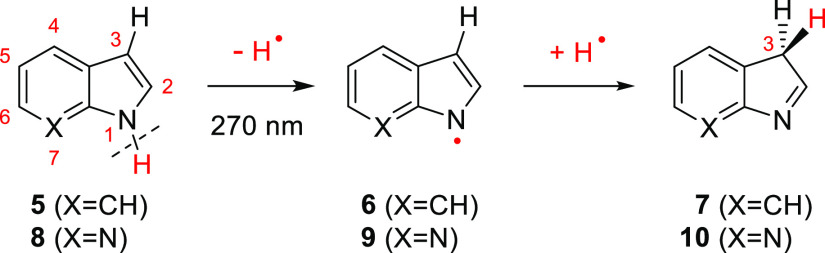
Photoinduced Reactivity of Monomeric Indole and 7-Azaindole^36,37^^,^ Numbering of heavy atoms of the starting compounds and ring position 3 of the photoproducts are shown in red.

In the case of indole (or its isomeric 7-azaindole), the photoinduced ring-opening reactions were not observed. Instead, the photochemical reactions involved the cleavage of the NH bond. The indolyl radical **6** or 7-azaindolyl radical **9** (along with the photodetached H-atom) were found to play a crucial role in the formation of prototropic 3*H*-tautomers of indole (**7**)^36^ or azaindole (**10**)^37^ (Scheme 3).

One can expect that the photochemistry of monomeric BzIm might bear similarities with that of either BzOx or indole, or both, making it unpredictable and challenging. Herein, we report on the photochemistry of monomeric benzimidazole isolated in a low-temperature Ar matrix, by employing infrared (IR) spectroscopy to characterize its photoproducts and density functional theory (DFT) computations to support the mechanistic interpretations.

## 2 Results and Discussion

### 2.1 Structural and Vibrational Characterization of Benzimidazole

Five unique prototropic tautomers of BzIm were found as minima on its potential energy surface (PES). According to the computations, the *1H*-BzIm (1*H*-benzimidazole) is the most stable by more than 130 kJ mol^–1^ (see Table 1). The aromatic arrangement of the π electrons in the two fused rings results in an increased stability of 1*H*-BzIm compared to that of the remaining forms. As discussed in the Supporting Information, the structural and spectroscopic characterization of the 1*H*-form of monomeric BzIm is vast and comprehensive.^38−41^ However, there is virtually no data available in the literature regarding other tautomeric forms of parent BzIm. The exceptions only briefly mention tautomerism of BzIm within the imidazole ring involving the N1, C2, and N3 atoms.^42,43^

**Table 1:**
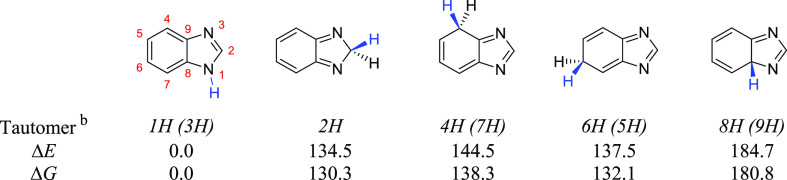
Structures, Relative Electronics (Δ*E*), and Gibbs Free (Δ*G*) Energies (at 298.15 K) Computed for the Prototropic Tautomers of Benzimidazole^a^

a Computed at the B97-1/def2-TZVP level of theory. Absolute computed energies (in Hartree) for the 1*H*-tautomer are *E*_h_ = −379.89895687 and *G* = −379.8122136. All relative energies (in kJ mol^–1^) are with respect to 1*H*-BzIm. Numbering of heavy atoms is shown in red for the 1*H*-tautomer. Numbering of all atoms (including H atoms) is shown in Figure S1. The labile H-atom is shown in blue.

b The names of symmetry-equivalent tautomeric forms are shown in parentheses.

**Figure 1:**
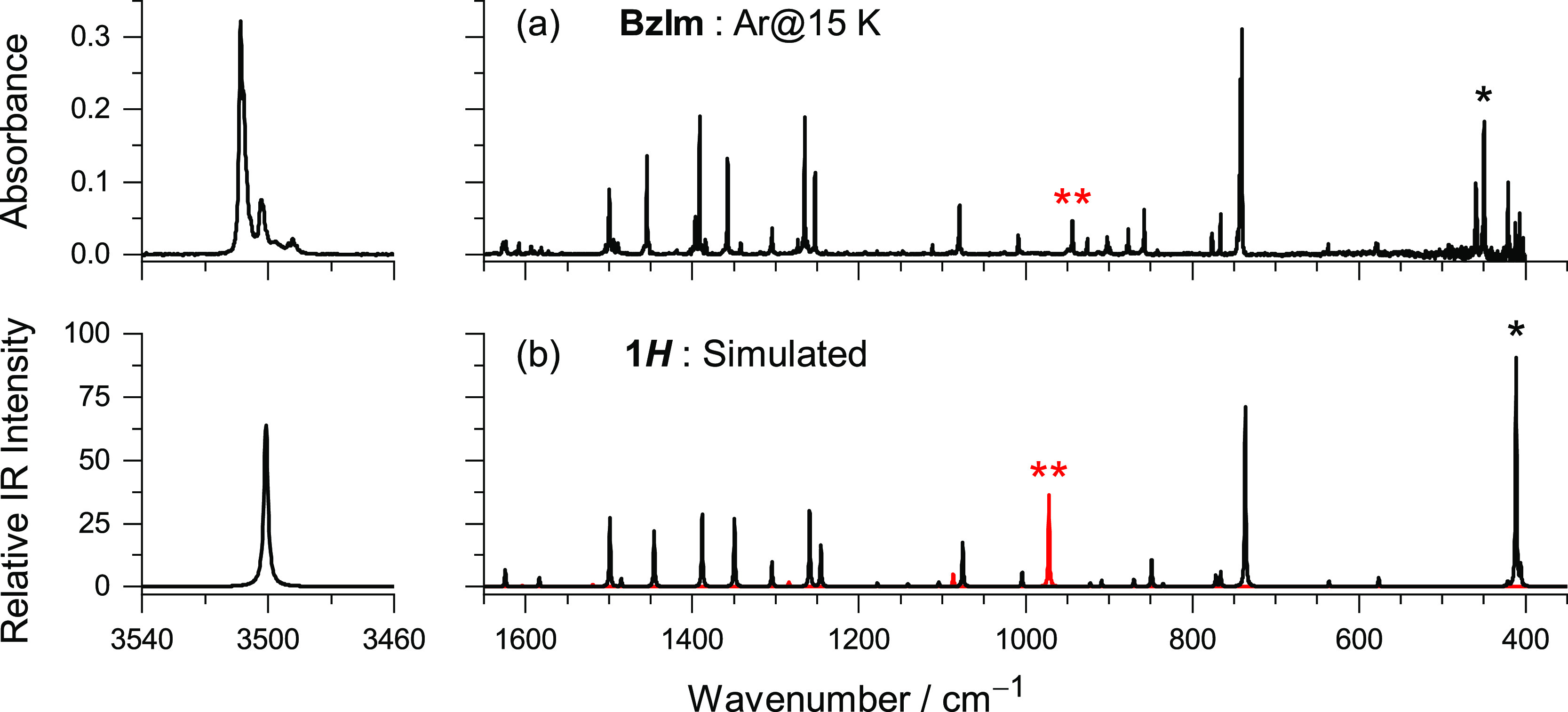
(a) Experimental IR spectrum of benzimidazole monomers isolated in an argon matrix at 15 K. (b) Black trace—simulated IR spectrum of 1*H*-BzIm computed at the B97-1/def2-TZVP level of theory in harmonic approximation. See the Experimental Section for details of simulation. See Figure S2 for the linear fit and determination of the scaling factor. Red trace—simulated spectrum of the first overtones (computed anharmonic frequencies unscaled). The bands marked with one and two asterisks (in both frames) are, respectively, assigned to the fundamental and the first overtone transitions of the NH out-of-plane bending γ(NH) mode.

Considering the relative stability of BzIm tautomers (Table 1), 1*H*-BzIm should dominate in the gas phase, and it should be the only form trapped from gas into a cryogenic matrix. Indeed, the three strongest bands found in the IR spectrum of BzIm isolated in an argon matrix (15 K) at 3509, 742/740, and 459/449 cm^–1^ are assigned to characteristic modes of the 1*H*-form, namely, to the NH stretching ν(NH), the in-phase CH out-of-plane bending γ_d_(CH), and the NH out-of-plane bending γ(NH), respectively (see Figure 1 and Tables S1 and S2). The overall IR spectrum of BzIm isolated in solid argon is in agreement with previously published spectroscopic data.^38,44^ Moreover, the experimental IR spectrum is very well reproduced by the theoretical spectrum of 1*H*-BzIm (see Figure 1 and Tables S1 and S2). The exception is a medium-intensity band found at 944 cm^–1^ (Figure 1a), not predicted by calculations in the harmonic approximation. This band should be assigned to the first overtone of the NH out-of-plane bending mode [2γ(NH)], for which the anharmonic calculation predicts a transition at 972 cm^–1^ with an IR intensity of 36.4 km mol^–1^ (Figure 1b). The observation in the IR spectra of similar overtone bands was previously reported for matrix-isolated indole^36^ [2γ(NH) = 804 cm^–1^] and 7-azaindole^37^ [2γ(NH) = 927 cm^–1^] and should be a common trend for aromatic molecules containing the NH group.

### 2.2 Fixed-Ring H-Scrambling Photochemistry of 1*H*-Benzimidazole

**Figure 2:**
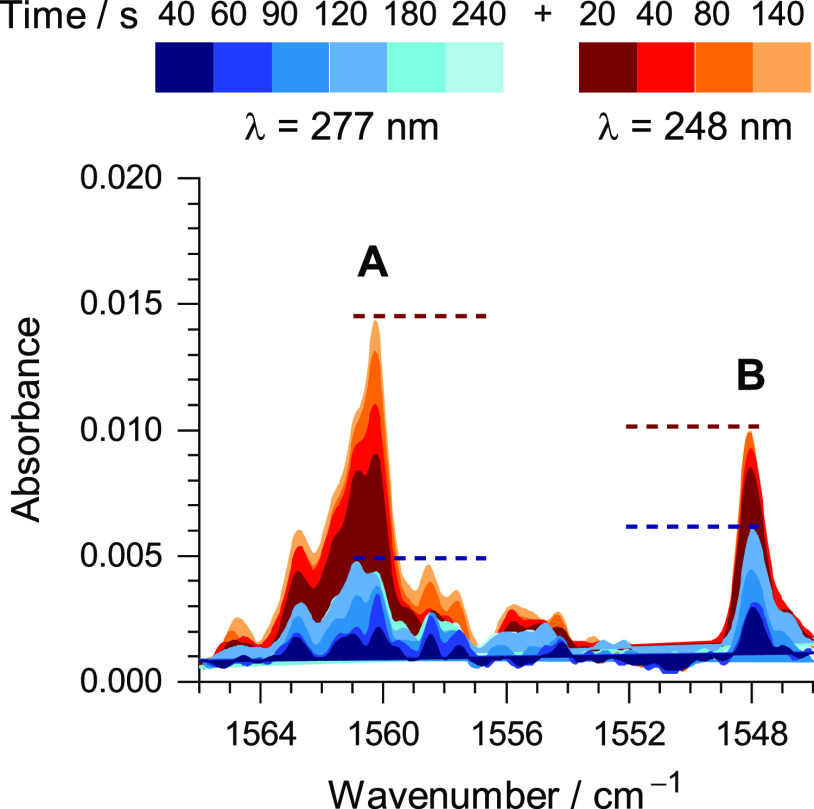
Changes of the IR spectra of BzIm isolated in an Ar matrix at 15 K during a sequence of irradiations, initially at λ = 277 nm (blue palette) and subsequently at λ = 248 nm (brown palette). The bands with maxima near 1560 and 1548 cm^–1^ were assigned to species **A** and **B**, respectively, photogenerated from 1*H*-BzIm. The dashed lines represent the peak intensities of **A** and **B** corresponding to two photostationary states.

The lowest energy spin-allowed electronic transition of 1*H*-BzIm has been assigned as a π* ← π transition, with origin at 36021.38 cm^–1^ (∼277.61 nm) in the gas phase.^41^ Initial irradiations of matrix-isolated benzimidazole were carried out at longer wavelengths, at 300 and 290 nm, but did not lead to any change in the IR spectrum. After narrow-band (fwhm = 0.2 cm^–1^) irradiations at λ = 280 nm, a trace amount of the 1*H*-BzIm form was consumed, and the transformation was barely discernible, but upon irradiations at λ = 277 nm, i.e., near the electronic origin of BzIm,^40−42,45−47^ it started to react more extensively (∼15% consumed in 140 s). Concomitantly, two sets of new bands emerged in the IR fingerprint region and could be assigned to two different products, **A** and **B**, because they exhibited different kinetic patterns of formation. These two species were found to be persistent under matrix-isolation conditions on the timescale of our experiments (hours). Two non-overlapping bands, characteristic of the new photoproducts, were found in the 1565–1540 cm^–1^ spectral range: at 1560 cm^–1^, assigned to **product A** and at 1547 cm^–1^, assigned to **product B** (Figure 2). Upon a few minutes of irradiation at λ = 277 nm, both the amount of consumed 1*H*-BzIm and the amounts of products **A** and **B** reach a plateau, suggesting the existence of a photo-stationary equilibrium among the three structures (see spectra in the blue palette in Figure 2). Upon subsequent irradiation of the matrix at λ = 248 nm (that corresponds to the second excited π–π* singlet state of 1*H*-BzIm, which was reported to have an energy of 5.0 eV in aqueous solution),^48^ the previous photo-stationary state is perturbed. Irradiations at 248 nm not only enhance the consumption of the 1*H*-form but also change the ratio of formation of the two photoproducts (**A/B**) (see Figure 2). Additionally, it was found that by irradiating freshly deposited matrices of BzIm at λ = 277 nm, the **A/B** ratio would be close to unity (47:53), whereas for initial irradiations at λ = 260 nm, the ratio shifted in favor of **A** (**A/B** = 78:22).

**Figure 3:**
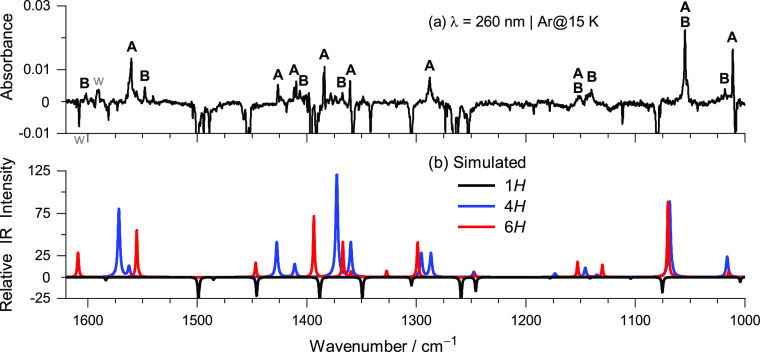
(a) Experimental difference IR spectrum showing changes upon UV irradiation at λ = 260 nm of BzIm isolated in an Ar matrix at 15 K. The downward bands are due to consumed species assigned to 1*H*-BzIm (truncated), and the upward bands are due to the photoproduced species **A** and **B**, distinguished based on further photo-transformations (see discussion below). The bands near 1600 cm^–1^ marked with w (dimmed) correspond to matrix-isolated monomeric water. (b) Simulated IR spectra of 1*H*-BzIm (black line), 4*H*-BzIm (blue line), and 6*H*-BzIm (red line) computed at the B97-1/def2-TZVP level of theory. The theoretical vibrational frequencies were scaled by 0.983, and the IR intensity of 1*H*-BzIm was multiplied by −1.

The remaining bands of products **A** and **B** were also distinguished based on their kinetics of growth upon ultraviolet irradiations. Subsequently, each set of bands was compared with putative photoproducts of 1*H*-BzIm, and a close agreement was found between the IR spectrum assigned to product **A** and that computed for 4*H*-benzo[*d*]imidazole (4*H*-BzIm), as well as between the IR spectrum assigned to product **B** and that computed for the 6*H*-benzo[*d*]imidazole (6*H*-BzIm)^49^ (Figure 3). The bands of products **A** and **B**, shown in Figure 2, were assigned to the vibrations of double bonds. The band of **A** centered at 1560 cm^–1^ was assigned to the [ν(C7=C8) + ν(C5=C6)] mode of 4*H*-BzIm, predicted to be its third most intense IR band (80 km mol^–1^). The band of **B** centered at 1548 cm^–1^ was assigned to the [ν(C5=C4) – ν(C9=N3) + ν(C2=N1)] mode of 6*H*-BzIm, predicted to be its second most intense IR band (56 km mol^–1^). Other characteristic bands of the newly identified benzimidazole tautomers were found at 1665 (6*H*-) and 1657 cm^–1^ (4*H*-) due to C7=C8 stretching modes, with computed counterparts at 1672 and 1663 cm^–1^, respectively. Notice that 1*H*-BzIm has its highest-frequency ν(CC) stretching mode at a much lower wavenumber (1627 cm^–1^). The characteristic bands due to the methylene scissoring modes δ(CH_2_) of the new tautomers were found at 1367 (6*H*-) and 1360 cm^–1^ (4*H*-), with nearly the same computed frequencies. The observed frequencies are in close agreement with those of previously identified δ(CH_2_) modes of two photogenerated thione isomers of matrix-isolated thiophenol at 1363 and 1389 cm^–1^.^50^ A detailed characterization of the IR spectra of 4*H*-BzIm and 6*H*-BzIm is provided for the first time in Tables S3 and S4, respectively. It is particularly noteworthy that for each of the two new tautomers, all the vibrational modes in the 1700–600 cm^–1^ range with predicted IR intensities over 10 km mol^–1^ (non-overlapping with 1*H*-BzIm) have been identified in the experiment.

Finally, it is also worth mentioning that there was no evidence of the formation of 2*H*-BzIm throughout the experiments. For example, the absence of a photoproduct band around 750 cm^–1^, where the most intense IR mode of 2*H*-BzIm is predicted, in a spectral region free of bands of either the precursor 1*H*-BzIm or photoproducts 4*H*-BzIm and 6*H*-BzIm suggests that 2*H*-BzIm is not a product of benzimidazole photochemistry in an argon matrix.

### 2.3 Fixed-Ring Photochemistry of 4*H*- and 6*H*-Tautomers

Tautomers 4*H*- and 6*H*- of benzimidazole contain localized and conjugated double bonds, unlike the aromatic 1*H*-form, and should therefore possess electronic transitions at longer wavelengths because conjugation of double bonds leads to reduction of the band gap for π* ← π transitions. In fact, time-dependent DFT (TD-DFT) calculations reveal that 4*H*- and 6*H*-forms should absorb light at wavelengths longer than 1*H*-form absorbs. Intense electronic π* ← π transitions were predicted at 335 nm for 6*H*-BzIm and at 349 nm for 4*H*-BzIm, at much longer wavelengths than the corresponding computed lowest–energy transition of 1*H*-BzIm at 247 nm (see Figure S3 and Table S5).

**Figure 4:**
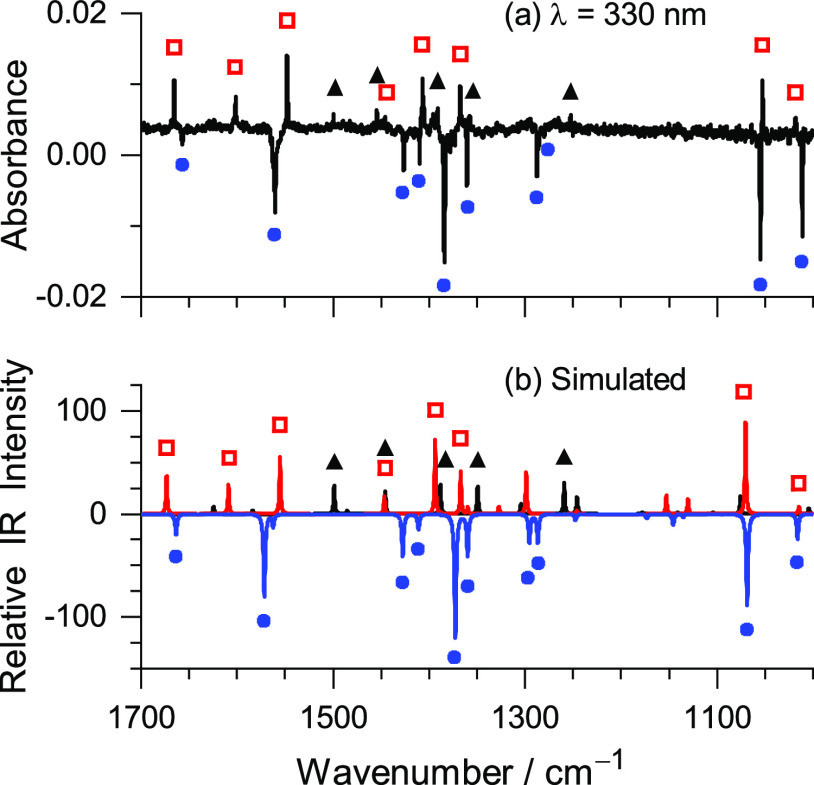
(a) Experimental difference IR spectrum showing changes upon UV irradiation at λ = 330 nm, subsequent to the initial irradiation at λ = 260 nm of BzIm isolated in an Ar matrix at 15 K. The downward bands are due to consumed 4*H*-BzIm (blue circles); the upward bands are due to photoproduced 6*H*-BzIm (red squares) and 1*H*-BzIm (black triangles). (b) Simulated IR spectra of 1*H*-BzIm (black line), 4*H*-BzIm (blue line), and 6*H*-BzIm (red line) computed at the B97-1/def2-TZVP level. The theoretical vibrational frequencies were scaled by 0.983, and the IR intensity of 4*H*-BzIm was multiplied by −1.

The initial UV irradiations of 1*H*-BzIm were carried out using different laser wavelengths yet always in the λ < 280 nm range, where the 1*H*-tautomer absorbs UV light. Aiming at further photochemical transformations of 4*H*- and 6*H*-forms, generated herein for the first time, the matrix was subjected to a series of narrow-band irradiations at longer wavelengths, where 1*H*-BzIm does not absorb. The initial wavelength was set at λ = 400 nm, and then, the wavelengths of subsequent irradiations were decreased in steps of 10 nm. Upon the irradiation at 380 nm, the IR intensity of the bands assigned to 4*H*-BzIm decreased, and the IR intensity of the bands assigned to 6*H*-BzIm simultaneously increased. A careful analysis shows that 1*H*- was also partially repopulated at the cost of 4*H*-BzIm, which is evidence that indeed, species **A** and **B** are isomers of 1*H*-BzIm, rather than its decomposition products. Later, it was found that upon irradiation at λ = 260 nm, the 4*H*-tautomer would be repopulated, and this cycle could be repeated multiple times. The consumption/repopulation cycle of 4*H*- was repeated, while reducing the wavelength of irradiation used to consume 4*H*- (below 380 nm). The most efficient consumption of 4*H*- occurred upon irradiations at λ = 330 nm, where a 30 s irradiation was enough to totally consume it (see Figure 4).

Upon total consumption of 4*H*- and enrichment of the matrix with 6*H*-, a search was conducted to find out which wavelengths allow its selective consumption. Narrow-band irradiation at λ = 283 nm, i.e., at an energy slightly below that of the electronic origin of 1*H*-, led to the consumption of 6*H*- and major recovery of 1*H*-. Importantly, signs of the formation of 4*H*- were also found (see Figure S4). These results are in line with the TD-DFT computations, which predict 6*H*- to have an allowed transition (*f* = 0.0820) at 260 nm, a wavelength longer than that of the first spin-allowed transition of 1*H*- computed at 247 nm.

### 2.4 Ring-Opening Photochemistry of the 1*H*-Benzimidazole Tautomer

**Figure 5:**
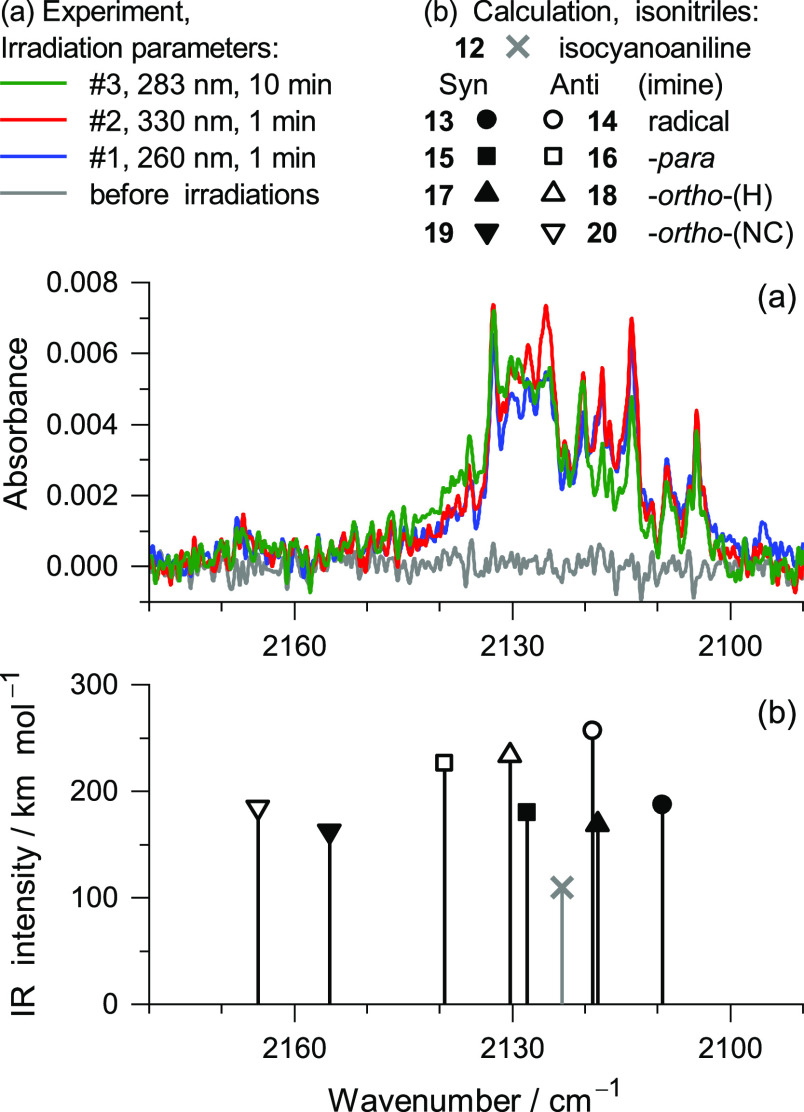
(a) Characteristic fragment of the IR spectra of benzimidazole isolated in an Ar matrix at 15 K: before irradiations (gray line) and after UV irradiations at 260 nm (blue line), 330 nm (red line), and 283 nm (green line). (b) B97-1/def2-TZVP-computed harmonic wavenumbers and IR intensities for putative isocyano photoproducts. Respective structures are shown in Chart 2. The computed frequencies were scaled as discussed in the Computational Section.

Upon the initial irradiations of matrix-isolated BzIm at wavelengths below 280 nm, a set of new low-intensity bands was found in the 2200–2000 cm^–1^ frequency range. This region is characteristic of stretching vibrations of molecular fragments containing either triple bonds or cumulated double bonds (e.g., nitrile, isonitrile, ketene, and allene, among others) and therefore could not be ascribed to either 4*H*-BzIm or 6*H*-BzIm. The multiplet feature shown in Figure 5 appears during the first instants of irradiation (1 min at 260 nm), and from that point on, it remains always present, after any UV irradiation, contrasting with the kinetics of growth and disappearance of isomers 4*H*-BzIm and 6*H*-BzIm, both of which ultimately disappear upon irradiations at 283 nm.

**Figure 6:**
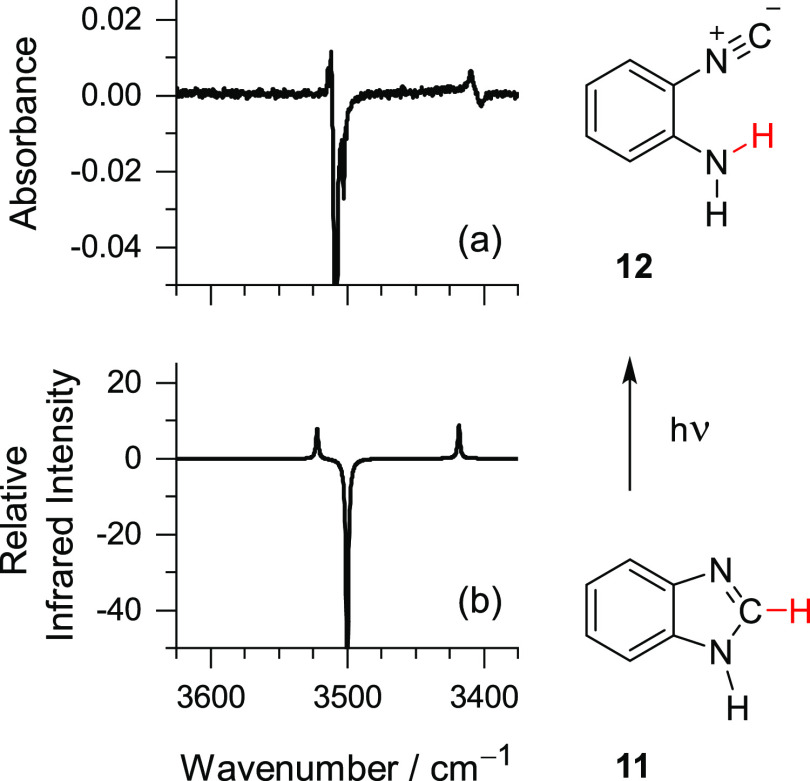
(a) Changes in the spectrum of matrix-isolated BzIm after 2 min of irradiation at λ = 248 nm. The negative (truncated) band is due to the BzIm precursor. Positive bands are due to the photoproduct. (b) Simulated IR difference spectrum computed at the B97-1/def2-TZVP level. The positive bands are due to ICA **12** (note that computed IR intensities of **12** were scaled by 0.25, which makes it clear that conversion from **11** to **12** is not quantitative). The negative (computed IR intensity scaled by −1, truncated) band is due to 1*H*-BzIm **11**. All computed frequencies in this range were scaled by 0.955.

**Chart 2:**
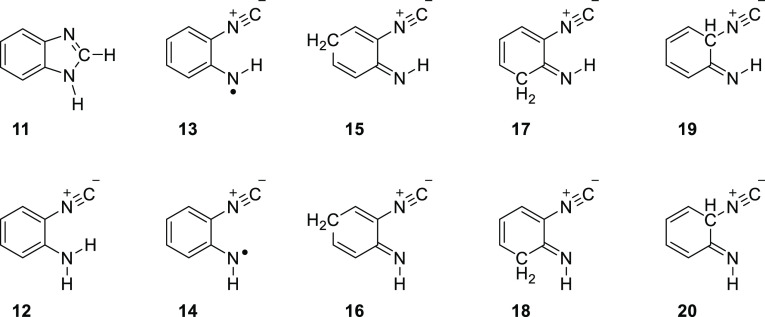
Structures of Benzimidazole and its Isonitrile (Isocyano) Derivatives

In the context of the current investigation, it should be noticed that isocyano (−N≡C) compounds could be produced upon cleavage of the N1–C2 bond (and simultaneous cleavage of the C2–H bond). The NC stretching mode of the −N≡C group has been found for other compounds, either matrix-isolated or in solutions, to give rise to sharp IR bands in the 2160–2100 cm^–1^ range.^51−54^ The band shown in Figure 5, on the other hand, appears broad and with a complex multiplet structure centered around 2120 cm^–1^ (spanning about 40 cm^–1^, see Figure 5), suggesting the contribution of multiple isocyano species. The ν(−N≡C) stretching mode is characterized by intrinsically high absorption coefficients in IR (hundreds of km mol^–1^).^35,54,55^ Yet the components of the broad multiplet band around 2120 cm^–1^ were found with low peak IR absorbance (between 4 × 10^–3^ and 8 × 10^–3^). These factors make the unequivocal assignment of the photoproducts responsible for such absorptions difficult because the IR bands due to other vibrations (of the photoproducts carrying the isocyano group) would have too low intensities to be detected. Nonetheless, as justified below, we propose that 2-isocyanoaniline (ICA) **12** (see Chart 2) should be formed, upon concerted cleavage of the N1–C2 bond in 1*H*-BzIm **11** and [1,2]H-shift from the C2–H group to the N1 atom. The changes in the NH stretching range of the spectra, consistent with formation of ICA, are presented in Figure 6.

The new bands appearing at 3511 and 3409 cm^–1^ (Figure 6a) have a good match with the theoretically predicted bands of ICA at 3522 and 3418 cm^–1^ (Figure 6b), ascribed to the antisymmetric and symmetric NH_2_ stretching modes of ICA, respectively. The absolute positions of these bands in the IR spectrum, and separation of an approximately 100 cm^–1^, are in good agreement with the respective values reported for the NH stretching modes of the parent aniline^56−59^ and 2-cyanoaniline^60^ in the gas-phase jet-cooled molecular beam and in cryogenic matrix. Note that conversion of 1*H*-BzIm **11** to ICA **12** can be only partial because it occurs simultaneously with the conversion of 1*H*-BzIm into its tautomers 4*H*-BzIm and 6*H*-BzIm, as discussed earlier in Sections 2.2 and 2.3. This is in accordance with the estimate that around 20% of consumed **11** is converted to **12**. The spectral manifestations of ICA in the fingerprint range are difficult to confirm or deny because this spectral range is congested, and the bands of **12** are predicted at close positions as the bands of precursor **11**. The non-accumulation of **12** in the cryogenic matrix can be explained by the fact that the wavelengths required to induce the UV reactions of **11** (277 nm or shorter) must be simultaneously acting on photogenerated **12**, leading to its subsequent phototransformations (see below). The ν(−N≡C) stretching mode in ICA is computed at 2123 cm^–1^ (109.6 km mol^–1^, see Figure 5b), near the experimental value reported for phenylisocyanide at 2125 cm^–1^.^61^

We propose that N–H homolytic cleavage in the amino group of **12** shall give rise to the 2-isocyanoanilinyl radical (**13**, **14**, see Chart 2) and an H atom. Note that two radical isomers (*syn***13** or *anti***14**), differing by the orientation of the imino group relative to the isocyano group, could be produced upon the N–H bond cleavage in the amino group. Furthermore, recombination of the H atom with **13** or **14** at para or ortho positions with respect to the imino group^62^ shall give rise to three pairs of *syn* or *anti* isomers (see Chart 2), namely, 2-isocyanocyclohexa-2,5-dienimine (*syn***15** or *anti***16**), 2-isocyanocyclohexa-2,4-dienimine (*syn***17** or *anti***18**), or 6-isocyanocyclohexa-2,4-dienimine (*syn***19** or *anti***20**). This hypothesis is in line with the fact that the band observed at 2140–2100 cm^–1^ shows overlapping absorptions of multiple species. Interestingly, the computed IR frequencies of the ν(−N≡C) modes for the mentioned candidate photoproducts span from 2165 to 2109 cm^–1^, corroborating the proposed interpretation. Further insights into the mechanism underlying such reactivity are discussed below.

### 2.5 Mechanistic Analysis of the Fixed-Ring Photochemistry

The pivotal role of the optically dark σ* ← π excited states in the photochemistry of heteroaromatic compounds containing endocyclic NH groups, such as pyrrole, imidazole, or indole, was recognized theoretically and presented as a new paradigm in photochemistry of aromatic biomolecules by Sobolewski *et al.*(63) The σ* ← π states are characterized by a repulsive PES along the NH bond stretching and might lead to the homolytic cleavage of the hydrogen atom from the NH bond. Predictions of Sobolewski and co-authors received experimental verification by photofragment translational spectroscopy, *via* the detection of fast detached hydrogen atoms from UV-irradiated azoles in the gas phase.^64−67^ Experimental and theoretical investigations have also reported the importance of σ* ← π states in the photochemistry of imidazole,^63,68−71^ which, in fact, is the five-membered ring contained in benzimidazole. Concomitantly, for several aromatic azoles isolated in cryogenic matrices, the formation of the corresponding radicals upon UV-induced detachment of the H-atom has been observed.^72−74^ Interestingly, the formation of unusual tautomers for these matrix-isolated molecules does not require the presence of intramolecular H-bonds but instead results from the recombination of the released H-atom and its complementary radical in positions far from those where the initial dissociation took place.^36,37^ Such a process is facilitated in cryogenic matrices due to the confinement of both fragments constituting the radical pair produced in the same matrix cavity (cage effect)^75^ and has been coined by Sobolewski *et al.* as photoinduced dissociation association (PIDA).^75,76^

**Figure 7:**
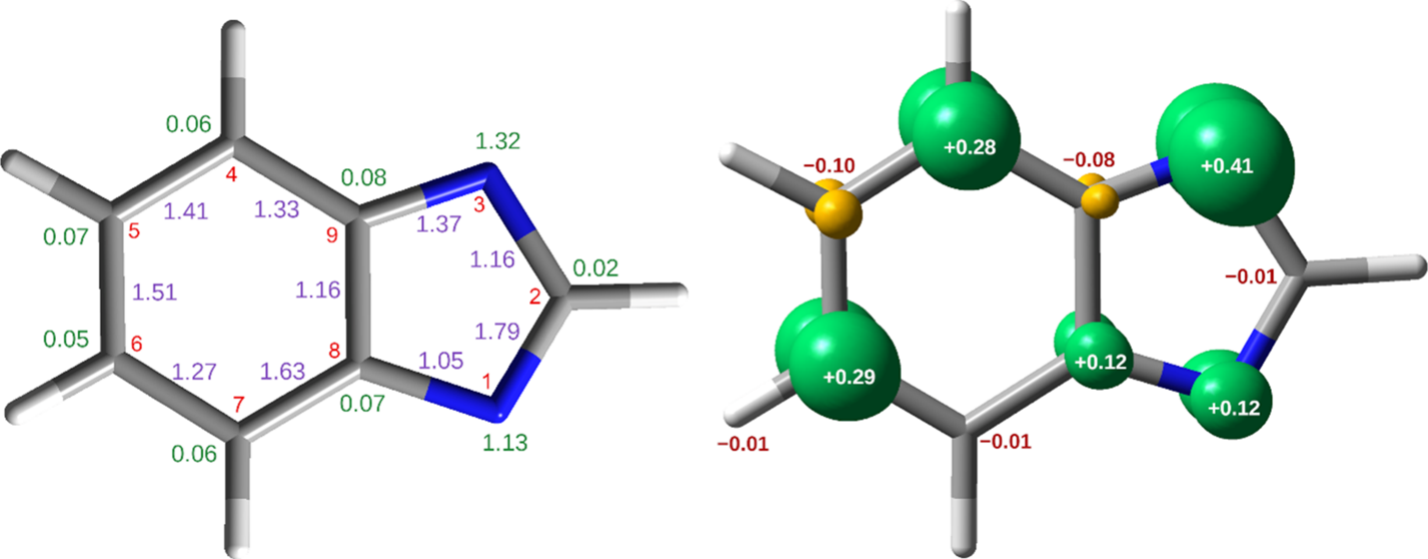
Left: structure of the benzimidazolyl radical optimized at the UB97-1/def2-TZVP level showing NRT bond orders (purple, only values higher than 1.0 included) and non-bonding orbital populations (green, only values higher than 0.02 included). Numbering of the heavy atoms is shown in red. Right: spin density surface (isovalue ± 0.01 e) for the benzimidazolyl radical. Green color stands for alpha spin density (“+” sign) and yellow for beta spin (“–” sign). Element colors: H—white; C—gray; and N—blue. The values near the heavy atoms correspond to the calculated atomic natural spin density values.

On the basis of the aforementioned, it is likely that the photochemistry reported in Sections 2.2 and 2.3, involving a dynamic photoequilibrium among three benzimidazole tautomers (1*H*-, 4*H*-, and 6*H*-), occurs *via* the PIDA mechanism and that the benzimidazolyl radical (Figure 7), formed upon homolytic cleavage of the NH bond in 1*H*-BzIm, plays a key role in such processes. This reactivity will from now on be referred to in this work as *fixed-ring photochemistry*. The benzimidazolyl radical contains an unpaired alpha electron (doublet multiplicity) and belongs to the *C*_s_ symmetry point group. There is no principal axis of rotation intercepting the C2H bond (Figure 7, left). According to the natural resonance theory (NRT) analysis of the benzimidazolyl radical, C2N3 becomes essentially a single bond (NRT bond order of 1.16), whereas the N1C2 bond in the benzimidazolyl radical takes a character close to that of a double bond (NRT bond order of 1.79). Similar NRT bond orders, essentially a double N1C2 bond and a single C2C3 bond, were found for the indolyl and 7-azaindolyl radicals.^36,37^ Natural bond orbital (NBO) analysis of the benzimidazolyl wavefunction showed that most of the unpaired alpha spin density is centered around the N3 (+0.41 *e*), C6 (+0.29 *e*), C4 (+0.28 *e*), N1 (+0.12 *e*), and C8 (+0.12 *e*) atoms (see Figure 7, right). According to NRT analysis (Figure S5), which decomposes a wavefunction into the Lewis structure contributors, the resonance structures with the largest weights have the radical center located mainly on the C4 (14.2%), N3 (12.6%), C6 (7.0%), and N1 (4.1%) atoms. It is likely that initially detached H-atoms recombine with the corresponding benzimidazolyl radical at those positions with higher spin density. Precisely such type of reactivity was experimentally observed for indole^36^ and a series of substituted indoles,^37,73,74^ where the new 3*H*-tautomers were experimentally detected, in accordance with the largest predicted spin density of the respective radicals at the C3 ring position. Recombination of the H-atom at either N3 or N1 of the benzimidazolyl radical would lead to two equivalent isomers, resulting in the back-formation of the precursor 1*H*-BzIm. However, recombination at C4, C6, or C8 should lead to the formation of 4*H*-, 6*H*-, and 8*H*-, respectively. Indeed, as shown in Sections 2.2 and 2.3, 4*H*- and 6*H*- are two new prototropic tautomers of benzimidazole which were generated photochemically and successfully identified in this work for the first time.

**Figure 8:**
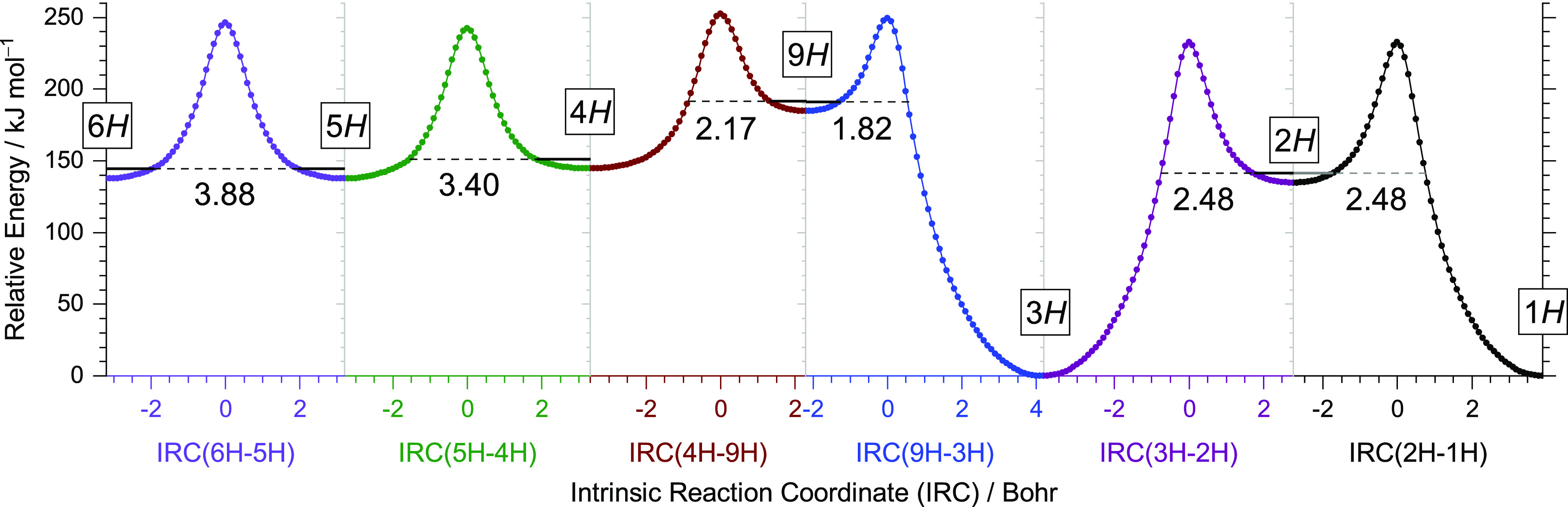
Potential energy profiles along the intrinsic reaction coordinates (IRCs) for the *H*-shifts between neighboring heavy atoms in the benzimidazole system computed at the B97-1/def2-TZVP level in non-mass-weighted (Cartesian) coordinates. The absolute electronic energy of the 1*H*-BzIm tautomer was taken as the relative zero. Horizontal solid lines represent the zero-point vibrational energy (ZPVE) levels of the higher-energy tautomer in each pair. Dashed lines represent the barrier widths for H-atom tunneling at the ZPVE level. See Table 1 for the graphical representation of the prototropic tautomers and for their energies.

The mere fact of existence as a local minimum on a PES does not guarantee, by itself, a successful capture of a molecular species or its subsequent characterization by stationary spectroscopy, even under cryogenic and inert conditions. It is especially difficult to stabilize those species that can relax to more stable isomers through reactions involving hydrogen atoms, which are prone to undergo quantum tunneling.^77^ This is the case of 8*H*-BzIm/9*H*-BzIm, which must be considered fleeting species due to *tunneling instability*.^78^ According to computations, a barrier height of 58.15 kJ mol^–1^ and a width of 1.82 Bohr separates 8*H*- from the most stable 1*H*-tautomer (the same applies for the symmetrically equivalent 9*H*- and 3*H*-pair of tautomers; see Figure 8). Shall 8*H*- (9*H*-) be formed, it will quickly disappear, on the millisecond time scale, back to 1*H*- (3*H*-) BzIm through quantum tunneling (see Table S6 for estimations of rate constants). On the other hand, tautomers 4*H*- and 6*H*- are located in much deeper wells (Figure 8), and according to quantum tunneling estimations (Table S6), once formed, they shall remain in the matrix unchanged, as in fact observed. 2*H*-BzIm, which throughout the experiments was never detected, has a relative stability between that of 8*H*- and that of 4*H*- and 6*H*-. A barrier height of 90.94 kJ mol^–1^ and a width of 2.48 Bohr separates 2*H*- from the most stable 1*H*-tautomer, rendering it an estimated half-life time of 14 h, which should allow its detection in case it is formed. However, the spin density analysis of the benzimidazolyl radical explains why 2*H*-BzIm shall not be produced: the unpaired spin density at C2 is very low, making recombination of the radical pair at C2 unlikely.

### 2.6 Mechanistic Analysis of the Ring-Opening Photochemistry

As shown in Section 2.4, the photochemistry of benzimidazole is not limited to the photoequilibrium among tautomers 1*H*-, (3*H*-), 4*H*-, and 6*H*-. The photoproducts absorbing at 2200–2000 cm^–1^ must be formed through some other mechanism. One of such mechanisms would be the cleavage of the N1–C2 bond, occurring upon photoexcitation of benzimidazole, giving rise to open-ring photoproducts.^79^ Cleavage of an X1–C2 bond in azoles is not unprecedented.^35,51^ It is, for instance, the main reactive channel in benzoxazole, which upon UV irradiation, undergoes a concerted O1–C2 cleavage and [1,2] H-shift from C2 to O1 to yield the open-ring 2-isocyanophenol.^35^ By analogy, the photochemistry discussed below will be referred to as *ring-opening photochemistry*.

**Figure 9:**
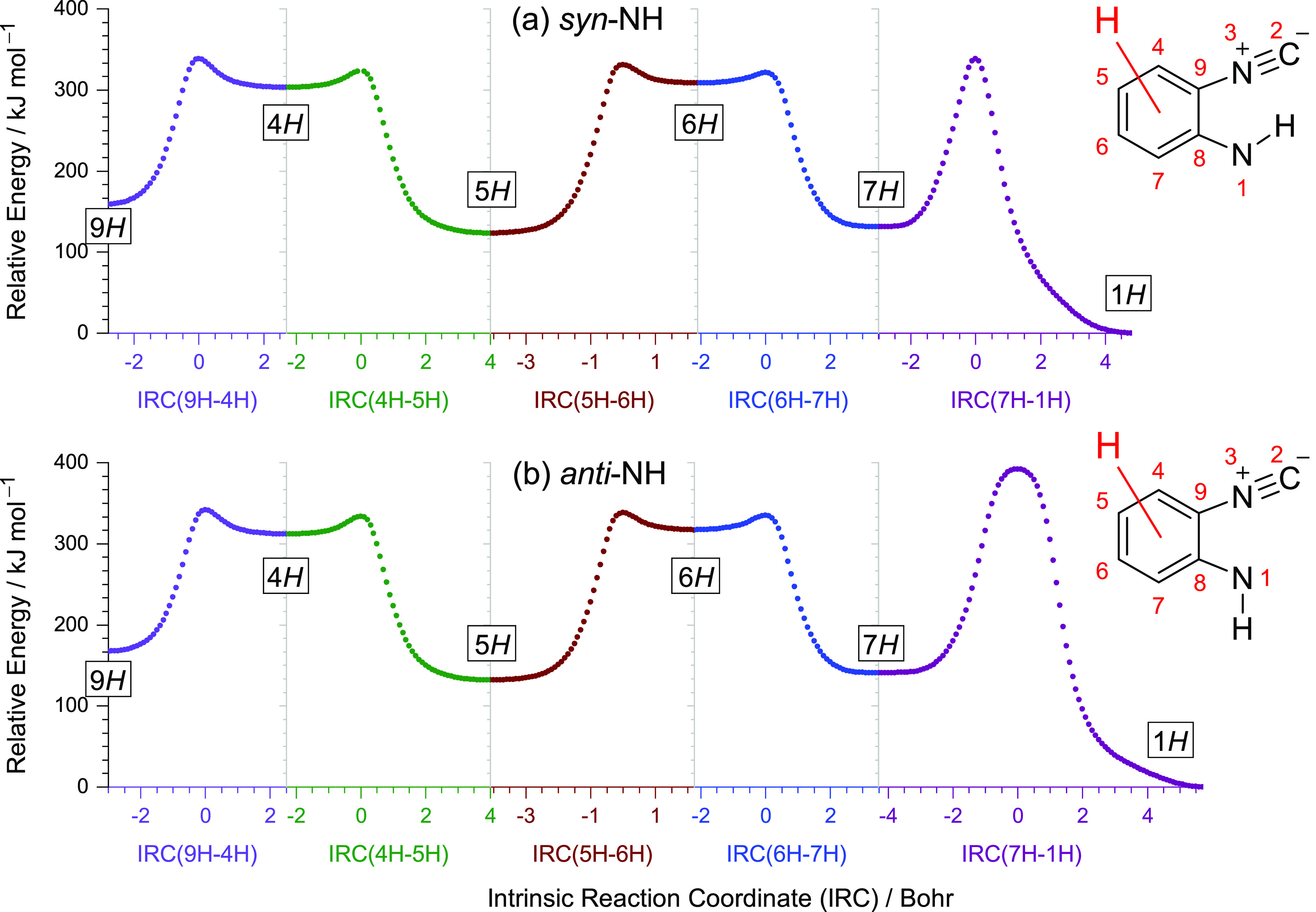
IRC profiles for the H-shifts around the six-membered ring (the migrating H atom is marked in red) computed at the B97-1/def2-TZVP level in non-mass-weighted (Cartesian) coordinates. The energy of 1*H* was set as the relative zero. The *syn*-NH and *anti*-NH imino isomers of 2-isocyanoaniline are shown near the IRC profiles, along with the atom numbering. The ring positions 4-, 6-, 7-, and 9- correspond to the meta-(NC), meta-*H*, ortho-*H*, and ortho-(NC) positions relative to the NH group, respectively. The structures and energies of the isomers resulting from recombination of an H-atom with radicals **13** and **14** at different ring positions are summarized in Table S7.

The concerted H-shift from C2 to N1, along with the N1—C2 bond cleavage, would lead to ICA (see Figure 6). King, Oliver, and Ashfold reported a comprehensive study of the mechanisms of H atom loss in the parent aniline (C_6_H_5_NH_2_), using H-atom photofragment translational spectroscopy,^80^ following excitation in the broad range of UV wavelengths (294 nm > λ_phot_ > 193 nm), accompanied with formation of the anilinyl radical in the ground electronic state. Furthermore, the electronic origin of the S_1_ excitation in 2-cyanoaniline (the cyano isomer of **12**) was reported at 31270 cm^–1^ = 319.8 nm^81^ or at 31265 ± 2 cm^–1^.^82^ Therefore, it is likely that also ICA **12** should absorb and undergo NH bond fission at irradiation wavelengths such as 277 nm (or shorter) used in this work to excite BzIm. Hence, the corresponding 2-isocyanoanilinyl radical (*syn***13** and *anti***14**, see Chart 2) should, accordingly, be generated in the matrix, along with an H atom. NBO analysis of the corresponding 2-isocyanoanilinyl radicals **13** and **14** revealed that the largest spin density is located at the N1, C5 (*para*), C7 (*ortho*-H), and C9 (*ortho*-NC) atoms (Figure S8), and the NRT analysis corroborated such interpretation (Figure S5). Recombination of the radical pair at the nitrogen atom would lead back to ICA **12**, while recombination at the ring carbon atoms would yield its imino isomers **15–20** (see Chart 2). These tautomers are located in deep PES wells, separated from other forms by high barriers (over 170 kJ mol^–1^, Figure 9). The tautomers with the H atom attached in *meta* position relative to the imino group must be metastable and relax to the more stable neighboring ortho- or para-isomers through barriers as low as 20 kJ mol^–1^ (Figure 9).

The IR signatures of imino tautomers **17** and **18** with a labile H-atom attached at the *ortho*-(H) position (C atom bearing the H atom) and tautomers **15** and **16** at the *para*-position seem to be the most consistent with the IR signature of the irradiated matrix (see Figure 5). These are the most stable imino tautomers of ICA, having relative energies near 120–140 kJ mol^–1^ (see Table S7). Notice the similarity between the amino–imino tautomerism reported herein and the hydroxy–oxo tautomerism reported elsewhere. Namely, there is a direct analogy of the present case with previous observations of the UV-induced photochemistry of substituted phenols, where the H-atom detached from the OH group recombined precisely at *ortho*-(H) and *para*-positions of the ring, yielding the carbonyl (C=O) isomers or their derivatives.^50,54^

**Scheme 4:**
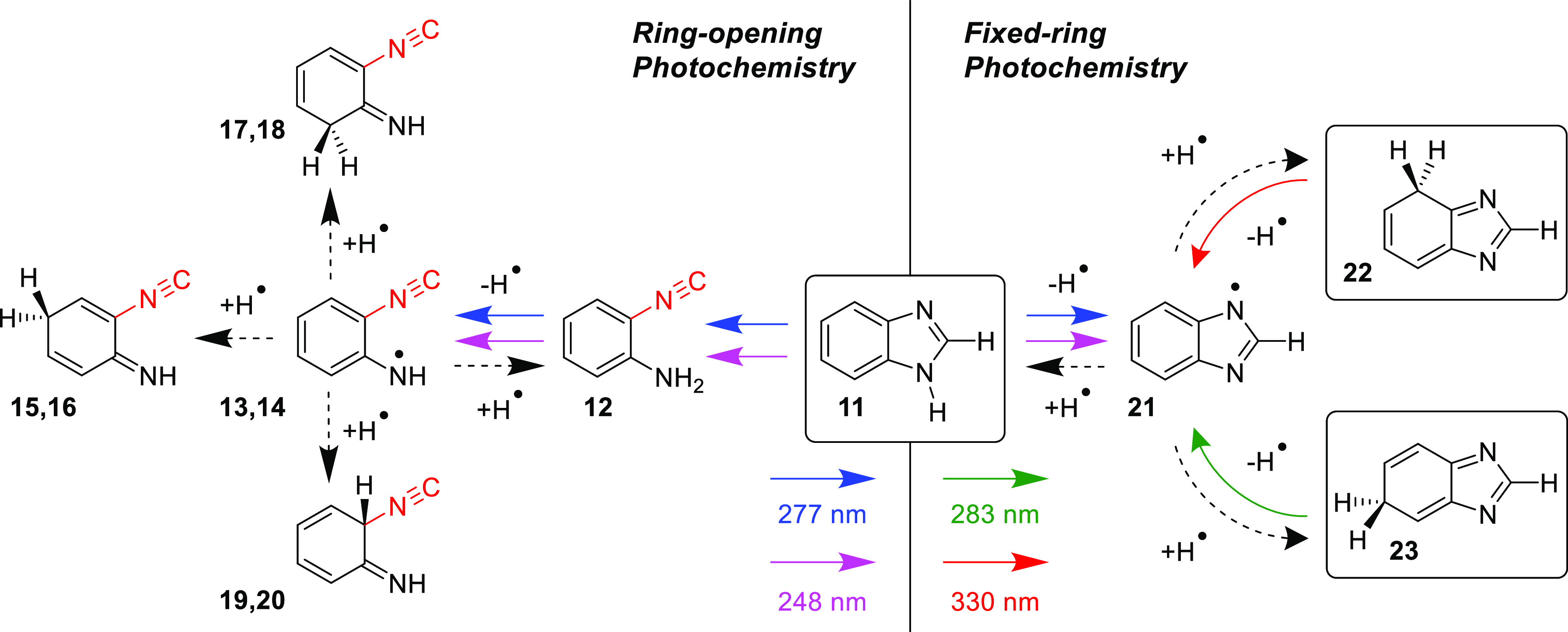
Summary of Dominant Concurrent Photochemistries of Monomeric Benzimidazole Isolated in an Argon Matrix at 15 K Molecules **12–20** in the left panel (“ring-opening”), with the isocyano groups shown in red, were identified as *a family*; the two molecules in the right panel (“fixed-ring”) surrounded by rectangles, **22** and **23**, were characterized by at least 15 IR bands each. Note color codes for irradiations at different wavelengths. The black dashed arrows correspond to the recombination of the radical pairs.

It is also interesting to compare approximate amounts of the photoproducts formed in the two main photochemical pathways of BzIm. The band in the 2140–2100 cm^–1^ range was integrated and then normalized by the average value of the computed IR intensities of the ν(−N≡C) mode of photoproducts **12–18**. Likewise, the characteristic bands at 1560 cm^–1^ (4*H*), 1548 cm^–1^ (6*H*), and 1054/1052 cm^–1^ (4*H* + 6*H*) were integrated and subsequently normalized by the respective computed IR intensities of the 4*H* and 6*H* tautomers. According to the analysis of the normalized integrated intensities, we concluded that the ring-open *versus* fixed-ring photoproduced isomers of benzimidazole, corresponding to the two photochemical pathways (Scheme 4), are generated in approximately equal amounts.

## 3 Conclusions

The UV-induced photochemistry of monomeric benzimidazole **11** isolated in an argon matrix at 15 K was discovered here to occur *via* two distinct mechanisms with similar yields. The fixed-ring reactivity initiated by irradiations at λ < 280 nm was characterized by detachment of the labile H atom from the endocyclic NH bond of 1*H*-BzIm, leading to the intermediacy of the benzimidazolyl radical **21** plus H-atom radical pair, which promptly recombined to yield tautomers 4*H*- (**22**) and 6*H*- (**23**), as shown in Scheme 4 (right). The characterization of hitherto unobserved tautomers 4*H*- and 6*H*- was achieved through their selective consumption by irradiations at λ = 330 and 283 nm, respectively. Indirect proof of intermediacy by radical **21** is given by NBO analysis, which places most of the spin density on N3 (+0.41), C4 (+0.28), and C6 (+0.29) atoms. Concomitantly, the initial irradiations at λ < 280 nm led to the formation of a family of compounds bearing the isocyano functional group, namely, ICA **12**, which is formed by ring opening of the imidazole moiety coupled with an H-shift from C2 to N1 and also the products of its chemistry **13–18** (see Scheme 4, left).

Interestingly, the photochemistry of monomeric benzimidazole bears no resemblance to its photochemistry in condensed phases^24,25^ but instead has a dual character in contrast to the photochemistry of indole (exclusively fixed-ring) and benzoxazole (exclusively ring-opening),^35,36^ with both of which it shares structural similarities (Chart 1). The observations reported here establish a direct correlation between structural aspects and chemical reactivity and show that neither of the two competitive pathways is surpassed but rather that both co-exist in benzimidazole.

## 4 Experimental Section

Benzimidazole (**1**) was acquired commercially from Alfa Aesar (99% purity). The sample of benzimidazole was placed in a home-made oven that was then connected to a closed-cycle helium cryostat (Advanced Research Systems, DE-202 expander). The sample was purified from volatile impurities by pumping through the cryostat at room temperature using a turbomolecular pump. Monomeric matrices were prepared by allowing the co-deposition of sublimated benzimidazole, whose vapor pressure was thermally enhanced in the oven, and a large excess of argon gas (N60, Air Liquide) onto a cryogenic CsI optical window (*T* = 15 K). The temperature of the CsI window was measured directly using a silicon diode sensor, connected to a digital controller (Scientific Instruments, model 9650-1), and stabilized with an accuracy of 0.1 K.

IR spectra were recorded using a Thermo Nicolet 6700 Fourier-transform IR (FTIR) spectrometer, equipped with a deuterated triglycine sulfate (DTGS) detector and a KBr beam splitter. The spectra were recorded, in the 4000–400 cm^–1^ range, with a resolution of 0.5 cm^–1^. To avoid interference from atmospheric impurities, a stream of dry and CO_2_ filtered-off air was continuously purged through the optical path of the spectrometer.

Ultraviolet (UV) irradiations were performed using a frequency-tunable narrowband pulsed light [full width at half-maximum (fwhm) of 0.2 cm^–1^, pulse energy ∼ 1 mJ], provided by a frequency-doubled signal beam of an optical parametric oscillator (Spectra Physics Quanta-Ray MOPO-SL) pumped using a pulsed Nd:YAG laser (Spectra Physics PRO-230: output power 4.3 W; pulse duration = 10 ns; repetition rate = 10 Hz). The matrices were irradiated through an outer quartz window of the cryostat, and after each irradiation, the transformations were monitored by collecting IR spectra.

## 5 Computational Section

Geometry optimizations and harmonic frequencies and IR intensities of BzIm and the putative photoproducts were computed at the B97-1/def2-TZVP level of theory^83,84^ using the default parameters of the GAMESS software package.^85^ The method was chosen due to its accuracy at predicting IR spectra of small organic molecules, as shown in a benchmark study of Kesharwani *et al.*(86) The absolute computed energies of the optimized structures are listed in Table S8, and the respective Cartesian coordinates are collected in Table S9. The nature of each stationary point (as a minimum or a first-order transition state) was inspected by the analysis of its Hessian matrix. The harmonic vibrational frequencies computed at the B97-1/def2-TZVP level for 1*H*-BzIm were subjected to least-squares linear fit against the experimental frequencies (for BzIm monomers isolated in an argon matrix) in the fingerprint (1700–400 cm^–1^) range, resulting in a scaling factor of 0.983 (see Figure S2a). This factor, in the fingerprint range, was used to scale the harmonic vibrational frequencies for their subsequent comparison with the experimental wavenumbers, to account for the neglected limitations of the implemented methods and basis set. It is also interesting to note that another method typically used for computational purposes in the previous studies of our group, B3LYP/6-311++G(d,p), gives exactly the same scaling factor of 0.983 (see Figure S2b) in this spectral range.^35,54^ The harmonic vibrational frequencies in the 3500–2800 and 2300–2000 cm^–1^ ranges were scaled by factors of 0.955 and 0.974, respectively, derived in our previous work for structurally similar compounds (BzOx, 2-isocyanophenol, and 2-cyanophenol).^35^ The scaled harmonic wavenumbers and absolute IR intensities were used to simulate theoretical IR spectra, by convoluting each peak with a Lorentzian function having an fwhm of 1 cm^–1^, using the Chemcraft 1.8 software package.^87^ Anharmonic vibrational computations for BzIm were carried out at the B97-1/def2-TZVP level of theory, using the fully automated second-order vibrational perturbation theory (VPT2) approach of Barone *et al.*(88−90) as defined in the Gaussian 16 (G16) software package.^91^ The G16 computations were performed using tight optimization criteria, the ultrafine integration DFT grid, and the integral accuracy threshold enhanced by 2 orders of magnitude. The frequencies of computed anharmonic modes were not scaled.

In addition to the B97-1 method^83^ and def2-TZVP basis set,^84^ several other model chemistries were used in this study: B3LYP,^92−94^ O3LYP,^93−95^ TPSSh,^96,97^ and B2PLYP^98^ methods combined with the aug-cc-pVTZ,^99^ 6-311++G(3df,3pd),^100,101^ and 6-311++G(d,p)^100,101^ basis sets. We chose these model chemistries because they proved to be the best choices in our previous studies.^102,103^ All possible combinations of the above-mentioned five methods and four basis sets (20 in total) were used for benchmarking computations. Benchmarking data are organized as a collection of frames in six spectral ranges and are included in the Supporting Information (Figure S10). By comparing results of these computations with the experiment, we found that the B97-1/def2-TZVP and B3LYP/6-311++G(d,p) model chemistries, from the viewpoint of the present study, both perform reliably and are comparable (see Figure S2 for the comparison of the scaling procedure). We selected the B97-1/def2-TZVP method to implement throughout this work.

The electronic structures of the radicals generated in the course of photochemistry of BzIm (closed-ring benzimidazolyl and 2-isocyanoanilinyl radicals) were characterized in detail by the analyses of their natural bond orbitals (NBOs) using the NBO 6.0 software package.^104^ Vertical excitation energies of the low-energy electronic excited states were computed using TD-DFT.^105^ The results of these calculations are provided in Table S5 of the Supporting Information. The theoretical UV spectra (Figure S3) were simulated using Lorentzian profiles centered at the computed transition wavelengths with a Lorentzian function having a half-width at half-maximum (hwhm) of 0.124 eV (1000 cm^–1^), as in our previous studies.^35^

The theoretical normal modes of BzIm were analyzed by carrying out potential energy distribution (PED) analysis. The computed force constants with respect to Cartesian coordinates were transformed into the force constants with respect to internal coordinates, which allowed the PED analysis to be carried out as described elsewhere.^106^ The set of internal coordinates used for BzIm was defined as recommended by Pulay *et al.*(107) and is given in Table S1. The atom numbering of BzIm, used for the definition of the internal coordinates, is shown in Figure S1. Marvin software, version 22.15, from ChemAxon, was used for drawing chemical structures, substructures, and reactions.^108^

The PESs along the rotamerization and tautomerization reaction paths were computed at the B97-1/def2-TZVP level by following the intrinsic reaction coordinate (IRC) in both directions. For tunneling computations, we used the potential energy profiles along the IRC, computed in non-mass-weighted Cartesian coordinates expressed in units of Bohr. The transmission coefficients of the H-atom tunneling through a parabolic barrier^109^ were estimated using the Wentzel–Kramers–Brillouin approximation.^110−112^

## Data Availability

Data Availability: The data underlying this study are available in the published article and its Supporting Information.

## Supporting Information Available

Least-squares linear fits of IR spectra computed at the B97-1/def2-TZVP and B3LYP/6-311++G(d,p) levels for 1*H*-tautomer *versus* experimental IR spectrum of BzIm isolated in an Ar matrix at 15 K; normal mode analysis and vibrational assignments of 1*H*-BzIm, 4*H*-BzIm, and 6*H*-BzIm tautomers; vertical excitation energies, oscillator strengths and simulated UV spectra of 1*H*-, 4*H*-, and 6*H*- tautomers obtained in TD-DFT calculations; experimental and computed IR spectra showing isomerization of 6*H*- into 1*H*- and 4*H*-tautomers upon UV irradiation at 283 nm; predominant resonance structures of the 1*H*-benzimidazole, benzimidazolyl radical, and 2-isocyanoanilinyl radical resulting from NRT analysis; computed IRC profiles for the ring-closure reaction of imino-nitrile-ylide to 1*H*-benzimidazole; experimental spectra of UV-irradiated benzimidazole in the 2180–1930 cm^–1^ range and computed IR spectra of putative photoproducts; computed energies of the isomers on the minor ring-opening pathway of benzimidazole, leading from nitrile-ylide, *via* spiro-azirine and triplet vinyl-nitrene, to ketenimine; natural bond orders and natural spin densities computed for anti- and syn-isomers of the 2-isocyanoanilinyl radical; IRC profiles for the H-shifts between neighboring heavy atoms in the 2-isocyanoanilinyl radical; reaction rates for H-atom tunneling in the closed-ring tautomers of BzIm; Cartesian coordinates and energies computed for all isomers of benzimidazole discussed in this work; comparison of computed IR spectra of 1*H*-BzIm (20 model chemistries: 5 methods × 4 basis sets) with the experimental IR spectrum of benzimidazole monomers isolated in an Ar matrix, shown in six spectral ranges; and selected bibliography reporting on multiple medicinal and pharmaceutical applications of benzimidazole and its vibrational characterization (complementary to the collection of references in the main text) (PDF)
