# Supplementary material for: Dual Photochemistry of Benzimidazole
Source: J Org Chem. 2023 Feb 16;88(5):2884–97. doi: 10.1021/acs.joc.2c02560 (PMC9990075; doi:10.1021/acs.joc.2c02560)
Supplement: Supplementary file 1 — jo2c02560_si_001.pdf [file jo2c02560_si_001.pdf]

Supporting Information

Dual Photochemistry of Benzimidazole

José P. L. Roque,[a] Mário T. S. Rosado,[a] Rui Fausto,[a] Igor Reva [a,b,*]

[a] *University of Coimbra, CQC-IMS, Department of Chemistry, 3004-535 Coimbra, Portugal*
[b] *University of Coimbra, CIEPQPF, Department of Chemical Engineering, 3030-790 Coimbra, Portugal*

* Corresponding author e-mail: reva@eq.uc.pt

Table of Contents

		Page
**Bibliography**	Supporting bibliography on multiple medicinal and pharmaceutical applications of benzimidazole (BzIm), and overview of the publications dedicated to structural and spectroscopic characterization of BzIm	**S2**
**Figure S1.**	Geometry and atom numbering scheme of 1*H*-benzimidazole	**S4**
**Figure S2.**	Least squares linear fits of vibrational spectrum of 1*H*-benzimidazole	**S4**
**Table S1.**	Definition of internal coordinates used in the normal mode analysis	**S5**
**Table S2.**	Vibrational assignment of 1*H*-BzIm isolated in an Ar matrix at 15 K	**S6**
**Table S3.**	Vibrational assignment of 4*H*-BzIm isolated in an Ar matrix at 15 K	**S7**
**Table S4.**	Vibrational assignment of 6*H*-BzIm isolated in an Ar matrix at 15 K	**S8**
**Figure S3.**	Simulated UV spectra of 1*H*-BzIm, 4*H*-BzIm, and 6*H*-BzIm	**S9**
**Table S5.**	Computed vertical excitation energies of 1*H*-, 4*H*-, and 6*H*- Isomers	**S9**
**Figure S4.**	Evidence of reversible photoisomerization of 6*H*- into 4*H*- and 1*H*-forms	**S10**
**Figure S5.**	Most significant resonance structures computed for 1*H*-benzimidazole, benzimidazolyl radical, and 2-isocyanoanilinyl radical	**S11**
**Note**	Note on a minor photochannel	**S12**
**Scheme S1.**	Minor photoreaction pathway of 1*H*-BzIm leading to ketenimine	**S12**
**Figure S6.**	IRC profiles computed for the ring-closure reaction of *anti*- and *syn*-isomers of Imino-Nitrile-Ylide to 1*H*-benzimidazole	**S13**
**Figure S7.**	An expanded version of Figure 5 of the main text	**S14**
**Figure S8.**	Bond orders, natural spin densities, and spin isodensity surfaces computed for the *anti*- and *syn*- isomers of 2-isocyanoanilinyl radical	**S15**
**Table S6.**	Computed potential energy barriers for H-atom scrambling around the closed-ring benzimidazole and reaction rates for H-atom tunneling	**S16**
**Table S7.**	Structures and relative energies computed for the open-ring isocyanoaniline and its open-ring prototropic tautomers	**S16**
**Table S8.**	Electronic, Zero-Point Corrected, and Gibbs Free Energies Computed for the Isomers Discussed in This Work	**S17**
**Table S9.**	Cartesian coordinates of selected isomers and photoproducts of BzIm, optimized at the B97-1/def2-TZVP level of theory	**S18**
**Figure S9.**	Comparison of IR spectra of 1*H*-BzIm computed at 20 theory levels with the experimental IR spectrum of matrix-isolated benzimidazole	**S26**
**References.**	Supporting Information References	**S32**

Selected supporting bibliography reporting on multiple medicinal and pharmaceutical applications of benzimidazole (BzIm) and of its derivatives, as well as overview of the publications dedicated to structural and spectroscopic characterization of BzIm in condensed phases and for the monomeric compound (the numbering is independent of the references in the main text).

BzIm nucleus is present in numerous categories such as anticancer,[1-3] antifungal,[4-6] anthelminthic,[4] antiparasitic,[7] anti-inflammatory,[8-9] antibacterial,[10] antimicrobial[11] agents, and is also found in antihypertensives,[12] anticoagulants,[13-14] antidiabetics,[14-15] to list a few. BzIm scaffolds are frequently used in development of new therapeutic agents against multiple diseases[16] having a wide spectrum of biological and pharmacological activities, such as immuno-, hormone, and lipid level modulators, and constitute a subject of multiple reviews.[17-26] BzIm derivatives are also known as inhibitors of different viruses,[27-28] including Zika,[29] Ebola,[30] herpes,[31] hepatitis B.[32] A promising antiviral scaffold[33] of BzIm is comprised in skeleton of drugs targeting picornavirus,[34] arenavirus,[35-36] and also SARS-CoV-2 coronavirus.[37-38] BzIm based compounds were also reported as organic corrosion inhibitors[39-44] for different metals in different media.

Benzimidazole can serve as a multifunctional unit in heteroaromatic molecular systems for optoelectronics, nonlinear optics, photovoltaics, sensing and bioimaging. A review on developments in optical sensing molecular systems that incorporate BzIm structural unit has been recently published by Horak et al.[45] Typical synthetic methods established for BzIm scaffolds are periodically reviewed.[46-51] Among others, photochemical[50] and photocatalytic[51] synthetic methods have been discussed. In the decade of 2000-ies, Vijayan et al. published a series of works where they have prepared and studied BzIm single crystals using a variety of growth and characterization techniques (NMR, FTIR, Raman, UV-Vis, X-ray diffraction, and more).[52-58] The BzIm therein was established as an organic nonlinear optical material whose nonlinear optical harmonic generation efficiency is 4.5 times higher than that of standard potassium dihydrogen phosphate (KDP) single crystal.[59]

Multiple reports address the structure and spectroscopic characterization of BzIm in different *condensed phases*. Early reports of this kind go as far back as the 1960-ies. UV spectra of BzIm in hexane solution were reported in 1961 by Derkosch et al.[60] and, in 1963, in ethanol and cyclohexane by Schütt and Zimmermann.[61] Infrared (IR) and Raman spectra of BzIm in KBr disks and nujol mulls were reported by Morgan in 1961,[62] and also by Cordes and Walter in 1968.[63] The crystal structure of BzIm was first determined by Escande and Galigné in 1974.[64] Then followed several crystallographic studies on BzIm wherein several polymorphs were detected, their lattice parameters were identified, and the crystals were characterized using several spectroscopic techniques.[65-67] FTIR and laser Raman spectra of BzIm in KBr disks were reported: in 1991 by Mohan et al.,[68] in 2002 by Morsy et al.,[69] as well as in 2007 by Sundaraganesan et al.[70] In 2008, Tomkinson reported spectroscopic vibrational data on benzimidazole and benzimidazole-d_2, using inelastic neutron

scattering spectroscopy, revealing some previously unobserved bands, or suggesting new assignments.[71] In 2014, Nieto et al.[72] and in 2018, Larina[73] reported on the tautomerism and structure of azoles, including BzIm, by employing multinuclear dynamic NMR spectroscopy in solutions and in the solid state, showing that prototropic transformations of almost all azoles in solution proceed very quickly, whereas in the solid state they are restrained.

Spectroscopic studies of *monomeric* BzIm were also a subject of several reports. IR and Raman spectra of BzIm in an argon matrix were reported in 1997 by Klots et al.[74] A year later, IR spectra of matrix-isolated BzIm were reported by Schoone et al.[75] In 1991, Caminati et al.,[76] studied the absorption spectra of the 278-nm electronic band system of BzIm and provided assignment of the S_1-S_0 transition by the rotational contour analysis of the 0-0 band. In 1992, Caminati et al. reported the microwave spectra of BzIm, proving the full planarity of the molecule in the gas-phase.[77] In the 1970-ies, Gordon et al. reported the vapor absorption UV spectra of BzIm, with electronic origin of the fully protonated species at 36023 cm^{-1} (277.60 nm), and also studied how it depends on deuteration at different positions.[78-79] In 1993, Jalviste and Treshchalov published laser-induced fluorescence excitation and dispersed fluorescence spectra of jet-cooled BzIm, and detected the origin of the first excited singlet state at 36032 cm^{-1} (277.53 nm).[80] In 1995, Berden et al. reported a more precise value, at 36021.34 cm^{-1} (277.61 nm).[81] In 2006, Schmitt et al. studied the rotationally resolved electronic spectra of four H/D isotopomers and found the electronic origin of the perhydrogenated form of BzIm at nearly the same position, 36021.38 cm^{-1} (277.61 nm).[82] In 2007, Lin et al. reported one-color resonant two-photon ionization spectrum of BzIm, in the energy range near its $S_1 \leftarrow S_0$ transition, with the band origin at 36022 ± 2 cm^{-1}.[83] In 2013-2014, vibronic bands of BzIm between 0,0 and 0,0+1265 cm^{-1} were reported by Schmitt et al.[84-85] There is also a variety of publications dedicated to computational spectra of BzIm. Geometry optimizations of BzIm in electronic excited states, to obtain band origins, relaxed emission energies, oscillator strengths and transition moment directions, were performed by Serrano-Andrés and Borin,[86-87] Arulmozhiraja and Coote,[88] and Yang et al.[89]

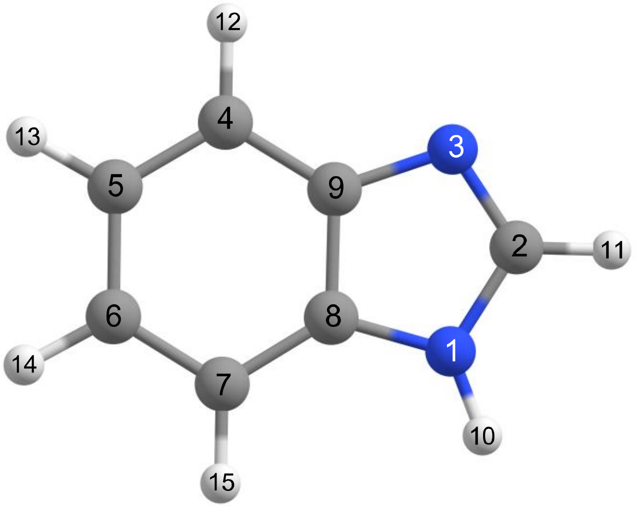

Figure S1. Geometry and atom numbering scheme of 1*H*-benzimidazole used for the definition of internal coordinates (see Table S1). Color codes: blue – nitrogen, grey – carbon, white – hydrogen.

.

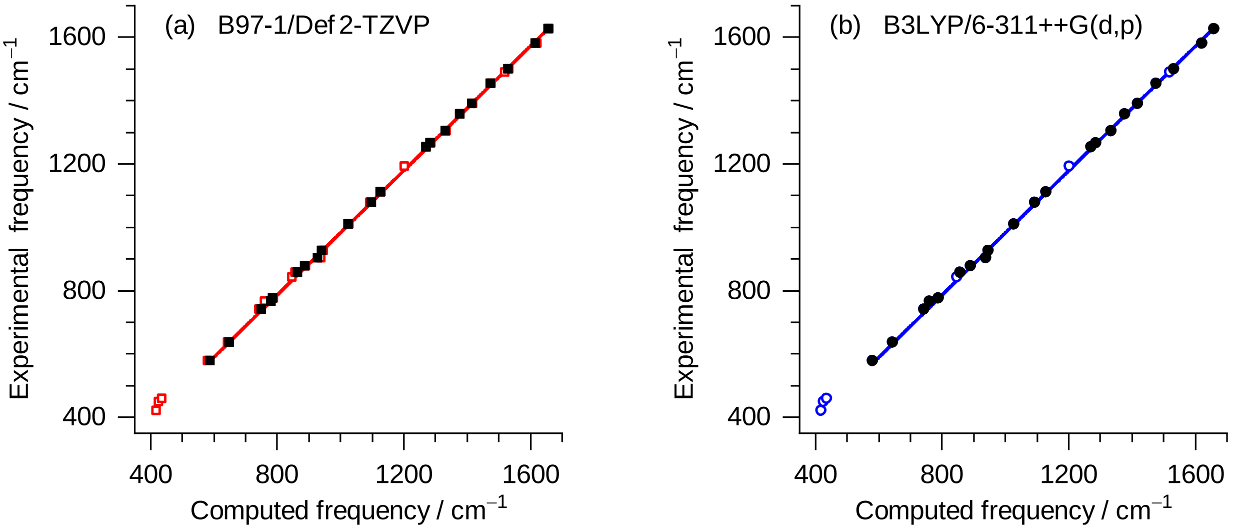

Figure S2. Experimental vibrational frequencies observed in the IR spectrum of benzimidazole monomers isolated in an Ar matrix at 15 K, plotted against theoretical vibrational frequencies of 1*H*-BzIm computed within the harmonic approximation at the (a) B97-1/def2-TZVP level, and (b) B3LYP/6-311++G(d,p) level. The least squares linear fits (a – red line; b – blue line) were carried out using the formula $y=kx+c$, with the intercept set to zero (c=0) in both cases. The following best fit parameters were obtained: (a) k=0.9829, RMSD = 4.1; (b) k=0.9825, RMSD = 8.6. RMSD stands for root-mean-square deviation (cm^{-1}). Twenty-one (21) vibrations from the 1700 – 500 cm^{-1} range were used for the fitting procedure (designated with filled symbols). The open symbols correspond to vibrational modes that either have low predicted IR intensity or are highly anharmonic; these modes were not used in the fits.

Table S1. Definition of internal coordinates used in the normal mode analysis of *1H*-benzimidazole.[a]

Coord.	Definition	Approximate description
	A' symmetry	
S_1	$r_{1,10}$	ν(NH)
S_2	$r_{2,11}$	ν(C2H)
S_3	$2\ (r_{4,12} + r_{5,13} + r_{6,14} + r_{7,15})$	ν_a(CH)
S_4	$2\ (r_{4,12} + r_{5,13} - r_{6,14} - r_{7,15})$	ν_b(CH)
S_5	$2\ (r_{4,12} - r_{5,13} - r_{6,14} + r_{7,15})$	ν_c(CH)
S_6	$2\ (r_{4,12} - r_{5,13} + r_{6,14} - r_{7,15})$	ν_d(CH)
S_7	$2\ (r_{4,5} - r_{6,7} + r_{7,8} - r_{9,4})$	ν_a(CC)
S_8	$(12^{-1/2})(-r_{4,5} + 2r_{5,6} - r_{6,7} - r_{7,8} + 2r_{8,9} - r_{9,4})$	ν_b(CC)
S_9	$2\ (r_{4,5} - r_{6,7} - r_{7,8} + r_{9,4})$	ν_c(CC)
S_{10}	$(12^{-1/2})(r_{4,5} + 2r_{5,6} + r_{6,7} - r_{7,8} - 2r_{8,9} - r_{9,4})$	ν_d(CC)
S_{11}	$(6^{-1/2})(r_{4,5} - r_{5,6} + r_{6,7} - r_{7,8} + r_{8,9} - r_{9,4})$	ν_e(CC)
S_{12}	$(6^{-1/2})(r_{4,5} + r_{5,6} + r_{6,7} + r_{7,8} + r_{8,9} + r_{9,4})$	ν_f(CC)
S_{13}	$r_{2,3}$	ν(C=N)
S_{14}	$r_{1,2}$	ν(N1C2)
S_{15}	$(2^{-1/2})(r_{3,9} + r_{1,8})$	ν_s(C–N)
S_{16}	$(2^{-1/2})(r_{3,9} - r_{1,8})$	ν_{as}(C–N)
S_{17}	$(2^{-1/2})(\beta_{8,10,1} - \beta_{2,10,1})$	δ(NH)
S_{18}	$(2^{-1/2})(\beta_{1,11,2} - \beta_{3,11,2})$	δ(C2H)
S_{19}	$2\ (\beta_{8,15,7} - \beta_{6,15,7} - \beta_{9,12,4} + \beta_{5,12,4})$	δ_a(CH)
S_{20}	$2\ (\beta_{8,15,7} - \beta_{6,15,7} + \beta_{9,12,4} - \beta_{5,12,4})$	δ_b(CH)
S_{21}	$2\ (\beta_{7,14,6} - \beta_{5,14,6} - \beta_{6,13,5} + \beta_{4,13,5})$	δ_c(CH)
S_{22}	$2\ (\beta_{7,14,6} - \beta_{5,14,6} + \beta_{6,13,5} - \beta_{4,13,5})$	δ_d(CH)
S_{23}	$(6^{-1/2})(\beta_{4,6,5} - \beta_{5,7,6} + \beta_{6,8,7} - \beta_{7,9,8} + \beta_{8,4,9} - \beta_{9,5,4})$	δ_a(Bz)
S_{24}	$(12^{-1/2})(-\beta_{4,6,5} - \beta_{5,7,6} + 2\beta_{6,8,7} - \beta_{7,9,8} - \beta_{8,4,9} + 2\beta_{9,5,4})$	δ_b(Bz)
S_{25}	$2\ (\beta_{4,6,5} - \beta_{5,7,6} + \beta_{7,9,8} - \beta_{8,4,9})$	δ_c(Bz)
S_{26}	$((1+2a^2+2b^2)^{-1/2})(\beta_{1,3,2} + a(\beta_{2,9,3} + \beta_{8,2,1}) + b(\beta_{3,8,9} + \beta_{9,1,8}))$	δ_a(Im)
S_{27}	$(2(a-b)^2 + 2(1-a)^2)^{-1/2})((a-b)(\beta_{2,9,3} - \beta_{8,2,1}) + (1-a)(\beta_{3,8,9} - \beta_{9,1,8}))$	δ_b(Im)
	A'' symmetry	
S_{28}	$2\ (\gamma_{12,9,4,5} - \gamma_{13,4,5,6} + \gamma_{14,5,6,7} - \gamma_{15,6,7,8})$	γ_a(CH)
S_{29}	$2\ (\gamma_{12,9,4,5} - \gamma_{13,4,5,6} - \gamma_{14,5,6,7} + \gamma_{15,6,7,8})$	γ_b(CH)
S_{30}	$2\ (\gamma_{12,9,4,5} + \gamma_{13,4,5,6} - \gamma_{14,5,6,7} - \gamma_{15,6,7,8})$	γ_c(CH)
S_{31}	$2\ (\gamma_{12,9,4,5} + \gamma_{13,4,5,6} + \gamma_{14,5,6,7} + \gamma_{15,6,7,8})$	γ_d(CH)
S_{32}	$\gamma_{11,1,2,3}$	γ(C2H)
S_{33}	$\gamma_{10,8,1,2}$	γ(NH)
S_{34}	$(6^{-1/2})(\tau_{4,5,6,7} - \tau_{5,6,7,8} + \tau_{6,7,8,9} - \tau_{7,8,9,4} + \tau_{8,9,4,5} - \tau_{9,4,5,6})$	τ_a(Bz)
S_{35}	$(12^{-1/2})(2\tau_{4,5,6,7} - \tau_{5,6,7,8} - \tau_{6,7,8,9} + 2\tau_{7,8,9,4} - \tau_{8,9,4,5} - \tau_{9,4,5,6})$	τ_b(Bz)
S_{36}	$2\ (\tau_{5,6,7,8} - \tau_{6,7,8,9} + \tau_{8,9,4,5} - \tau_{9,4,5,6})$	τ_c(Bz)
S_{37}	$((1+2a^2 + 2b^2)^{-1/2})(\tau_{1,8,9,3} + b(\tau_{3,2,1,8} + \tau_{9,3,2,1}) + a(\tau_{2,1,8,9} + \tau_{8,9,3,2}))$	τ_a(Im)
S_{38}	$(2(a-b)^2 + 2(1-a)^2)^{-1/2})((a-b)(\tau_{8,9,3,2} - \tau_{2,1,8,9}) + (1-a)(\tau_{9,3,2,1} - \tau_{3,2,1,8}))$	τ_b(Im)
S_{39}	$(2^{-1/2})(\tau_{1,8,9,4} - \tau_{7,8,9,3})$	γ(Bz–Im)

[a] See Figure S1 for the atom numbering scheme and Table S2 for the results of the normal mode analysis; $r_{i,j}$ is the distance between atoms A_i and A_j; $\beta_{i,j,k}$ is the angle between vectors A_kA_i and A_kA_j; $\tau_{i,j,k,l}$ is the dihedral angle between the plane defined by A_i, A_j, A_k and the plane defined by A_j, A_k and A_l atoms; $\gamma_{i,j,k,l}$ is the angle between the vector A_kA_i and the plane defined by atoms A_j, A_k, A_l. The combinations [(+), (+)] and [(+), (−)] denote in-phase and in-opposite-phase couplings between coordinates of different types. Coefficients: a = cos 144° = −0.809; b = cos 72° = 0.309. Abbreviations: ν = stretching, δ = in-plane bending, γ = out-of-plane bending, τ = torsion, Bz = six-membered (benzene) ring, Im = five-membered (imidazole) ring.

Table S2. Experimental wavenumbers and infrared intensities of BzIm isolated in an Ar matrix at 15 K, compared to the harmonic and anharmonic wavenumbers ($\tilde{\nu}$ / cm^{-1}) and absolute infrared intensities (A^{th} / km mol^{-1}) of 1*H*-BzIm, computed at the B3LYP/def2-TZVP level.

Experimental [a]		Harmonic [b]		Anharmonic [b]		Sym.	Assignment [c] (PED [d])
ν	I	$\tilde{\nu}$	A^{th}	$\tilde{\nu}$	A^{th}		
3508.7/3502.1	127.6	3499.9	64.1	3500.0	49.8	A'	ν(NH) (99.7)
3093.3	2.1	3083.8	1.3	3106.3	0.7	A'	ν(C2H) (99.3)
3076.5/3074.0	7.7	3052.4	8.5	3074.5	3.1	A'	ν_a(CH) (79.1) ; ν_b(CH) (15.5)
3065	vw	3044.1	15.3	3059.9	1.3	A'	ν_b(CH) (56.6) ; ν_a(CH) (17.4) ; ν_c(CH) (19.0)
3051.9	6.4	3032.9	8.8	3040.7	14.6	A'	ν_c(CH) (75.5) ; ν_b(CH) (21.5)
-		3023.7	0.02	3012.9	0.3	A'	ν_d(CH) (87.9)
1626.7	5.5	1629.4	6.9	1615.6	5.1	A'	ν_a(CC) (54.9)
1582.8/1581.2	2.0	1591.1	3.6	1582.6	2.2	A'	ν_b(CC) (63.8)
1504.2/1499.5	22.3	1505.1	27.1	1499.8	21.4	A'	[ν(C=N) (48.5) − δ(C2H) (15.3)]
1494.1/1489.1	3.2	1492.5	3.5	1488.5	1.7	A'	[ν_c(CC) (32.3) − δ_d(CH) (30.2)]
1454.5	26.6	1450.6	21.5	1444.0	18.6	A'	[δ_b(CH) (38.0) − ν_d(CC) (29.5)]
1391.2	21.7	1394.4	29.2	1383.0	16.9	A'	δ(NH) (28.4) ; δ_d(CH) (14.8) ; ν_e(CC) (14.6)
1357.7	20.5	1355.3	27.2	1349.4	19.7	A'	ν_e(CC) (41.5)
1304.1	8.4	1310.7	9.6	1306.9	8.0	A'	[ν_{as}(C−N) (19.0) − δ(C2H) (16.2) − ν(C=N) (15.9)];
1265.3	26.9	1264.0	30.5	1258.8	27.8	A'	[ν_s(C−N) (25.6) − δ_a(CH) (17.9)]
1252.7	15.1	1250.9	16.3	1248.1	12.2	A'	[ν_s(C−N) (19.0) + δ_a(CH) (17.6)]
1178.3	0.4	1183.1	1.4	1177.9	0.8	A'	[ν_{as}(C−N) (22.9) + δ(C2H) (21.0) − δ_a(Bz) (18.6)];
1147.8	0.6	1146.9	1.5	1151.1	0.9	A'	δ_c(CH) (51.8) ; δ_b(CH) (23.9)
1112.0	1.5	1109.3	1.9	1112.2	1.7	A'	[ν_c(CC) (22.7) + δ_d(CH) (21.7)]; δ_a(CH)(20.2) ; δ_a(Bz)(14.9)
1079.3	12.8	1080.4	17.5	1072.1	8.1	A'	ν(N1C2) (55.2) ; δ(NH) (29.5)
1008.9	5.5	1007.3	5.5	1007.4	4.7	A'	[ν_d(CC) (44.1) + δ_b(CH) (22.4)]; ν_f(CC) (26.7)
944.0/942.6	9.8			972.0	36.4	A'	2 × (γNH)
927.0/925.8	3.0	926.4	1.4	934.0	5.8	A'	δ_a(Im) (69.4)
878.4/876.7	5.2	873.1	3.1	879.2	2.2	A'	[δ_a(Bz) (51.5) + ν_{as}(C−N) (21.9)]
776.6/775.9	5.2	774.5	4.4	775.5	2.8	A'	ν_f(CC) (40.6) ; ν_s(C−N) (18.1) ; δ_b(Bz) (15.6)
-		615.4	0.1	617.9	0.3	A'	δ_b(Im) (46.6) ; δ_c(Bz) (29.1)
-		540.0	0.2	543.5	0.2	A'	δ_b(Bz) (70.9)
421.0	14.0	410.3	7.8	412.8	7.6	A'	δ_c(Bz) (53.2)
-		941.3	0.3	1143.9	0.5	A"	[γ_a(CH) (100.7) − γ_b(CH) (18.2)]
902.0	5.0	909.3	2.6	1002.7	4.5	A"	[γ_b(CH) (78.9) + γ_a(CH) (14.6)]
860.7/857.6	12.0	851.0	10.8	883.4	1.9	A"	[γ(C2H) (70.9) − γ_c(CH) (15.2)]
841.6	0.8	836.9	1.0	838.7	7.7	A"	[γ_c(CH) (54.8) + γ(C2H) (30.9)]
768.8/766.0	7.4	768.2	5.3	806.7	5.0	A"	[τ_a(Bz) (46.5) − τ_a(Im) (30.9)] ; γ_c(CH) (17.3)
742.2/740.3	91.9	737.0	71.6	749.9	74.0	A"	γ_d(CH) (84.1)
636.7	1.7	638.2	2.4	648.1	0.8	A"	τ_b(Im) (88.5)
579.3/577.8	4.5	577.0	3.9	613.4	2.4	A"	[τ_a(Bz) (47.1) + τ_a(Im) (25.7) − τ_b(Bz) (39.0)]
459.5 [e]	27.5	421.6	0.3	425.0	13.4	A"	[τ_c(Bz) (82.6) − γ(Bz–Im) (18.6)]
449.8 [e]	40.7	417.0	93.0	478.7	73.3	A"	γ(NH) (93.6)
-		250.0	3.2	254.1	4.8	A"	[τ_b(Bz) (61.2) + τ_a(Im) (33.9)]
-		217.7	8.6	222.5	7.6	A"	[γ(Bz–Im) (67.8) + τ_c(Bz) (22.9)]

[a] For doublet bands, the strongest component is underlined. Experimental intensities (I) were normalized in such a way that the sum of integrated infrared (IR) intensities of the fundamental modes in the 1700-500 cm^{-1} range was equal to the of sum computed harmonic IR intensities in the same range. [b] Computed harmonic wavenumbers were multiplied by 0.955 (above 3000 cm^{-1}) or 0.983 (below 3000 cm^{-1}). The scaling factor of 0.983 obtained from least-squares linear fit of the harmonic wavenumbers computed for 1*H*-BzIm against the experimental wavenumbers of **1** in an Ar matrix in the 1700–500 cm^{-1} range (See Fig. S2). Computed anharmonic wavenumbers were not scaled. [c] Abbreviations: ν = stretching, δ = in-plane bending, γ = out-of-plane bending, τ = torsion, Bz = six-membered (benzene) ring, Im = five-membered (imidazole) ring, vw = very weak, PED = potential energy distribution. The symbols (+) and (−) denote in-phase and in-opposite-phase couplings between the contributing internal coordinates. [d] See Table S1 for the definition of internal coordinates. Only contributions with PED over 15% are included. [e] Strongly anharmonic and mixed, tentative assignment is based on IR intensities.

Table S3. Vibrational assignment of the experimental infrared bands of photoproduct **A** (identified as 4*H*-BzIm), trapped in an Ar matrix at 15 K, and their comparison to the harmonic wavenumbers ($\tilde{\nu}$ / cm^{-1}) and absolute infrared intensities (A^{th} / km mol^{-1}) of 4*H*-BzIm computed at the B97-1/def2-TZVP level of theory.

Ar matrix [a]		Calc.[b]		Sym.	Approx. Assignment [c]
ν	I	$\tilde{\nu}$	A^{th}		
1657	m	1662.8	21.1	A'	ν(C7=C8) − ν(C5=C6)
1560	s	1570.3	84.1	A'	ν(C7=C8) + ν(C5=C6)
1556	sh	1561.2	8.6	A'	ν(N3=C9)
1426	m	1427.0	45.3	A'	ν(C2=N1)
1409	m	1407.3	14.0	A'	δ(C5H + C6H)
1384	s	1372.0	117.9	A'	ν(C8−C9)
1360	m	1360.3	41.0	A'	δ(CH_2)
1287	m	1294.7	24.0	A'	δ(C2H)
1279	w	1288.9	30.1	A'	ν(C5−C4−C9)$_{as}$
		1247.4	7.6	A'	ω(CH_2) + δ(C7H)
		1171.2	4.2	A'	δ(C5H − C6H)
1143	w	1144.9	11.1	A'	ω(CH_2) + δ(CH)
		1141.4	2.7	A"	tw(CH_2)
1054	s	1068.7	90.2	A'	ν(N3−C2)
1011	s	1014.9	24.0	A'	δ(C7H – C6H – C5H)
		969.6	0.01	A"	γ(C7H – C6H + C5H)
938		957.4	7.8	A"	γ(C7H – C5H)
		928.7	2.1	A"	γ(C2H)
932	m	926.9	20.0	A'	δ$_a$(Bz)
1*H*-		903.8	5.4	A'	δ(Imi)
		888.5	4.0	A"	ρ(CH_2) + γ(C6H)
869	w	865.3	7.2	A'	δ$_b$(Bz)
		730.9	1.8	A'	δ$_c$(Bz)
710	m	711.9	38.5	A"	γ(C7H + C5H + C6H)
681	w	676.5	15.9	A"	τ(Bz) + τ(Imi)

[a] Experimental intensities (I) are given in qualitative terms: s = strong; m = medium; w = weak, sh = shoulder. [b] Computed harmonic wavenumbers (in cm^{-1}) scaled by 0.983. The scaling factor obtained from least-squares linear fit of the computed wavenumbers against the experimental wavenumbers of BzIm in an Ar matrix in the 1700–500 cm^{-1} range (See Fig. S2). [c] Abbreviations: ν = stretching, δ = in-plane bending, γ = out-of-plane bending, τ = torsion, ω = wagging, ρ = rocking, tw = twisting, as = antisymmetric, Bz = 6-membered (“benzene”) ring, Imi = 5-membered (“imidazole”) ring, (δ$_a$, δ$_b$, δ$_c$) = three orthogonal bending modes of the Bz ring. The expected spectral position of the photoproduct 4*H*-BzIm, which should overlap with nearby strong IR bands of the 1*H*- precursor, is designated as “1*H*-”.

Table S4. Vibrational assignment of the experimental infrared bands of photoproduct **B** (identified as 6*H*-BzIm), trapped in an Ar matrix at 15 K, and their comparison to the harmonic wavenumbers ($\tilde{\nu}$ / cm^{-1}) and absolute infrared intensities (A^{th} / km mol^{-1}) of 6*H*-BzIm computed at the B97-1/def2-TZVP level of theory.

Ar matrix [a]		Calc.[b]		Sym.	Approx. Assignment [c]
ν	I	$\tilde{\nu}$	A^{th}		
1665	m	1672.1	36.2	A'	ν(C7=C8)
1601	m	1608.8	28.4	A'	ν(C5=C4) + ν(C9=N3)
1548	s	1555.4	57.0	A'	ν(C5=C4) – ν(C9=N3) + ν(C2=N1)
1446	w	1447.9	15.7	A'	ν(C2=N1)
1406	s	1393.9	71.8	A'	ν(C8−C9) ?
1367	m	1368.6	43.8	A'	δ(CH_2)
		1361.8	7.7	A'	δ(C5H + C4H)
		1327.1	7.0	A'	ω(CH_2)
1288	m	1298.5	39.2	A'	δ(C2H)
		1246.7	3.1	A'	δ(C7H – C4H)
		1161.7	0.1	A"	tw(CH_2)
1151	w	1151.9	18.1	A'	ν(C8−N1)
1140	m	1134.8	14.4	A'	δ(C5H − C4H)
1052	s	1070.7	90.9	A'	ν(N3−C2)
1018	w	1014.4	6.9	A'	ω(CH_2) + ν(CC)
		985.9	0.5	A"	τ(C5H − C4H)
935	m	951.9	14.6	A"	τ(C2H)
923	m	932.9	15.4	A"	τ(C7H + C4H)
1*H*-		904.9	5.9	A'	δ(Imi)
1*H*-		873.3	14.1	A'	δ_a(Bz)
1*H*-		856.6	11.6	A'	δ_b(Bz)
810	m	821.4	13.7	A"	γ(Bz)
		818.8	3.8	A"	ρ(CH_2) + τ(CH)
		738.4	2.1	A'	δ_c(Bz)
685	w	683.7	23.6	A"	ρ(CH_2)+τ(C5H+C4H)

[a] Experimental intensities (I) are given in qualitative terms: s = strong; m = medium; w = weak. [b] Computed harmonic wavenumbers (in cm^{-1}) scaled by 0.983. The scaling factor obtained from least-squares linear fit of the computed wavenumbers against the experimental wavenumbers of BzIm in an Ar matrix in the 1700–500 cm^{-1} range (See Fig. S2). [c] Abbreviations: ν = stretching, δ = in-plane bending, γ = out-of-plane bending, τ = torsion, ω = wagging, ρ = rocking, tw = twisting, Bz = 6-membered ("benzene") ring, Imi = 5-membered ("imidazole") ring, (δ_a, δ_b, δ_c) = three orthogonal bending modes of the Bz ring. The expected spectral positions of the photoproduct 6*H*-BzIm, which should overlap with nearby strong IR bands of the 1*H*- precursor, are designated as "1*H*-".

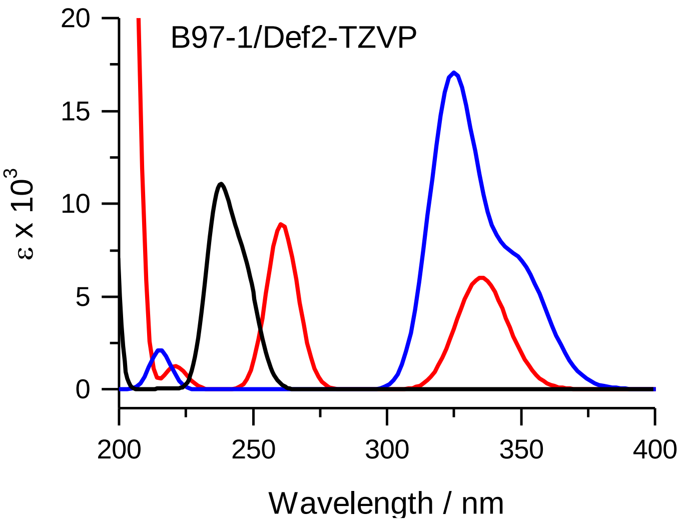

Figure S3. Simulated UV spectra of 1*H*-BzIm (black line), 4*H*-BzIm (blue line), and 6*H*-BzIm (red line) obtained from vertical transitions computed at the B97-1/def2-TZVP level using the time-dependent density functional theory (see Table S5). Each computed transition was convoluted with a Lorentzian function having a half-width at half-height of 0.124 eV (1000 cm^{-1}).

Table S5. Vertical excitation energies (λ_{calc}, nm) and corresponding oscillator strengths (f_{calc}) computed at the B97-1/def2-TZVP level using the time-dependent density functional theory (TD-DFT) for the fifteen lowest-energy excited states of 1*H*-, 4*H*- and 6*H*-BzIm.

	1*H*-			4*H*-			6*H*-	
State	λ_{calc}	f_{calc}	State	λ_{calc}	f_{calc}	State	λ_{calc}	f_{calc}
^{3}A'	345.84	0	^{3}A'	660.21	0	^{3}A'	540.45	0
^{3}A'	300.89	0	^{3}A'	426.73	0	^{3}A'	386.70	0
^{3}A'	261.80	0	^{3}A"	394.50	0	^{3}A"	366.99	0
^{1}A'	247.01	0.0530	^{1}A"	373.77	0.0007	^{1}A"	341.55	0.0011
^{3}A'	245.53	0	^{1}A'	349.38	0.0595	^{1}A'	335.01	0.0545
^{1}A'	237.04	0.0934	^{3}A"	342.28	0	^{3}A"	318.48	0
^{3}A'	235.48	0	^{1}A'	324.59	0.1545	^{3}A'	296.75	0
^{3}A'	229.33	0	^{1}A"	319.20	0.0003	^{1}A"	294.71	0.0000
^{1}A"	216.53	0.0005	^{3}A'	288.24	0	^{1}A'	260.67	0.0820
^{3}A'	208.00	0	^{3}A'	240.26	0	^{3}A'	254.64	0
^{3}A"	197.73	0	^{3}A'	217.42	0	^{3}A"	223.64	0
^{1}A"	195.35	0.0001	^{1}A'	215.30	0.0197	^{1}A'	221.09	0.0114
^{3}A"	194.41	0	^{3}A"	208.15	0	^{3}A'	221.06	0
^{1}A"	193.70	0.0005	^{3}A'	207.93	0	^{1}A"	216.46	0.0002
^{1}A'	193.56	0.3508	^{3}A"	203.05	0	^{3}A'	213.12	0

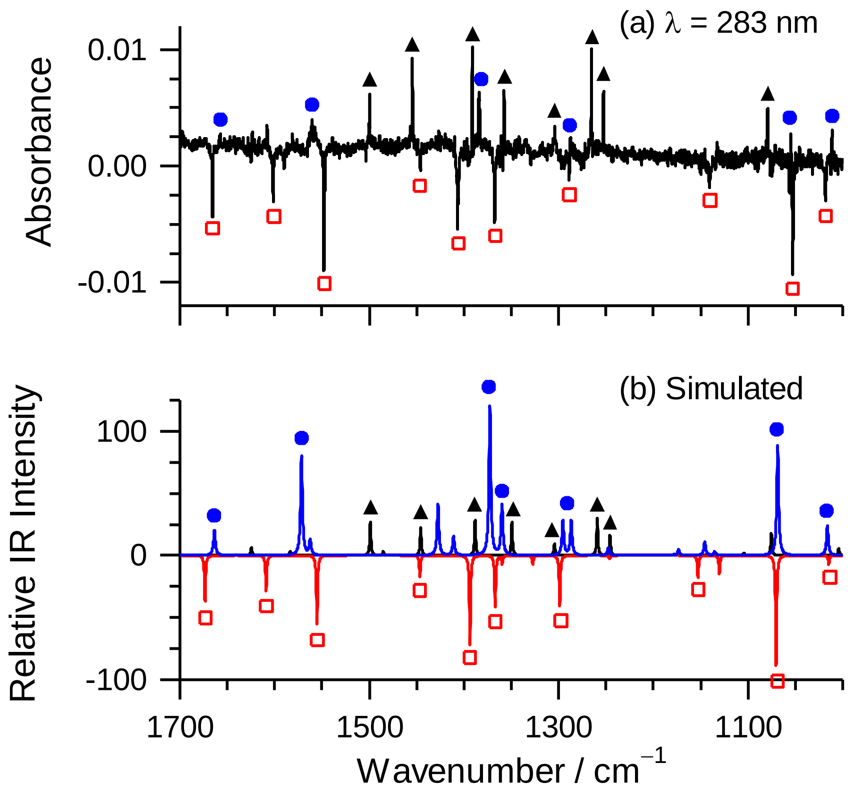

Figure S4. (a) Experimental difference IR spectrum showing changes upon the *third* UV irradiation of the sample at λ = 283 nm, following the initial *(first)* irradiation of matrix-isolated **1** at λ = 260 nm and the subsequent *(second)* irradiation at λ = 330 nm. The downward bands are due to consumed species assigned to 6*H*- (□ red squares) and the upward bands are due to the photoproduced species assigned to 1*H*- (▲ black triangles) and 4*H*-BzIm (● blue circles); (b) Simulated IR spectra of tautomers 1*H*- (black line), 4*H*- (blue line) and 6*H*-BzIm (red line) computed at the B97-1/def2-TZVP level of theory. The theoretical vibrational frequencies were scaled by 0.983, and the IR intensity of 6*H*- was multiplied by (−1).

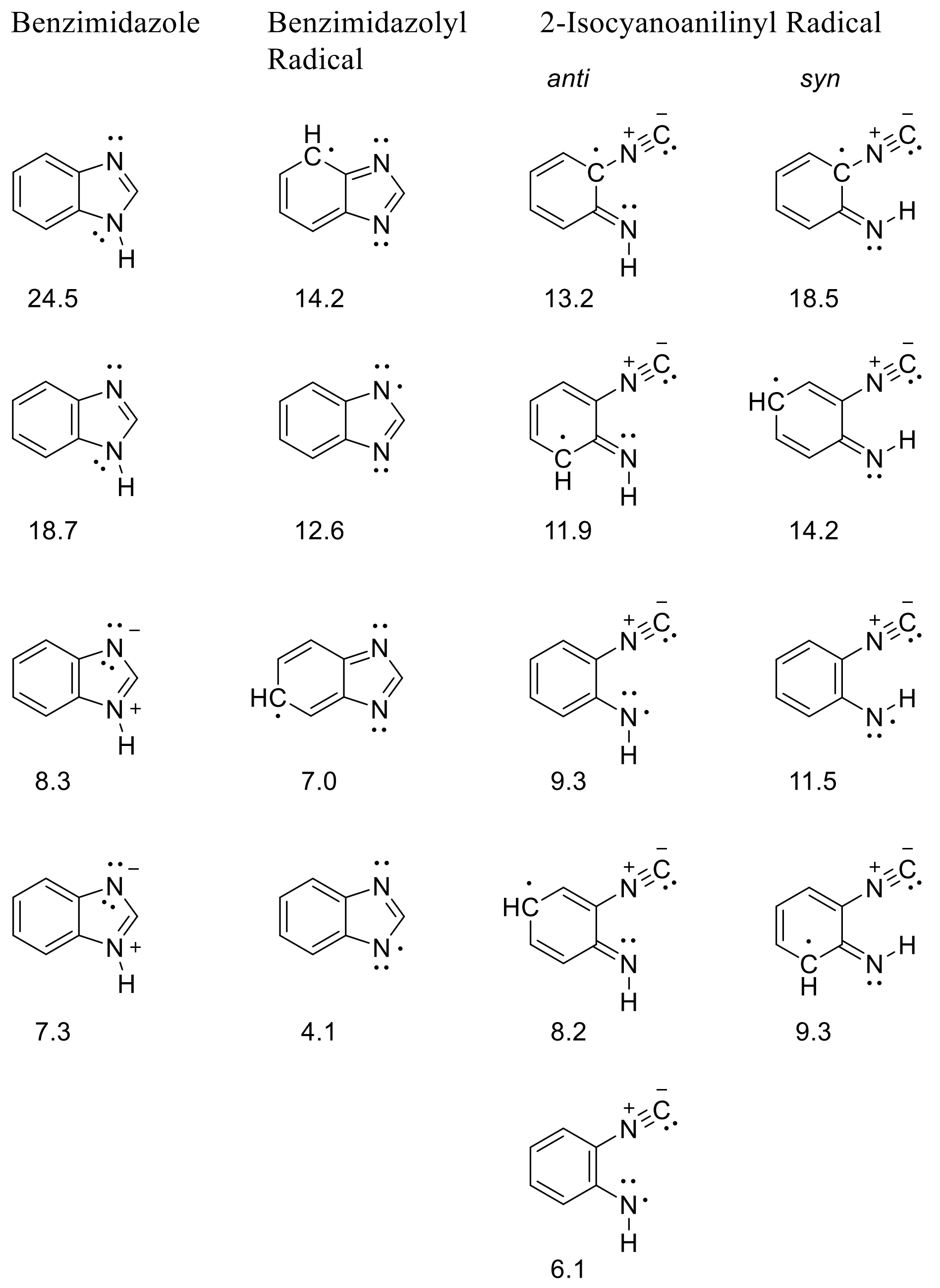

Figure S5. Most significant resonance structures (overall weights greater than 5%) found by the NRT analysis carried out for benzimidazole, benzimidazolyl radical, and 2-isocyanoanilinyl radical (*anti* and *syn*) at the UB97-1/def2-TZVP level. Resonance weights for each structure correspond to the mean value obtained for complementary alpha-spin and beta-spin manifolds. Structures with more than 1 radical center were excluded.

Note on a minor ring-opening photochannel.

Just like in benzoxazole, the sole cleavage of the N1−C2 bond in benzimidazole would lead to a fleeting nitrile ylide species. The potential energy barrier height for the back-reaction from nitrile ylide (*anti*-imino) to benzimidazole, in the electronic ground state, is as low as 3.1 kJ mol^{-1} only (Figure S6a) and this reaction should therefore be extremely fast. A second isomer, *syn*-imino nitrile ylide, is much more stable than the former, the barrier height preventing it from a fast decay back to benzimidazole is 28.6 kJ mol^{-1} (Figure S6b). Such a species would manifest itself by a characteristic very strong IR absorption below 2000 cm^{-1} due to ν_{as}(CNC) mode[80] (predicted values for this mode in *syn*-imino nitrile ylide are 1959 cm^{-1}, 547.6 km mol^{-1}), however no such photoproduct band was detected in this work (see Figure S7). Indirect evidence of the fleeting nitrile-ylide intermediate is the observation of absorptions around 2050-2020 cm^{-1}, a very characteristic spectral manifestation of the ketenimine moiety.[81-84] The (imino)-ketenimine (−C=C=NH) **27** is isomeric of (imino)-nitrile-ylide (−C=N=CH) **24** and may be formed via intermediacy of (imino)-spiro-azirine **25** and triplet (imino)-vinyl-nitrene **26** (see Scheme S1), similarly as it was reported for the respective (oxo)-substituted isomers.[85] The antisymmetric stretching vibration of ketenimine ν_{as}(CCN) has an intrinsically huge infrared intensity (above 800 km mol^{-1}, see Figure S7) and, considering the intensity of its experimental counterpart, the respective isomer should account for no more than 1-2% of the total.

Scheme S1. Computed energies of the isomers of benzimidazole, on the minor ring-opening pathway leading from nitrile-ylide **24**, via *spiro*-azirine **25** and triplet vinyl-nitrene **26**, to ketenimine **27**.

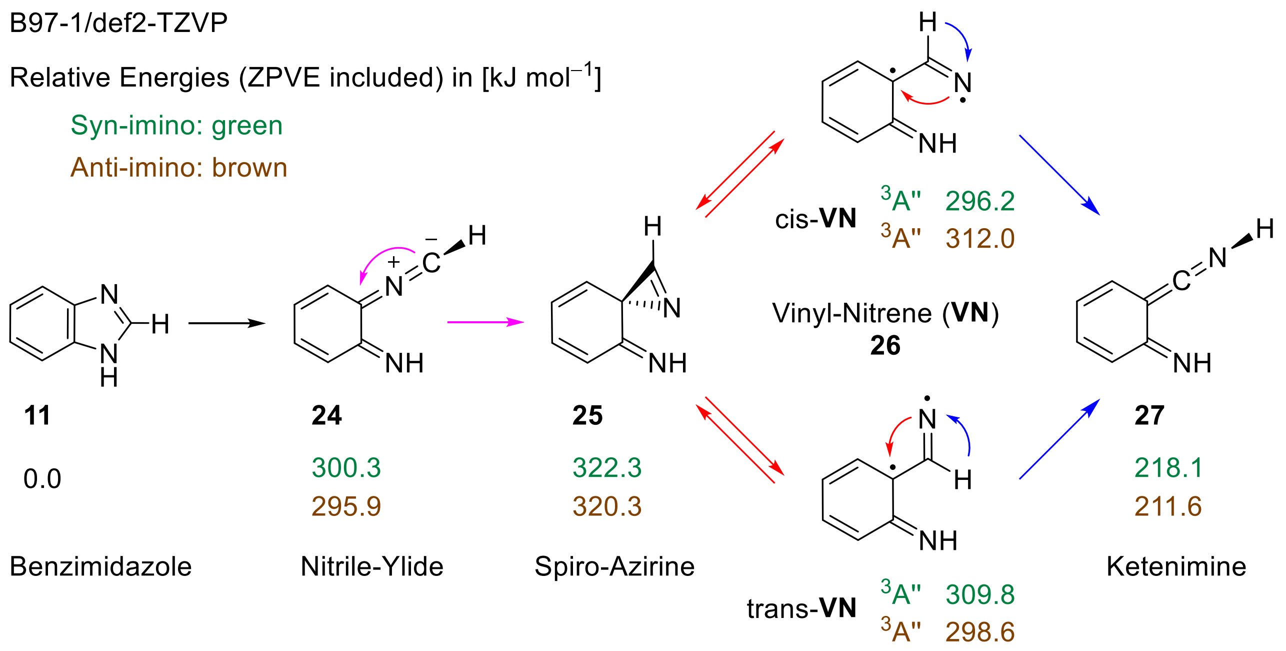

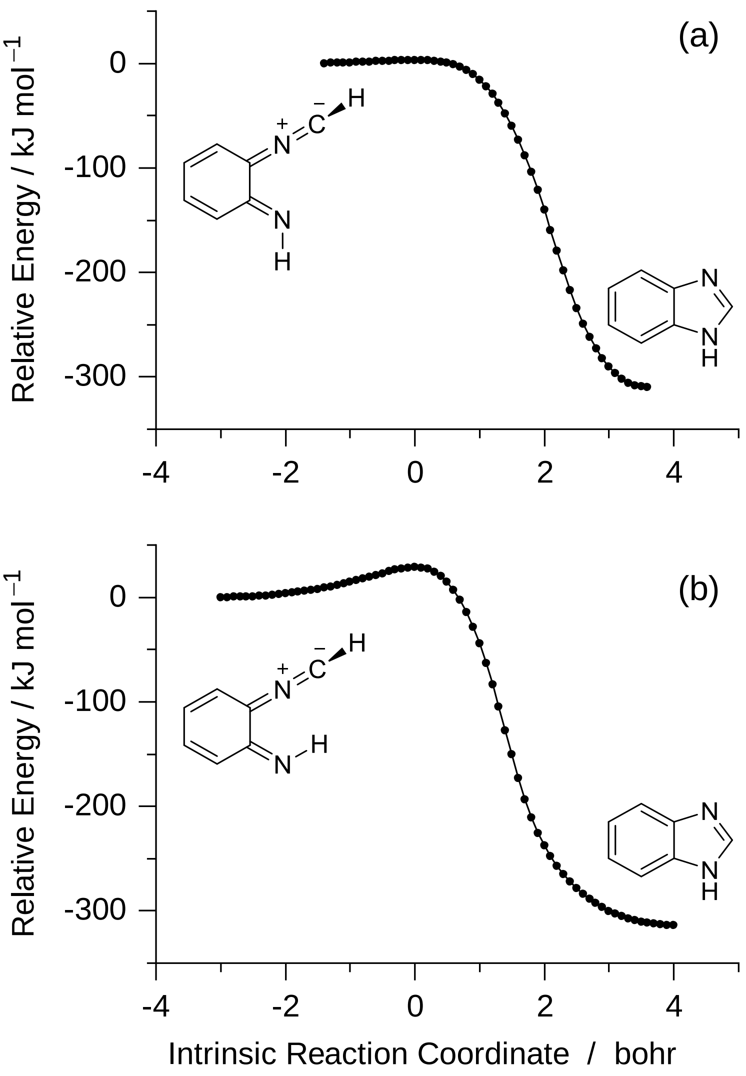

Figure S6. Intrinsic reaction coordinate profiles for the ring-closure reaction of (a) *anti*-Imino-Nitrile-Ylide and (b) *syn*-Imino-Nitrile-Ylide to 1*H*-benzimidazole computed at the B97-1/def2-TZVP level in Cartesian (non-mass-weighted) coordinates.

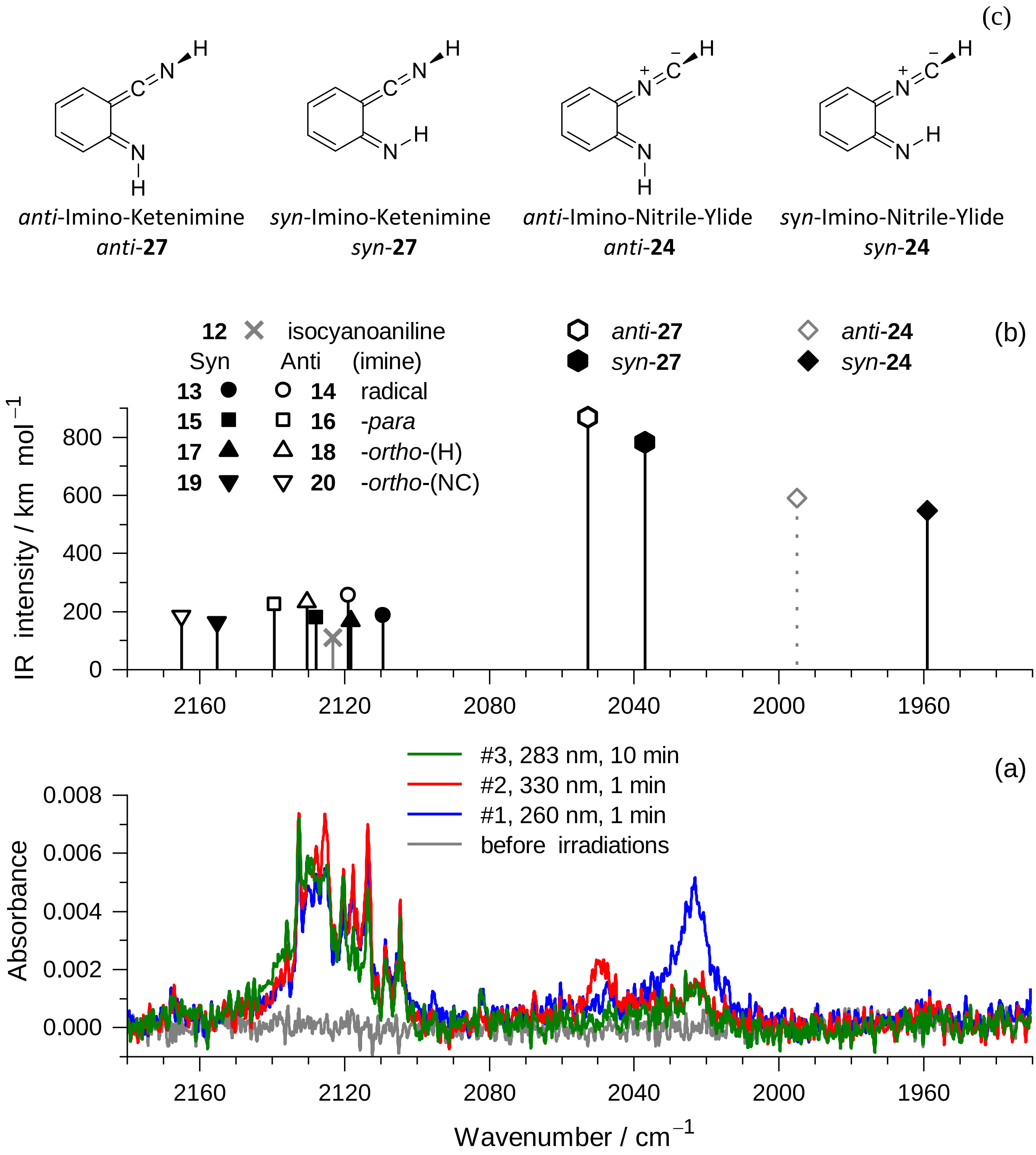

Figure S7. (a) 2180-1930 cm^{-1} range of the infrared spectra of benzimidazole isolated in an Ar matrix at 15 K: before irradiations (gray line) and after UV irradiations at 260 nm (blue line), 330 nm (red line) and 283 nm (green line); (b) B97-1/def2-TZVP computed harmonic wavenumbers (scaled) and IR intensities (not scaled) of putative open-ring photoproducts. The structures of isocyano photoproducts **12-20** are shown in Chart 2 (main text); (c) structures of *anti*-Imino-Ketenimine, *syn*-Imino-Ketenimine, *anti*-Imino-Nitrile-Ylide and *syn*-Imino-Nitrile-Ylide (see also Scheme S1). Note: this figure is an expanded version of Figure 5 of the main text.

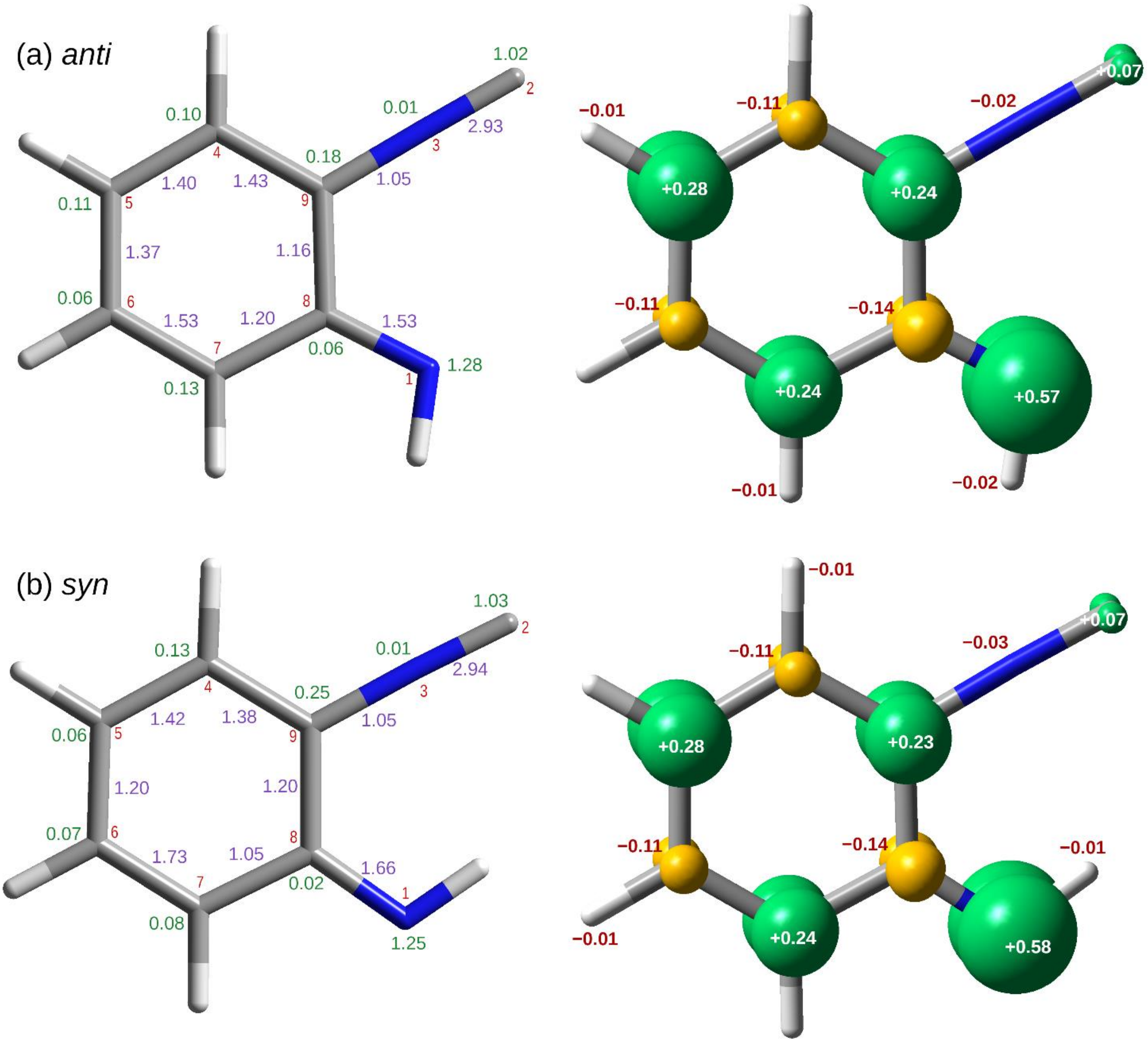

Figure S8. Structures of (a) *anti* and (b) *syn* (with respect to the N1H bond) 2-isocyanoanilinyl radicals optimized at the UB97-1/def2-TZVP level of theory. The adopted numbering of heavy atoms is inherited from the benzimidazole molecule and is shown in red. Left: The bond orders, computed using the Natural Resonance Theory (NRT), are shown in purple (for the bonds between the heavy atoms). The non-bonding orbital populations at the heavy atoms are shown in green. Right: Spin isodensity surface (isovalue ±0.01 e) for the two radical isomers. The values near the heavy atoms correspond to the computed atomic natural spin density values. Blue color stands for the α spin density ("+") and yellow for the β spin density ("–"). Element colors: H – white; C – grey; N – blue.

Table S6. Parameters of the potential energy barriers along the intrinsic reaction coordinate for the isomerization reactions depicted in Figure 7, and reaction rates for H-atom tunneling from the vibrational ground state.

Reaction	Barrier [b]		Rate Constant [c]	
	width (bohr)	height ($kJ\ mol^{-1}$)	k (s^{-1})	$t_{1/2}$ (s)
2H → 1H	2.48	90.94	1.34×10^{-5}	5.17×10^{4}
8H → 1H	1.82	58.15	2.36×10^{2}	2.94×10^{-3}
8H → 7H	2.17	60.40	1.02×10^{0}	6.80×10^{-1}
7H → 6H	3.40	90.64	6.63×10^{-13}	1.05×10^{12}
6H ↔ 5H	3.88	101.57	2.61×10^{-18}	2.66×10^{17}
8H ↔ 9H	2.97	56.22	3.88×10^{-05}	1.79×10^{4}

Table S7. Structures, relative electronic energies (ΔE) and Gibbs free energies (ΔG) (at 298.15 K) computed at the B97-1/def2-TZVP level for isocyanoaniline and its prototropic tautomers. [a]

***Anti-imino* forms**	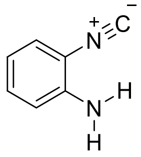	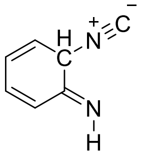	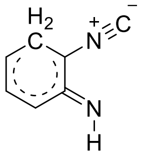	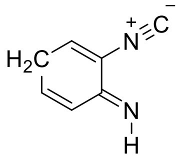	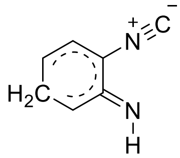	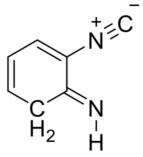
Name	**ICA**	**ortho-(NC)**	**meta-(NC)**	**para**	**meta-(H)**	**ortho-(H)**
ΔE	0.00	158.88	303.12	123.40	308.34	131.22
ΔG	0.00	166.98	311.87	131.76	317.10	140.74

***Syn-imino* forms**	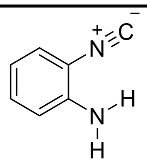	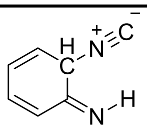	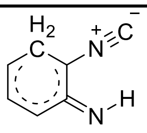	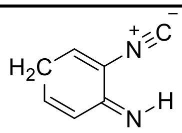	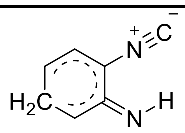	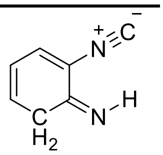
Name	**ICA**	**ortho-(NC)**	**meta-(NC)**	**para**	**meta-(H)**	**ortho-(H)**
ΔE	0.00	156.32	292.59	121.14	296.64	127.98
ΔG	0.00	164.88	300.67	129.09	304.95	137.13

[a] Relative energies in $kJ\ mol^{-1}$, absolute computed energies (in Hartree) for the amino-tautomer are: E=−379.8498528, G=−379.735318. Abbreviations: ICA = isocyanoaniline; *ortho-*, *meta- para-* are defined with respect to the imino group; suffix in ortho-(H) and ortho-(NC) indicates the substituent in the respective ortho position of the isocyanoanilinyl radical (before recombination with the H atom). Suffixes -(H) or -(NC) in *meta*-positions match the respective suffix of the vicinal *ortho*-position. See also Figure S8.

Table S8. Electronic (EE), zero-point corrected (EE+ZPE), and Gibbs free energies (E Gibbs, at 298.15 K) computed for the isomers discussed in this work. [a]

Isomer	EE	EE+ZPE	E Gibbs	Sym.	Shown in	Shown in
1*H*-BzIm (3*H*-BzIm)	-379.898942	-379.781308	-379.811503	C_s	Table 1	Fig. S1
2*H*-BzIm	-379.847617	-379.731599	-379.761303	C_{2v}	Table 1	Fig. 8
4*H*-BzIm (7*H*-BzIm)	-379.843870	-379.728173	-379.758819	C_s	Table 1	Fig. 8
5*H*-BzIm (6*H*-BzIm)	-379.846552	-379.730674	-379.761166	C_s	Table 1	Fig. 8
8*H*-BzIm (9*H*-BzIm)	-379.828615	-379.712991	-379.743401	C_1	Table 1	Fig. 8
Species **12** (ICA)	-379.849855	-379.735325	-379.767165	C_1	Chart 2	Fig. 9
Species **13** (syn-rad.)	-379.226273	-379.124355	-379.156650	C_s	Chart 2	Fig. 9
Species **14** (anti-rad.)	-379.223412	-379.121676	-379.153994	C_s	Chart 2	Fig. 9
Species **15** (syn-)	-379.802852	-379.689176	-379.721401	C_s	Chart 2	Fig. 9
Species **16** (anti-)	-379.799667	-379.686150	-379.718470	C_s	Chart 2	Fig. 9
Species **17** (syn-)	-379.799873	-379.686574	-379.719908	C_1	Chart 2	Fig. 9
Species **18** (anti-)	-379.796248	-379.683089	-379.716839	C_1	Chart 2	Fig. 9
Species **19** (syn-)	-379.789337	-379.675779	-379.708187	C_1	Chart 2	Fig. 9
Species **20** (anti-)	-379.786252	-379.672517	-379.705370	C_1	Chart 2	Fig. 9
Species **21** (radical)	-379.230477	-379.127256	-379.158123	C_s	Scheme 4	Fig. 7
Species **24** (syn-)	-379.779305	-379.666933	-379.698931	C_1	Scheme S1	Fig. S7
Species **24** (anti-)	-379.780871	-379.668621	-379.700684	C_1	Scheme S1	Fig. S7
Species **25** (syn-)	-379.771399	-379.658551	-379.690339	C_1	Scheme S1	
Species **25** (anti-)	-379.772099	-379.659314	-379.690947	C_1	Scheme S1	
Species **26** (syn-C)	-379.779793	-379.668494	-379.701212	C_s	Scheme S1	
Species **26** (anti-C)	-379.773645	-379.662480	-379.695487	C_s	Scheme S1	
Species **26** (syn-T)	-379.774320	-379.663314	-379.697181	C_s	Scheme S1	
Species **26** (anti-T)	-379.778861	-379.667564	-379.700649	C_s	Scheme S1	
Species **27** (syn-)	-379.811713	-379.698232	-379.730588	C_1	Scheme S1	Fig. S7
Species **27** (anti-)	-379.814153	-379.700719	-379.732938	C_1	Scheme S1	Fig. S7
Syn-meta-H	-379.732416	-379.622332	-379.655288	C_s	Table S7	Fig. 9
Syn-meta-(NC)	-379.734398	-379.623879	-379.656996	C_s	Table S7	Fig. 9
Anti-meta-H	-379.729077	-379.619169	-379.652432	C_s	Table S7	Fig. 9
Anti-meta-(NC)	-379.731064	-379.620796	-379.654544	C_s	Table S7	Fig. 9

[a] Absolute energies (in Hartree) computed at the B97-1/def2-TZVP level of theory. The two last columns indicate some parts of this work in which the respective structures are presented graphically. All structures listed in this table were optimized using tight convergence criteria and belong to true energy minima (have zero imaginary frequencies in the respective Hessian matrices). The respective optimized Cartesian coordinates are collected in Table S9.

Table S9. Cartesian coordinates (Å) of selected isomers and photoproducts of BzIm, optimized at the B97-1/def2-TZVP level of theory. If not stated otherwise, the species belong to the singlet manifold.

1*H*-BzIm (or 3*H*-BzIm), Cs (Imaginary Freq.: 0)
(also designated as species **11** in Scheme 4)

Atom	X	Y	Z
N	-1.1259700	1.5360020	0.0000000
C	-2.2113640	0.6953090	0.0000000
N	-1.8930500	-0.5700620	0.0000000
C	0.3813200	-1.6672480	0.0000000
C	1.7425040	-1.3945590	0.0000000
C	2.2274600	-0.0725480	0.0000000
C	1.3659630	1.0179540	0.0000000
C	0.0000000	0.7359860	0.0000000
C	-0.5046700	-0.5849120	0.0000000
H	-1.1521560	2.5413300	0.0000000
H	-3.2212540	1.0823990	0.0000000
H	0.0039420	-2.6833800	0.0000000
H	2.4521950	-2.2144930	0.0000000
H	3.2983100	0.0984830	0.0000000
H	1.7448160	2.0341910	0.0000000

2*H*-BzIm, C2v (Imaginary Freq.: 0)

Atom	X	Y	Z
N	0.0000000	1.1963650	1.5094290
C	0.0000000	0.0000000	2.3282630
N	0.0000000	-1.1963650	1.5094290
C	0.0000000	-1.4604770	-0.9541180
C	0.0000000	-0.7278960	-2.0953520
C	0.0000000	0.7278960	-2.0953520
C	0.0000000	1.4604770	-0.9541180
C	0.0000000	0.7415720	0.2957880
C	0.0000000	-0.7415720	0.2957880
H	0.8790640	0.0000000	2.9879070
H	-0.8790640	0.0000000	2.9879070
H	0.0000000	-2.5441670	-0.9591590
H	0.0000000	-1.2296380	-3.0574520
H	0.0000000	1.2296380	-3.0574520
H	0.0000000	2.5441670	-0.9591590

4*H*-BzIm (or 7*H*-BzIm), Cs (Imaginary Freq.: 0)
(also designated as species **22** in Scheme 4)

Atom	X	Y	Z
N	-0.9032860	1.7309050	0.0000000
C	-2.1057670	0.9994690	0.0000000
N	-2.0243300	-0.3059580	0.0000000
C	0.0732900	-1.6636030	0.0000000
C	1.5212980	-1.5930290	0.0000000
C	2.1816580	-0.4152960	0.0000000
C	1.4816400	0.9088730	0.0000000
C	0.0000000	0.7886330	0.0000000
C	-0.6525450	-0.5154850	0.0000000
H	1.8117130	1.4995730	0.8674840
H	-3.0533870	1.5249670	0.0000000
H	-0.4197020	-2.6308730	0.0000000
H	2.0788580	-2.5232470	0.0000000
H	3.2666750	-0.4019870	0.0000000
H	1.8117130	1.4995730	-0.8674840

6*H*-BzIm (or 5*H*-BzIm), Cs (Imaginary Freq.: 0)
(also designated as species **23** in Scheme 4)

Atom	X	Y	Z
N	1.9482030	-0.3516140	0.0000000
C	2.0916200	1.0451350	0.0000000
N	1.0086030	1.7760780	0.0000000
C	-1.3396370	0.9436030	0.0000000
C	-2.1922540	-0.2806240	0.0000000
C	-1.4805130	-1.5974900	0.0000000
C	-0.1369290	-1.7090670	0.0000000
C	0.6462760	-0.5038770	0.0000000
C	0.0000000	0.8143090	0.0000000
H	-2.8756620	-0.2421310	0.8642440
H	3.0842620	1.4808160	0.0000000
H	-1.8179160	1.9184110	0.0000000
H	-2.8756620	-0.2421310	-0.8642440
H	-2.1047580	-2.4855670	0.0000000
H	0.3607060	-2.6725910	0.0000000

8*H*-BzIm (or 9*H*-BzIm), C1 (Imaginary Freq.: 0)

Atom	X	Y	Z
N	1.7027670	-1.0971580	0.0145310
C	2.2741020	0.0398570	-0.2039870
N	1.4904250	1.2123760	-0.1522690
C	-0.9612300	1.4484880	0.1211870
C	-2.0759760	0.6867970	-0.0378250
C	-2.0286360	-0.7614220	-0.1925150
C	-0.8782850	-1.4555390	-0.0823770
C	0.3410650	-0.7185220	0.3590560
C	0.3003640	0.7619620	0.1264540
H	3.3324160	0.1235150	-0.4273820
H	-0.9971410	2.5316780	0.1180120
H	-3.0446820	1.1700150	-0.1165910
H	-2.9547060	-1.2781190	-0.4203390
H	-0.8353160	-2.5330020	-0.1963440
H	0.3186590	-0.8303440	1.4668410

Species **12** (ICA), C1 (Imaginary Freq.: 0)

Atom	X	Y	Z
N	-0.9596490	1.9321960	-0.0677890
C	-2.9811210	-0.8647040	0.0083320
N	-1.8183210	-0.7055200	0.0004490
C	0.4612640	-1.4905720	-0.0019770
C	1.8224250	-1.2217950	0.0031090
C	2.2534350	0.1071330	0.0067350
C	1.3415060	1.1529450	0.0014780
C	-0.0410580	0.9072270	-0.0062300
C	-0.4609110	-0.4416070	-0.0055970
H	-0.6474130	2.8400440	0.2354980
H	-1.9110340	1.7120400	0.1841620
H	0.0876450	-2.5077500	-0.0004860
H	2.5378600	-2.0348400	0.0063500
H	3.3144970	0.3314780	0.0129390
H	1.6909890	2.1805300	-0.0021750

Species **13**, Cs (Imaginary Freq.: 0)
(2-isocyanoanilinyl radical, *syn*-isomer),
doublet

Atom	X	Y	Z
N	-2.1021030	-0.6687220	0.0000000
C	-0.6853800	1.8740380	0.0000000
N	-1.2617970	2.8769990	0.0000000
C	1.3903710	0.5837670	0.0000000
C	2.0605640	-0.6412030	0.0000000
C	1.3372250	-1.8454700	0.0000000
C	-0.0402510	-1.8319370	0.0000000
C	-0.7770100	-0.6026810	0.0000000
C	0.0000000	0.6222520	0.0000000
H	-2.5077640	0.2720530	0.0000000
H	1.9486010	1.5127610	0.0000000
H	3.1442030	-0.6573630	0.0000000
H	1.8691090	-2.7904040	0.0000000
H	-0.6199720	-2.7475770	0.0000000

Species **14**, Cs (Imaginary Freq.: 0)
(2-isocyanoanilinyl radical, *anti*-isomer),
doublet

Atom	X	Y	Z
N	-2.1258300	-0.4539800	0.0000000
C	-0.6347550	1.9058660	0.0000000
N	-1.0937630	2.9665610	0.0000000
C	1.3889670	0.5487160	0.0000000
C	2.0287770	-0.6921400	0.0000000
C	1.2755290	-1.8780190	0.0000000
C	-0.1009570	-1.8266860	0.0000000
C	-0.8045770	-0.5764440	0.0000000
C	0.0000000	0.6260320	0.0000000
H	-2.5565260	-1.3822510	0.0000000
H	1.9699080	1.4634560	0.0000000
H	3.1115960	-0.7366960	0.0000000
H	1.7823920	-2.8367490	0.0000000
H	-0.6881220	-2.7397790	0.0000000

Species **15**, Cs (Imaginary Freq.: 0)
(2-isocyanocyclohexa-2,5-dienimine, *syn*-)

Atom	X	Y	Z
C	-2.0700320	-0.6982640	0.0000000
C	-1.3404530	0.6027030	0.0000000
C	0.0000000	0.6775010	0.0000000
C	0.8743650	-0.5313830	0.0000000
C	0.1572200	-1.8129120	0.0000000
C	-1.1768880	-1.8987680	0.0000000
N	0.6382630	1.9030030	0.0000000
N	2.1528110	-0.5221830	0.0000000
C	1.2613660	2.8977180	0.0000000
H	2.5183400	0.4327490	0.0000000
H	0.7859550	-2.6963950	0.0000000
H	-1.6588640	-2.8718200	0.0000000
H	-2.7467720	-0.7341550	0.8679210
H	-1.9228640	1.5184660	0.0000000
H	-2.7467720	-0.7341550	-0.8679210

Species **16**, Cs (Imaginary Freq.: 0)
(2-isocyanocyclohexa-2,5-dienimine, *anti*-)

Atom	X	Y	Z
C	-2.0548940	-0.7186490	0.0000000
C	-1.3399370	0.5908830	0.0000000
C	0.0000000	0.6893570	0.0000000
C	0.8842940	-0.5058740	0.0000000
C	0.1842030	-1.8003660	0.0000000
C	-1.1484670	-1.9074000	0.0000000
N	0.6097430	1.9257280	0.0000000
N	2.1533960	-0.3443680	0.0000000
C	1.1799160	2.9507580	0.0000000
H	2.6281950	-1.2493420	0.0000000
H	0.8110220	-2.6878610	0.0000000
H	-1.6157390	-2.8878890	0.0000000
H	-2.7307630	-0.7674930	0.8679590
H	-1.9346020	1.4982920	0.0000000
H	-2.7307630	-0.7674930	-0.8679590

Species **17**, C1 (Imaginary Freq.: 0)
(2-isocyanocyclohexa-2,4-dienimine, *syn*-)

Atom	X	Y	Z
C	1.7037050	-1.3265800	-0.0447390
C	0.2618330	-1.4776590	-0.0102040
C	-0.5662530	-0.4072290	0.0152540
C	-0.0544770	0.9870320	-0.0061720
C	1.4573120	1.1436770	0.0815280
C	2.2683930	-0.1106640	-0.0115380
N	-1.9336650	-0.5717500	0.0241900
N	-0.7743840	2.0344930	-0.0849880
C	-3.1075000	-0.6158920	0.0306810
H	-1.7697880	1.8032660	-0.1116920
H	1.7722670	1.8624080	-0.6832750
H	3.3484700	-0.0047750	-0.0315950
H	2.3112460	-2.2227400	-0.0963020
H	-0.1679390	-2.4734230	-0.0166150
H	1.6840100	1.6399520	1.0362100

Species **18**, C1 (Imaginary Freq.: 0)
(2-isocyanocyclohexa-2,4-dienimine, *anti*-)

Atom	X	Y	Z
C	1.6585700	-1.3697000	-0.0297060
C	0.2118910	-1.4807910	-0.0072550
C	-0.5916700	-0.3910340	0.0075290
C	-0.0412860	0.9838030	-0.0055620
C	1.4802940	1.1044000	0.0566320
C	2.2615240	-0.1734830	-0.0068200
N	-1.9588450	-0.5305030	0.0140730
N	-0.8462290	1.9712320	-0.0561990
C	-3.1311320	-0.5902750	0.0213100
H	-0.3391890	2.8588530	-0.0685160
H	1.8075970	1.7764600	-0.7469750
H	3.3441040	-0.0972160	-0.0204530
H	2.2407500	-2.2835610	-0.0648100
H	-0.2437540	-2.4645500	-0.0104030
H	1.7368650	1.6274100	0.9892800

Species **19**, C1 (Imaginary Freq.: 0)
(6-isocyanocyclohexa-2,4-dienimine, *syn*-)

Atom	X	Y	Z
C	1.6435980	-1.3228000	-0.1774940
C	0.3316240	-1.5114130	0.0019140
C	-0.5421760	-0.3591120	0.4431200
C	0.0139690	1.0336090	0.0686180
C	1.4784700	1.1131480	0.0503240
C	2.2373110	0.0068130	-0.0688130
N	-1.8944730	-0.5168120	0.0401390
N	-0.6845530	2.0697760	-0.1661640
C	-3.0120050	-0.6135150	-0.2937380
H	-1.6850660	1.8572730	-0.1807950
H	1.9052640	2.1092050	0.0300690
H	3.3156960	0.0966270	-0.1467000
H	2.2855570	-2.1610980	-0.4248590
H	-0.1371910	-2.4844770	-0.0864410
H	-0.5358320	-0.3686600	1.5473160

Species **20**, C1 (Imaginary Freq.: 0)
(6-isocyanocyclohexa-2,4-dienimine, *anti*-)

Atom	X	Y	Z
C	1.4958160	-1.3646540	0.1397080
C	0.2793510	-1.4113790	0.6927760
C	-0.6597840	-0.2259040	0.6277660
C	0.0120890	1.0889270	0.1856510
C	1.2772620	0.9808930	-0.5431280
C	1.9802900	-0.1669620	-0.5412350
N	-1.7326430	-0.5382220	-0.2704580
N	-0.5802280	2.1697340	0.5111110
C	-2.5950080	-0.8141900	-1.0121050
H	-0.0801770	2.9837070	0.1445750
H	1.6552040	1.8739130	-1.0315250
H	2.9410780	-0.2157850	-1.0431820
H	2.1435570	-2.2339450	0.1748370
H	-0.1004140	-2.3099170	1.1660830
H	-1.1092480	-0.0389540	1.6080480

Species *syn*-**24**, C1 (Imaginary Freq.: 0)
(*syn*-imino-nitrile-ylide)

Atom	X	Y	Z
N	-0.6657150	2.1064210	-0.0064650
C	-2.9675510	-0.5814010	-0.1499780
N	-1.7806130	-0.5417760	0.0262140
C	0.3919270	-1.5069050	0.0020660
C	1.7344870	-1.3212310	-0.0028890
C	2.2842320	0.0132050	0.0022690
C	1.4932440	1.1110330	0.0035890
C	0.0362340	1.0239350	0.0054860
C	-0.4901450	-0.3704790	0.0129670
H	-1.6677600	1.9055870	-0.0002760
H	-3.7263760	-0.6379120	0.6323680
H	-0.0477880	-2.4978990	-0.0034180
H	2.4028850	-2.1740400	-0.0125580
H	3.3631080	0.1295050	0.0016330
H	1.9056490	2.1133080	0.0029410

Species *anti*-**24**, C1 (Imaginary Freq.: 0)
(*anti*-imino-nitrile-ylide)

Atom	X	Y	Z
N	-0.8475350	1.9750620	0.0391600
C	-2.9527800	-0.5673530	-0.1704530
N	-1.7774920	-0.5791910	0.0144300
C	0.4144690	-1.5118060	0.0286630
C	1.7575580	-1.3053240	0.0108220
C	2.2745370	0.0370890	-0.0196730
C	1.4587580	1.1209590	-0.0271870
C	0.0046380	1.0026610	0.0100730
C	-0.4819470	-0.3956120	0.0203570
H	-0.3579480	2.8696130	0.0051030
H	-3.7760850	-0.5731490	0.5385190
H	-0.0073970	-2.5105650	0.0420290
H	2.4425900	-2.1443730	0.0133690
H	3.3506620	0.1788540	-0.0387900
H	1.8719680	2.1248470	-0.0509710

Species *syn*-**25**, C1 (Imaginary Freq.: 0)
(*syn*-imino-spiro-azirine)

Atom	X	Y	Z
N	0.8898200	2.0483550	0.1101480
C	1.9252660	-0.7021340	-0.6126000
N	2.0321550	-0.6737350	0.6247390
C	-0.3872460	-1.4884690	0.0865700
C	-1.7030370	-1.2135050	0.0559070
C	-2.1709080	0.1594310	-0.0620050
C	-1.3176830	1.2031920	-0.0913000
C	0.1321630	1.0185250	0.0021440
C	0.6058640	-0.4128100	-0.0312770
H	1.8696560	1.7755970	0.2157430
H	2.5280260	-0.8304100	-1.5056390
H	-0.0190420	-2.5049580	0.1792370
H	-2.4320050	-2.0132760	0.1229040
H	-3.2408140	0.3367470	-0.1028670
H	-1.6661610	2.2285850	-0.1382150

Species *anti*-**25**, C1 (Imaginary Freq.: 0)
(*anti*-imino-spiro-azirine)

Atom	X	Y	Z
N	1.0053970	1.9441350	-0.1637010
C	2.0323800	-0.6360290	-0.4028200
N	1.9057850	-0.7397780	0.8257880
C	-0.3697030	-1.4913430	-0.1569750
C	-1.6884840	-1.2257790	-0.0903380
C	-2.1572440	0.1386950	0.0743320
C	-1.3066240	1.1863540	0.1225960
C	0.1372290	1.0055520	-0.0337860
C	0.6166030	-0.4134810	-0.0809180
H	0.5574180	2.8597620	-0.0982920
H	2.7957190	-0.6452420	-1.1723180
H	-0.0010110	-2.5082390	-0.2405000
H	-2.4151910	-2.0287150	-0.1369810
H	-3.2255010	0.3084440	0.1670790
H	-1.6746560	2.1996770	0.2538600

Species *syn*-**27**, C1 (Imaginary Freq.: 0)
(*syn*-imino-ketenimine)

Atom	X	Y	Z
N	0.5186940	2.1684510	-0.0220000
C	1.8205900	-0.4613030	0.0176790
N	3.0219610	-0.5451130	-0.0994030
C	-0.3508850	-1.5141030	-0.0170760
C	-1.6952160	-1.3906590	-0.0061210
C	-2.3066800	-0.0794970	0.0196880
C	-1.5707160	1.0535230	0.0225340
C	-0.1094150	1.0436740	-0.0030080
C	0.4936220	-0.3296230	-0.0085750
H	3.6155570	-0.5968990	0.7284300
H	1.5321480	2.0414590	-0.0492940
H	0.1259140	-2.4876030	-0.0351950
H	-2.3245210	-2.2727340	-0.0169060
H	-3.3899290	-0.0141890	0.0346380
H	-2.0315620	2.0345270	0.0374260

Species *anti*-**27**, C1 (Imaginary Freq.: 0)
(*anti*-imino-ketenimine)

Atom	X	Y	Z
N	0.6465090	2.0753540	0.0564520
C	1.8340860	-0.4277040	-0.0003060
N	3.0232950	-0.5297650	-0.1589290
C	-0.3239160	-1.5156270	0.0418770
C	-1.6730740	-1.4135670	0.0213970
C	-2.2963940	-0.1120250	-0.0279350
C	-1.5725190	1.0317640	-0.0421510
C	-0.1114680	1.0326790	0.0079460
C	0.4990030	-0.3245620	0.0139970
H	3.6676500	-0.5246940	0.6293050
H	0.0801740	2.9238270	0.0312940
H	0.1660050	-2.4825450	0.0660890
H	-2.2918170	-2.3026750	0.0325050
H	-3.3804670	-0.0581050	-0.0541220
H	-2.0644820	1.9993330	-0.0766790

Species *syn-cis*-**26**, Cs (Imaginary Freq.: 0)
(*syn*-imino-*cis*-vinyl-nitrene), triplet

Atom	X	Y	Z
N	-1.9530070	-0.8454380	0.0000000
C	-0.7869320	1.9074240	0.0000000
N	-2.0383080	2.0118460	0.0000000
C	1.3889430	0.7456650	0.0000000
C	2.1811250	-0.4030370	0.0000000
C	1.5802190	-1.6745690	0.0000000
C	0.2096480	-1.7922900	0.0000000
C	-0.6476710	-0.6372790	0.0000000
C	0.0000000	0.6637300	0.0000000
H	-2.4612500	0.0495840	0.0000000
H	-0.1967130	2.8389740	0.0000000
H	1.8619220	1.7230370	0.0000000
H	3.2613470	-0.3118740	0.0000000
H	2.2018020	-2.5631870	0.0000000
H	-0.2799000	-2.7592650	0.0000000

Species *anti-cis*-**26**, Cs (Imaginary Freq.: 0)
(*anti*-imino-*cis*-vinyl-nitrene), triplet

Atom	X	Y	Z
N	-1.9795820	-0.6708090	0.0000000
C	-0.7580840	1.9316880	0.0000000
N	-2.0017950	2.1045150	0.0000000
C	1.3910820	0.7285540	0.0000000
C	2.1704000	-0.4277230	0.0000000
C	1.5549830	-1.6929280	0.0000000
C	0.1834590	-1.7904450	0.0000000
C	-0.6583890	-0.6226070	0.0000000
C	0.0000000	0.6688550	0.0000000
H	-2.2923740	-1.6454140	0.0000000
H	-0.1292530	2.8390130	0.0000000
H	1.8752920	1.7002260	0.0000000
H	3.2516890	-0.3503090	0.0000000
H	2.1649280	-2.5897150	0.0000000
H	-0.3013500	-2.7621090	0.0000000

Species *syn-trans*-**26**, Cs (Imaginary Freq.: 0)
(*syn*-imino-*trans*-vinyl-nitrene), triplet

Atom	X	Y	Z
N	2.3021240	-0.3475640	0.0000000
C	0.4207020	2.0034240	0.0000000
N	-0.3461080	2.9977170	0.0000000
C	-1.3602800	0.2774390	0.0000000
C	-1.7970780	-1.0424340	0.0000000
C	-0.8653980	-2.1000150	0.0000000
C	0.4799410	-1.8301690	0.0000000
C	0.9824040	-0.4806540	0.0000000
C	0.0000000	0.5908020	0.0000000
H	2.5801920	0.6353910	0.0000000
H	1.5004750	2.2185190	0.0000000
H	-2.0812010	1.0876300	0.0000000
H	-2.8600140	-1.2560360	0.0000000
H	-1.2149500	-3.1265810	0.0000000
H	1.2216510	-2.6203640	0.0000000

Species *anti-trans*-**26**, Cs (Imaginary Freq.: 0)
(*anti*-imino-*trans*-vinyl-nitrene), triplet

Atom	X	Y	Z
N	2.2462530	-0.2204870	0.0000000
C	0.5151630	1.9804530	0.0000000
N	-0.1933140	3.0147740	0.0000000
C	-1.3677100	0.3298430	0.0000000
C	-1.8401780	-0.9786360	0.0000000
C	-0.9383280	-2.0627620	0.0000000
C	0.4158010	-1.8343530	0.0000000
C	0.9491400	-0.4977470	0.0000000
C	0.0000000	0.5967310	0.0000000
H	2.7866980	-1.0882510	0.0000000
H	1.6112720	2.0722950	0.0000000
H	-2.0629950	1.1619220	0.0000000
H	-2.9080510	-1.1659260	0.0000000
H	-1.3187150	-3.0784980	0.0000000
H	1.1178900	-2.6627260	0.0000000

Species *syn*-meta-H, Cs (Imaginary Freq.: 0)
(marked 6*H* in Figure 9a)

Atom	X	Y	Z
C	2.0749620	-0.5865280	0.0000000
C	1.4092880	0.6058460	0.0000000
C	0.0000000	0.6587940	0.0000000
C	-0.9055460	-0.5162340	0.0000000
C	-0.1687020	-1.7330670	0.0000000
C	1.2882740	-1.8259910	0.0000000
N	-0.6022730	1.8786330	0.0000000
N	-2.2064710	-0.5310750	0.0000000
C	-1.2461110	2.8672540	0.0000000
H	-2.5879460	0.4144220	0.0000000
H	1.5884070	-2.4606320	0.8559790
H	1.5884070	-2.4606320	-0.8559790
H	3.1556520	-0.6400570	0.0000000
H	1.9466270	1.5480850	0.0000000
H	-0.7429340	-2.6545410	0.0000000

Species *anti*-meta-H, Cs (Imaginary Freq.: 0)
(marked 6*H* in Figure 9b)

Atom	X	Y	Z
C	2.0631330	-0.6014090	0.0000000
C	1.4079050	0.5975940	0.0000000
C	0.0000000	0.6626470	0.0000000
C	-0.9165280	-0.5023960	0.0000000
C	-0.1886340	-1.7290510	0.0000000
C	1.2706960	-1.8348000	0.0000000
N	-0.5792330	1.8889930	0.0000000
N	-2.2033590	-0.3183810	0.0000000
C	-1.1930140	2.8965080	0.0000000
H	-2.6859720	-1.2162680	0.0000000
H	1.5790180	-2.4635070	0.8574760
H	1.5790180	-2.4635070	-0.8574760
H	3.1438030	-0.6623060	0.0000000
H	1.9515230	1.5358080	0.0000000
H	-0.7505910	-2.6590710	0.0000000

Species *syn*-meta-(NC), Cs (Imagin. Freq.: 0)
(marked 4*H* in Figure 9a)

Atom	X	Y	Z
C	-1.9931300	-0.9048320	0.0000000
C	-1.4638930	0.4615620	0.0000000
C	0.0000000	0.6361670	0.0000000
C	0.9492360	-0.4707190	0.0000000
C	0.2663930	-1.7537550	0.0000000
C	-1.1149890	-1.9616860	0.0000000
N	0.4765590	1.9000870	0.0000000
N	2.2439340	-0.4332910	0.0000000
C	0.9875460	2.9663490	0.0000000
H	2.5842500	0.5279420	0.0000000
H	0.9290720	-2.6147500	0.0000000
H	-1.4875920	-2.9805800	0.0000000
H	-3.0669900	-1.0452900	0.0000000
H	-1.8945800	1.0032940	0.8615160
H	-1.8945800	1.0032940	-0.8615160

Species *anti*-meta-(NC), Cs (Imagin. Freq.: 0)
(marked 4*H* in Figure 9b)

Atom	X	Y	Z
C	-1.9859290	-0.9185330	0.0000000
C	-1.4618630	0.4506480	0.0000000
C	0.0000000	0.6400720	0.0000000
C	0.9591190	-0.4550050	0.0000000
C	0.2847080	-1.7469670	0.0000000
C	-1.0963780	-1.9647180	0.0000000
N	0.4540650	1.9085220	0.0000000
N	2.2340890	-0.2229330	0.0000000
C	0.9302800	2.9903610	0.0000000
H	2.7556520	-1.0985800	0.0000000
H	0.9351600	-2.6186110	0.0000000
H	-1.4594190	-2.9875150	0.0000000
H	-3.0582250	-1.0667970	0.0000000
H	-1.8849320	0.9986170	0.8607370
H	-1.8849320	0.9986170	-0.8607370

Species **21**, Cs (Imaginary Freq.: 0)
(benzimidazolyl radical), doublet

Atom	X	Y	Z
N	-0.9872300	1.6843570	0.0000000
C	-2.1427540	0.8748100	0.0000000
N	-1.9705510	-0.4154540	0.0000000
C	0.2323180	-1.6542850	0.0000000
C	1.6389880	-1.4468460	0.0000000
C	2.2072430	-0.1676870	0.0000000
C	1.3996080	0.9674900	0.0000000
C	0.0000000	0.7761500	0.0000000
C	-0.5668080	-0.5421650	0.0000000
H	-3.1213340	1.3387840	0.0000000
H	-0.1823840	-2.6558420	0.0000000
H	2.2928390	-2.3124990	0.0000000
H	3.2859800	-0.0654370	0.0000000
H	1.8177990	1.9678710	0.0000000

Figure S9. Twenty model chemistries (5 methods × 4 basis sets) comparing computed infrared spectrum of 1*H*-BzIm with the experimental infrared spectrum of benzimidazole monomers isolated in an Ar matrix, shown in six spectral ranges.

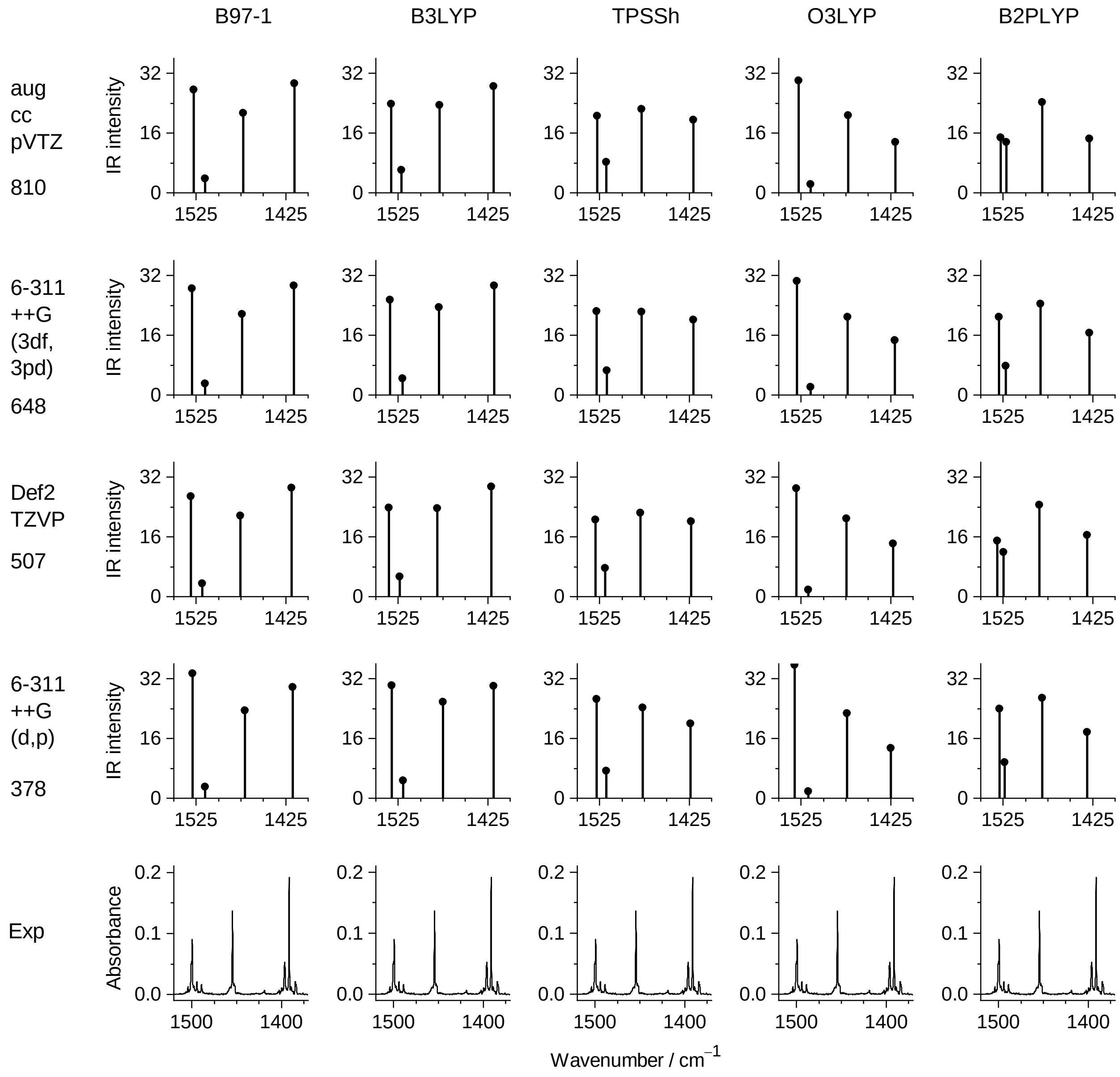

Figure S9. (Continued)

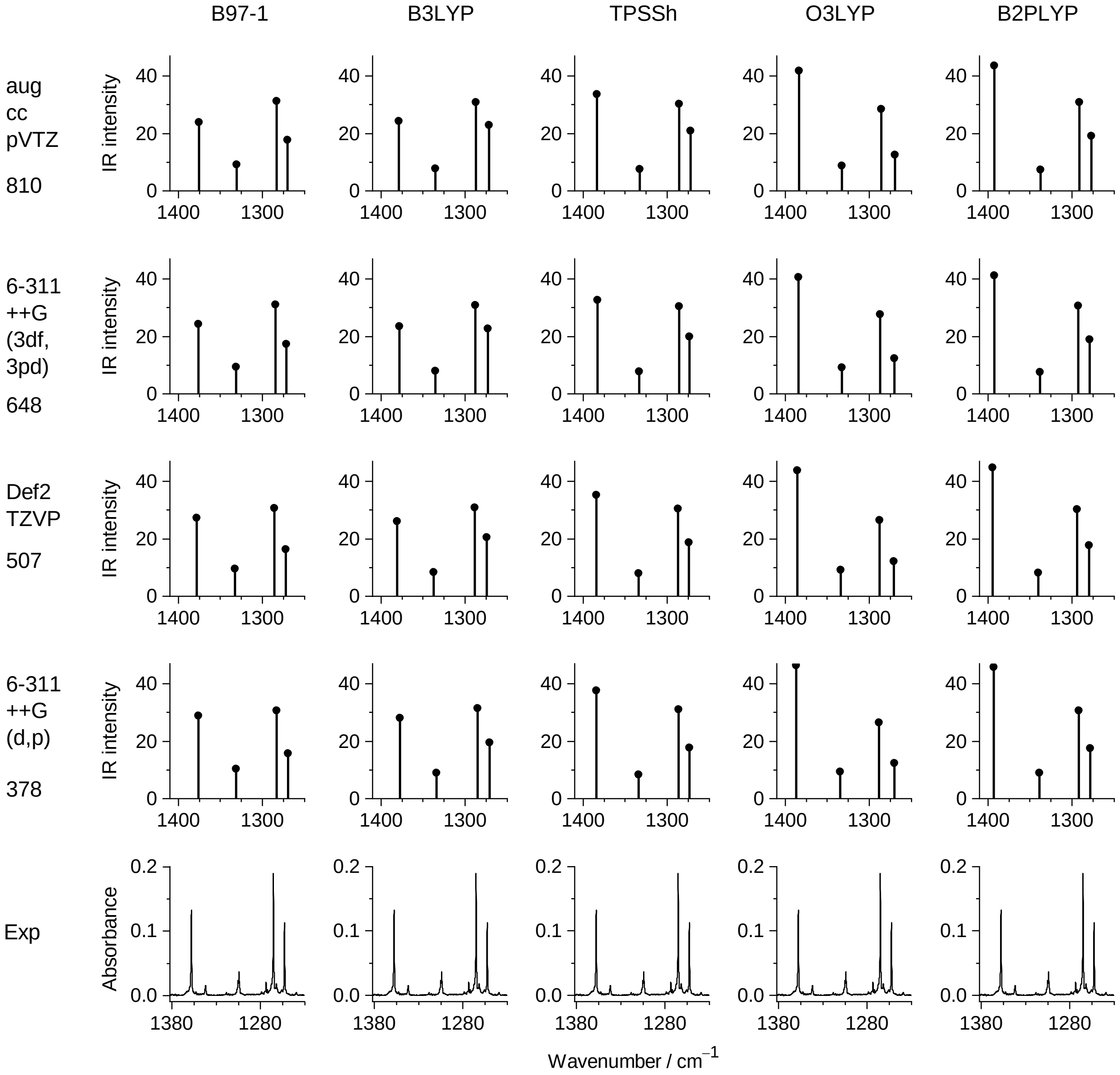

Figure S9. (Continued)

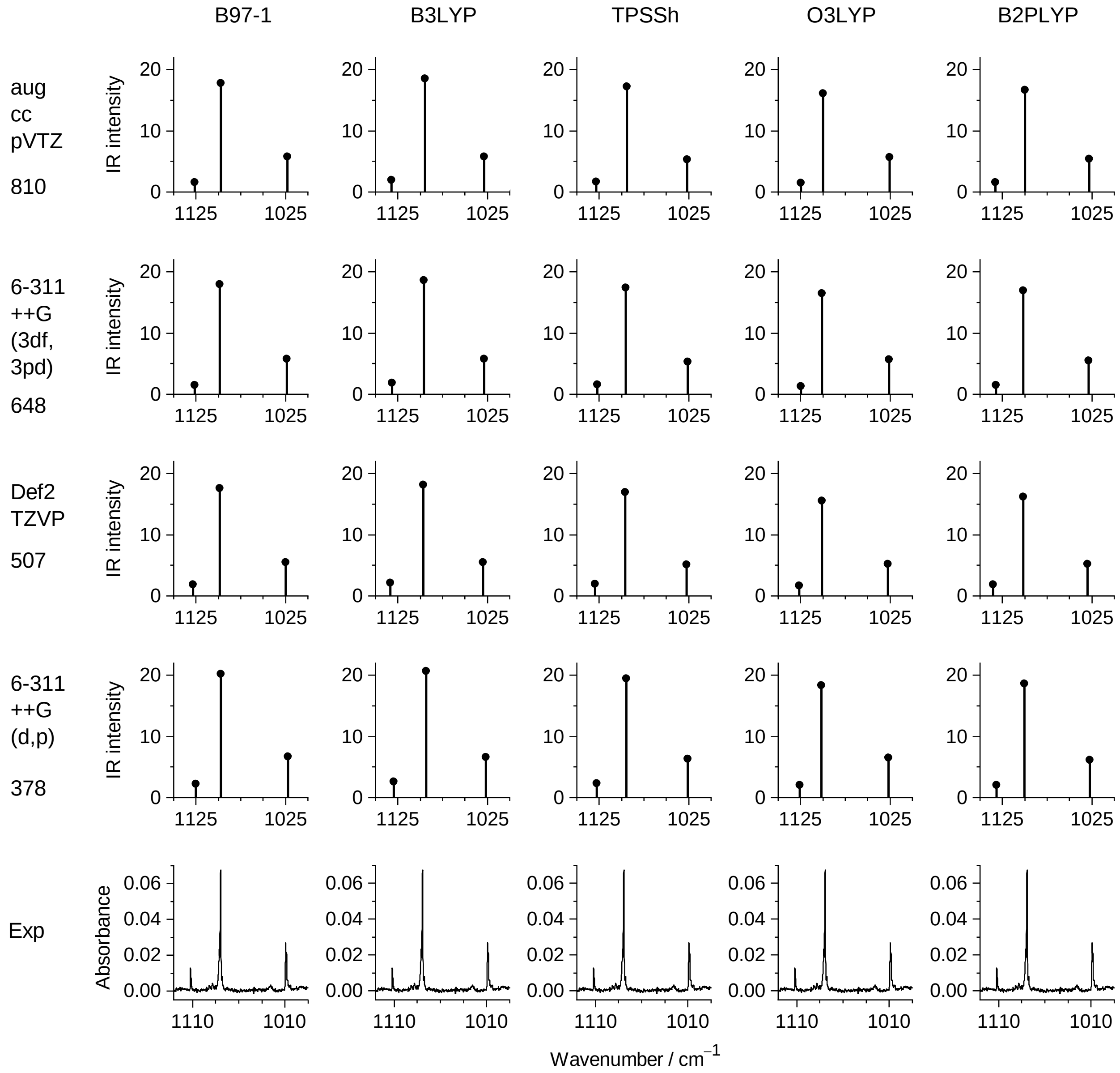

Figure S9. (Continued)

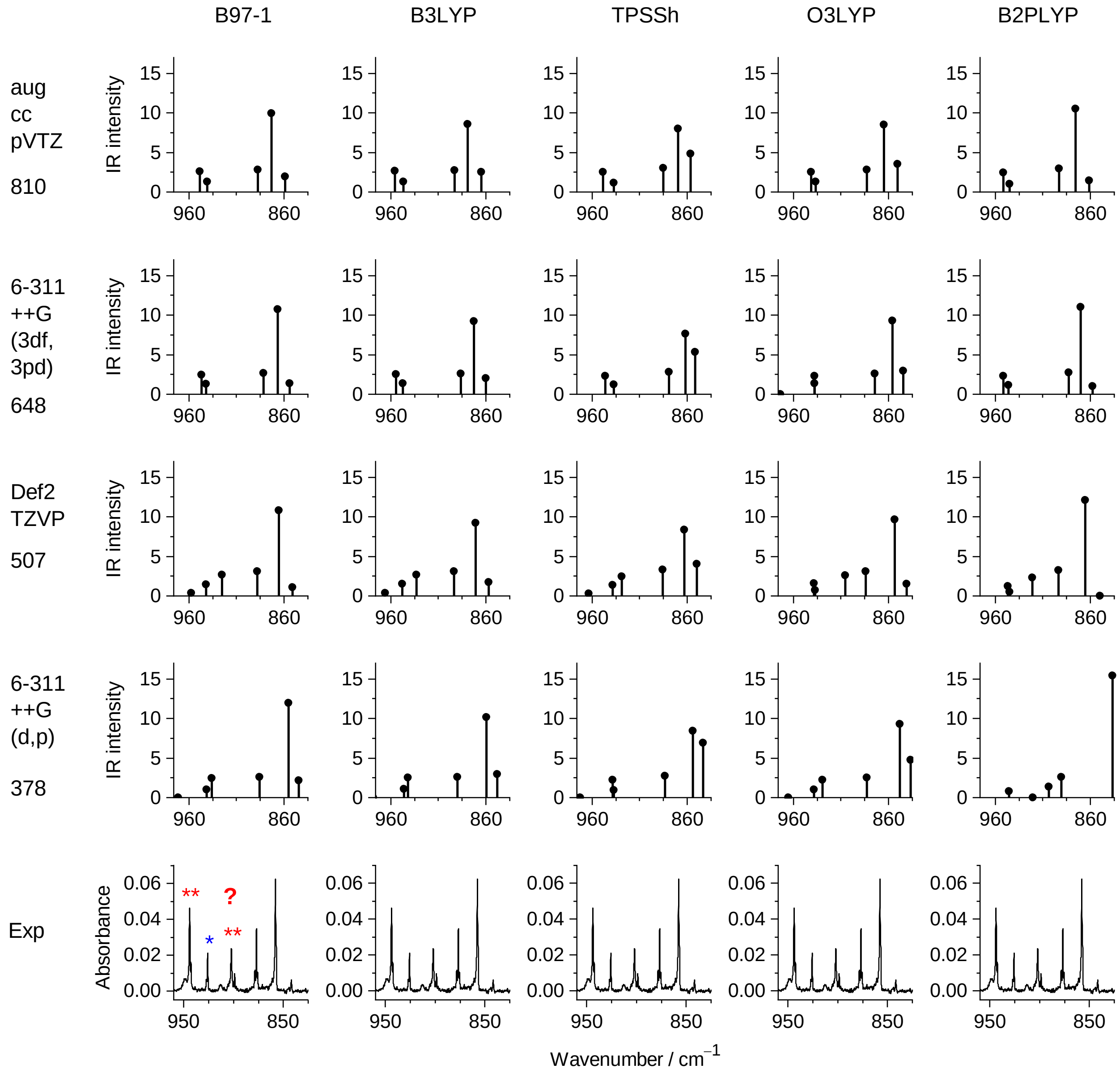

Figure S9. (Continued)

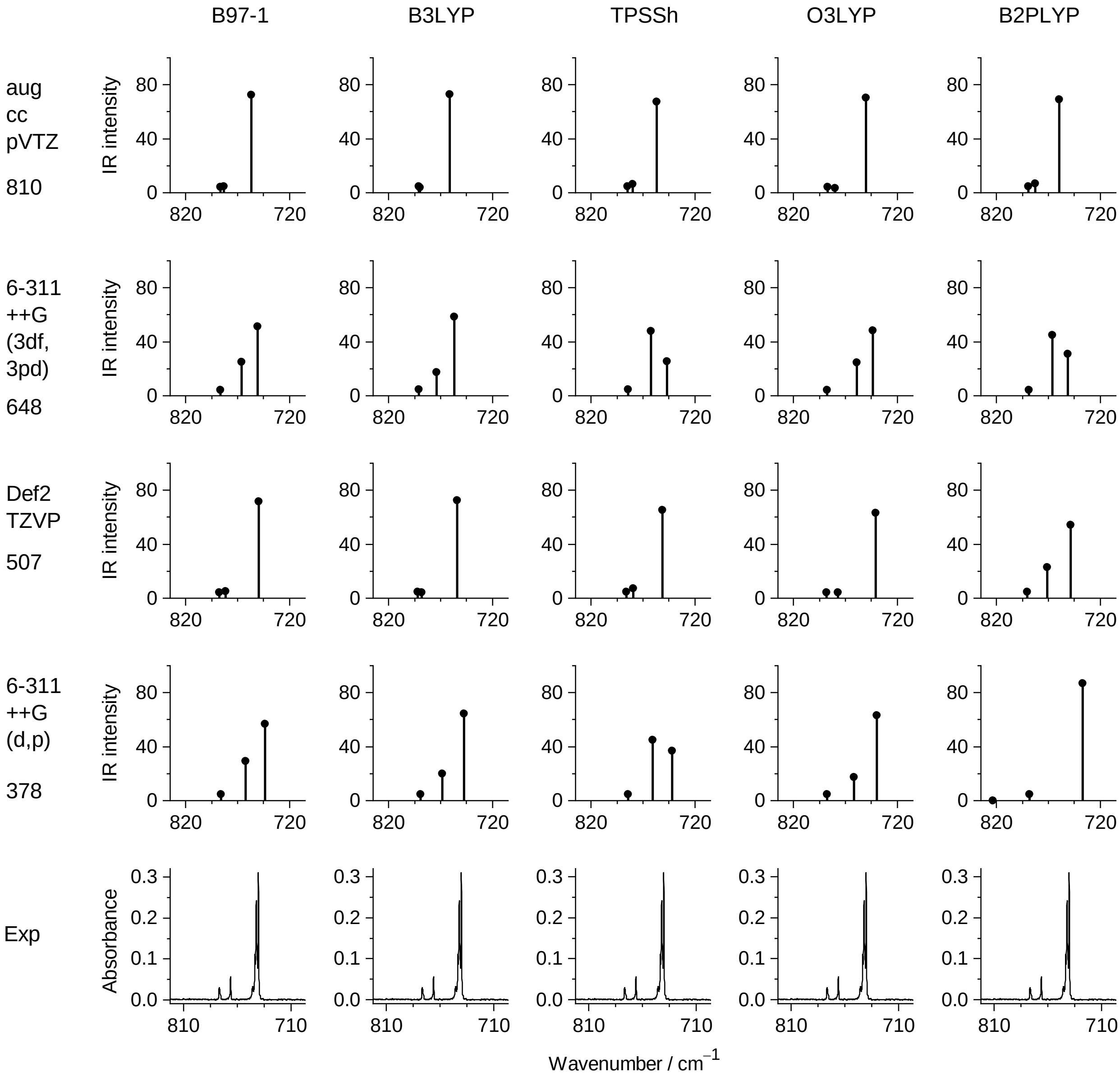

Figure S9. (Continued)

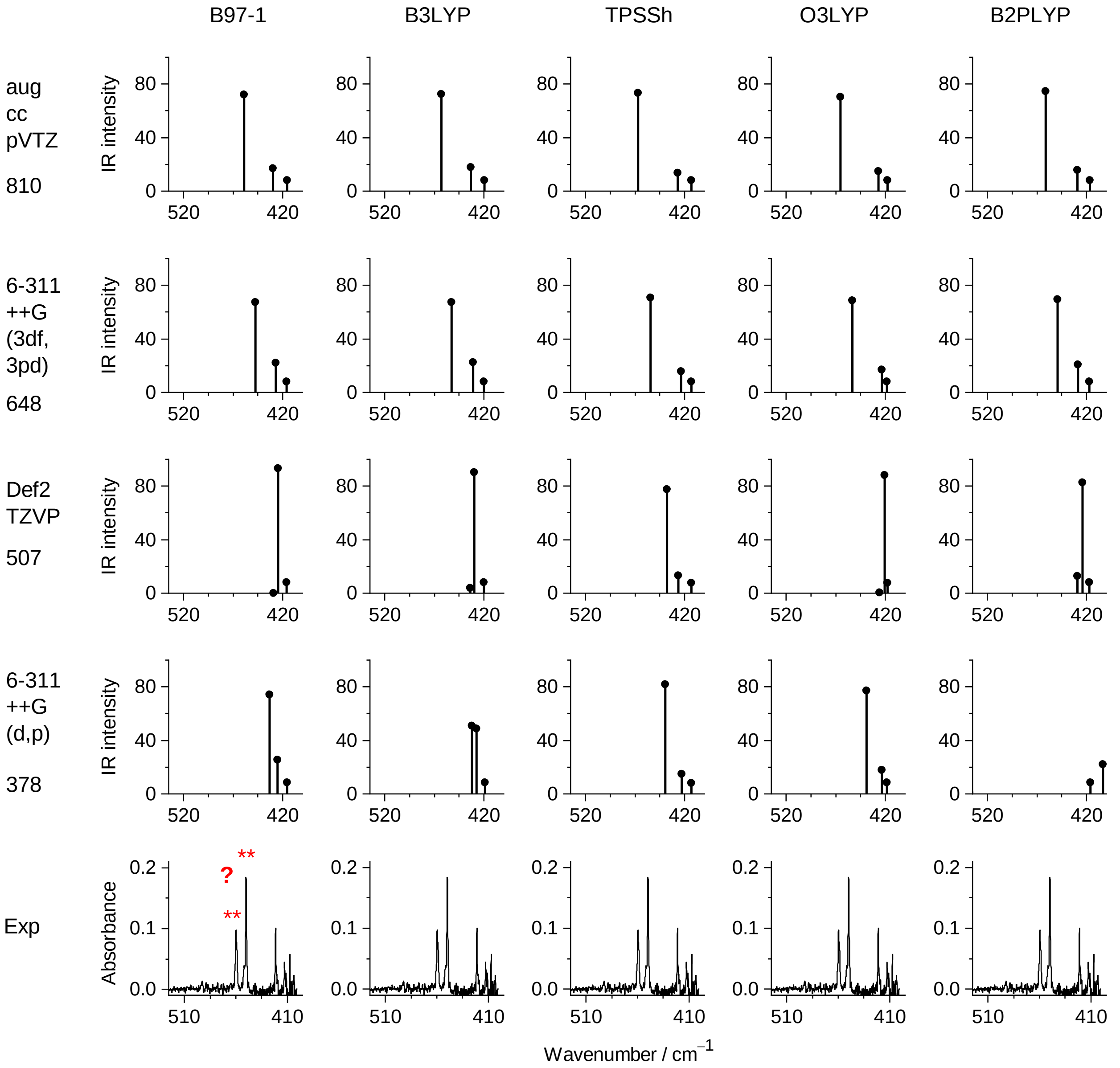

Supporting Information References:

(1) Kanwal, A.; Saddique, F. A.; Aslam, S.; Ahmad, M.; Zahoor, A. F.; Mohsin, N. U. Benzimidazole Ring System as a Privileged Template for Anticancer Agents. *Pharm. Chem. J.* **2018,** *51* (12), 1068-1077.

(2) Tahlan, S.; Kumar, S.; Kakkar, S.; Narasimhan, B. Benzimidazole scaffolds as promising antiproliferative agents: a review. *BMC Chem.* **2019,** *13*, 66, https://doi.org/10.1186/s13065-019-0579-6.

(3) Sharma, P.; LaRosa, C.; Antwi, J.; Govindarajan, R.; Werbovetz, K. A. Imidazoles as Potential Anticancer Agents: An Update on Recent Studies. *Molecules* **2021,** *26* (14), 4213, https://doi.org/10.3390/molecules26144213.

(4) Davidse, L. C. Benzimidazole Fungicides - Mechanism of Action and Biological Impact. *Annu. Rev. Phytopathol.* **1986,** *24*, 43-65, https://doi.org/10.1146/annurev.py.24.090186.000355.

(5) Kathiravan, M. K.; Salake, A. B.; Chothe, A. S.; Dudhe, P. B.; Watode, R. P.; Mukta, M. S.; Gadhwe, S. The biology and chemistry of antifungal agents: A review. *Bioorg. Med. Chem.* **2012,** *20* (19), 5678-5698, https://doi.org/10.1016/j.bmc.2012.04.045.

(6) Obydennov, K. L.; Kalinina, T. A.; Galieva, N. A.; Beryozkina, T. V.; Zhang, Y.; Fan, Z. J.; Glukhareva, T. V.; Bakulev, V. A. Synthesis, Fungicidal Activity, and Molecular Docking of 2-Acylamino and 2-Thioacylamino Derivatives of 1*H*-benzo[*d*]imidazoles as Anti-Tubulin Agents. *J. Agric. Food Chem.* **2021,** *69* (40), 12048-12062, https://doi.org/10.1021/acs.jafc.1c03325.

(7) Danaher, M.; De Ruyck, H.; Crooks, S. R. H.; Dowling, G.; O'Keeffe, M. Review of methodology for the determination of benzimidazole residues in biological matrices. *J. Chromatogr. B* **2007,** *845* (1), 1-37, DOI: 10.1016/j.jchromb.2006.07.046.

(8) Gaba, M.; Singh, S.; Mohan, C. Benzimidazole: An emerging scaffold for analgesic and anti-inflammatory agents. *Eur. J. Med. Chem.* **2014,** *76*, 494-505, https://doi.org/10.1016/j.ejmech.2014.01.030.

(9) Veerasamy, R.; Roy, A.; Karunakaran, R.; Rajak, H. Structure-Activity Relationship Analysis of Benzimidazoles as Emerging Anti-Inflammatory Agents: An Overview. *Pharmaceuticals* **2021,** *14* (7), 663, https://doi.org/10.3390/ph14070663.

(10) Keri, R. S.; Rajappa, C. K.; Patil, S. A.; Nagaraja, B. M. Benzimidazole-core as an antimycobacterial agent. *Pharmacol. Rep.* **2016,** *68* (6), 1254-1265, DOI: 10.1016/j.pharep.2016.08.002.

(11) Tahlan, S.; Kumar, S.; Narasimhan, B. Antimicrobial potential of 1*H*-benzo[*d*]imidazole scaffold: a review. *BMC Chem.* **2019,** *13*, 18, https://doi.org/10.1186/s13065-019-0521-y.

(12) Silky, S.; Mandal, S. K.; Ewies, E. F.; Neerupma, D.; Arun, G. Synthesis, Characterization and Biological Evaluation of Benzimidazole And Benzindazole Derivatives as Anti-Hypertensive Agents. *Egypt. J. Chem.* **2021,** *64* (7), 3659-3664, DOI: 10.21608/EJCHEM.2021.79840.3931.

(13) Ueno, H.; Katoh, S.; Yokota, K.; Hoshi, J.; Hayashi, M.; Uchida, I.; Aisaka, K.; Hase, Y.; Cho, H. Structure-activity relationships of potent and selective factor Xa inhibitors: benzimidazole derivatives with the side chain oriented to the prime site of factor Xa. *Bioorg. Med. Chem. Lett.* **2004,** *14* (16), 4281-4286, DOI: 10.1016/j.bmcl.2004.05.092.

(14) Bharadwaj, S. S.; Poojary, B.; Nandish, S. K. M.; Kengaiah, J.; Kirana, M. P.; Shankar, M. K.; Das, A. J.; Kulal, A.; Sannaningaiah, D. Efficient Synthesis and in Silico Studies of the Benzimidazole Hybrid Scaffold with the Quinolinyloxadiazole Skeleton with Potential α-Glucosidase Inhibitory, Anticoagulant, and Antiplatelet Activities for Type-II Diabetes Mellitus Management and Treating Thrombotic Disorders. *ACS Omega* **2018,** *3* (10), 12562-12574, DOI: 10.1021/acsomega.8b01476.

(15) Al-Wasidi, A. S.; Refat, M. S.; Naglah, A. M.; Elhenawy, A. A. Different Potential Biological Activities of Benzimidazole Derivatives. *Egypt. J. Chem.* **2021,** *64* (5), 2631-2646, DOI: 10.21608/EJCHEM.2021.71477.3570.

(16) Choudhary, S.; Arora, M.; Verma, H.; Kumar, M.; Silakari, O. Benzimidazole based hybrids against complex diseases: A catalogue of the SAR profile. *Eur. J. Pharmacol.* **2021,** *899*, 174027, https://doi.org/10.1016/j.ejphar.2021.174027.

(17) Boiani, M.; González, M. Imidazole and benzimidazole derivatives as chemotherapeutic agents. *Mini-Rev. Med. Chem.* **2005,** *5* (4), 409-424, http://dx.doi.org/10.2174/1389557053544047.

(18) Bansal, Y.; Silakari, O. The therapeutic journey of benzimidazoles: A review. *Bioorg. Med. Chem.* **2012,** *20* (21), 6208-6236, https://doi.org/10.1016/j.bmc.2012.09.013.

(19) Shah, K.; Chhabra, S.; Shrivastava, S. K.; Mishra, P. Benzimidazole: a promising pharmacophore. *Med. Chem. Res.* **2013,** *22* (11), 5077-5104, https://doi.org/10.1007/s00044-013-0476-9.

(20) Keri, R. S.; Hiremathad, A.; Budagumpi, S.; Nagaraja, B. M. Comprehensive Review in Current Developments of Benzimidazole-Based Medicinal Chemistry. *Chem. Biol. Drug Des.* **2015,** *86* (1), 19-65, https://doi.org/10.1111/cbdd.12462.

(21) Yadav, G.; Ganguly, S. Structure activity relationship (SAR) study of benzimidazole scaffold for different biological activities: A mini-review. *Eur. J. Med. Chem.* **2015,** *97*, 419-443, http://dx.doi.org/10.1016/j.ejmech.2014.11.053.

(22) Gaba, M.; Mohan, C. Development of drugs based on imidazole and benzimidazole bioactive heterocycles: recent advances and future directions. *Med. Chem. Res.* **2016,** *25* (2), 173-210, https://doi.org/10.1007/s00044-015-1495-5.

(23) Akhtar, J.; Khan, A. A.; Ali, Z.; Haider, R.; Yar, M. S. Structure-activity relationship (SAR) study and design strategies of nitrogen-containing heterocyclic moieties for their anticancer activities. *Eur. J. Med. Chem.* **2017,** *125*, 143-189, http://dx.doi.org/10.1016/j.ejmech.2016.09.023.

(24) Akhtar, W.; Khan, M. F.; Verma, G.; Shaquiquzzaman, M.; Rizvi, M. A.; Mehdi, S. H.; Akhter, M.; Alam, M. M. Therapeutic evolution of benzimidazole derivatives in the last quinquennial period. *Eur. J. Med. Chem.* **2017,** *126*, 705-753, http://dx.doi.org/10.1016/j.ejmech.2016.12.010.

(25) Beltran-Hortelano, I.; Alcolea, V.; Font, M.; Pérez-Silanes, S. The role of imidazole and benzimidazole heterocycles in Chagas disease: A review. *Eur. J. Med. Chem.* **2020,** *206*, 112692, https://doi.org/10.1016/j.ejmech.2020.112692.

(26) Law, C. S. W.; Yeong, K. Y. Benzimidazoles in Drug Discovery: A Patent Review. *ChemMedChem* **2021,** *16* (12), 1861-1877, DOI: 10.1002/cmdc.202100004.

(27) Tonelli, M.; Simone, M.; Tasso, B.; Novelli, F.; Boido, V.; Sparatore, F.; Paglietti, G.; Pricl, S.; Giliberti, G.; Blois, S.; Ibba, C.; Sanna, G.; Loddo, R.; La Colla, P. Antiviral activity of benzimidazole derivatives. II. Antiviral activity of 2-phenylbenzimidazole derivatives. *Bioorg. Med. Chem.* **2010,** *18* (8), 2937-2953, DOI: 10.1016/j.bmc.2010.02.037.

(28) Kanwal, A.; Ahmad, M.; Aslam, S.; Naqvi, S. A. R.; Saif, M. J. Recent Advances in Antiviral Benzimidazole Derivatives: A Mini Review. *Pharm. Chem. J.* **2019,** *53* (3), 179-187, DOI: 10.1007/s11094-019-01976-3.

(29) Hue, B. T. B.; Nguyen, P. H.; De, T. Q.; Hieu, M. V.; Jo, E.; Tuan, N. V.; Thoa, T. T.; Anh, L. D.; Son, N. H.; Thanh, D. L. D.; Dupont-Rouzeyrol, M.; Grailhe, R.; Windisch, M. P. Benzimidazole Derivatives as Novel Zika Virus Inhibitors. *ChemMedChem* **2020,** *15* (15), 1453-1463, DOI: 10.1002/cmdc.202000124.

(30) Bessières, M.; Plebanek, E.; Chatterjee, P.; Shrivastava-Ranjan, P.; Flint, M.; Spiropoulou, C. F.; Warszycki, D.; Bojarski, A. J.; Roy, V.; Agrofoglio, L. A. Design, synthesis and biological evaluation of 2-substituted-6-[(4-substituted-1-piperidyl)methyl]-1H-benzimidazoles as inhibitors of Ebola virus infection. *Eur. J. Med. Chem.* **2021,** *214*, 113211, DOI: 10.1016/j.ejmech.2021.113211.

(31) Kumar, A.; De, S.; Moharana, A. K.; Nayak, T. K.; Saswat, T.; Datey, A.; Mamidi, P.; Mishra, P.; Subudhi, B. B.; Chattopadhyay, S. Inhibition of herpes simplex virus-1 infection by MBZM-N-IBT: in silico and in vitro studies. *Virol. J.* **2021,** *18* (1), 103, DOI: 10.1186/s12985-021-01581-5.

(32) Li, Y. F.; Wang, G. F.; He, P. L.; Huang, W. G.; Zhu, F. H.; Gao, H. Y.; Tang, W.; Luo, Y.; Feng, C. L.; Shi, L. P.; Ren, Y. D.; Lu, W.; Zuo, J. P. Synthesis and anti-hepatitis B virus activity of novel benzimidazole derivatives. *J. Med. Chem.* **2006,** *49* (15), 4790-4794, DOI: 10.1021/jm060330f.

(33) Ahmad, N.; Azad, M. I.; Khan, A. R.; Azad, I. Benzimidazole as a Promising Antiviral Heterocyclic Scaffold: A Review. *J. Sci. Arts* **2021,** (1), 273-284, https://doi.org/10.46939/J.Sci.Arts-21.1-b05.

(34) O'Sullivan, D. G.; Wallis, A. K. Vibrational Frequency Correlations in Heterocyclic Molecules. Part VIII. Infrared Spectra of Virus Inhibitors Related to Benzimidazole. *J. Chem. Soc.* **1965,** (APR), 2331-2337, DOI: 10.1039/JR9650002331.

(35) Plewe, M. B.; Gantla, V. R.; Sokolova, N. V.; Shin, Y. J.; Naik, S.; Brown, E. R.; Fetsko, A.; Zhang, L. H.; Kalveram, B.; Freiberg, A. N.; Henkel, G.; McCormack, K. Discovery of a novel highly potent broad-spectrum heterocyclic chemical series of arenavirus cell entry inhibitors. *Bioorg. Med. Chem. Lett.* **2021,** *41*, 127983, DOI: 10.1016/j.bmcl.2021.127983.

(36) Gholivand, K.; Mohammadpanah, F.; Pooyan, M.; Roohzadeh, R. Evaluating anti-coronavirus activity of some phosphoramides and their influencing inhibitory factors using molecular docking, DFT, QSAR, and NCI-RDG studies. *J. Mol. Struct.* **2022,** *1248*, 131481, DOI: 10.1016/j.molstruc.2021.131481.

(37) Syrbu, S. A.; Kiselev, A. N.; Lebedev, M. A.; Gubarev, Y. A.; Yurina, E. S.; Lebedeva, N. S. Synthesis of Hetaryl-Substituted Asymmetric Porphyrins and Their Affinity to SARS-CoV-2 Helicase. *Russ. J. Gen. Chem.* **2021,** *91* (6), 1039-1049, DOI: 10.1134/S1070363221060098.

(38) Rossi, F. V.; Gentili, D.; Marcantoni, E. Metal-Promoted Heterocyclization: A Heterosynthetic Approach to Face a Pandemic Crisis. *Molecules* **2021,** *26* (9), 2620, DOI: 10.3390/molecules26092620.

(39) López, D. A.; Simison, S. N.; de Sánchez, S. R. The influence of steel microstructure on CO_2 corrosion. EIS studies on the inhibition efficiency of benzimidazole. *Electrochim. Acta* **2003,** *48* (7), 845-854, https://doi.org/10.1016/S0013-4686(02)00776-4.

(40) Aljourani, J.; Raeissi, K.; Golozar, M. A. Benzimidazole and its derivatives as corrosion inhibitors for mild steel in 1M HCl solution. *Corrosion Sci.* **2009,** *51* (8), 1836-1843, https://doi.org/10.1016/j.corsci.2009.05.011.

(41) Obot, I. B.; Edouk, U. M. Benzimidazole: Small planar molecule with diverse anti-corrosion potentials. *J. Mol. Liq.* **2017,** *246*, 66-90, http://dx.doi.org/10.1016/j.molliq.2017.09.041.

(42) Marinescu, M. Recent advances in the use of benzimidazoles as corrosion inhibitors. *BMC Chem.* **2019,** *13* (1), 136, https://doi.org/10.1186/s13065-019-0655-y.

(43) Mishra, A.; Aslam, J.; Verma, C.; Quraishi, M. A.; Ebenso, E. E. Imidazoles as highly effective heterocyclic corrosion inhibitors for metals and alloys in aqueous electrolytes: A review. *J. Taiwan Inst. Chem. Eng.* **2020,** *114*, 341-358, https://doi.org/10.1016/j.jtice.2020.08.034.

(44) Goni, L. K. M. O.; Mazumder, M. A. J.; Quraishi, M. A.; Rahman, M. M. Bioinspired Heterocyclic Compounds as Corrosion Inhibitors: A Comprehensive Review. *Chem.-Asian J.* **2021,** *16* (11), 1324-1364, https://doi.org/10.1002/asia.202100201.

(45) Horak, E.; Kassal, P.; Murković-Steinberg, I. Benzimidazole as a structural unit in fluorescent chemical sensors: the hidden properties of a multifunctional heterocyclic scaffold. *Supramol. Chem.* **2018,** *30* (10), 838-857, https://doi.org/10.1080/10610278.2017.1403607.

(46) Wright, J. B. The Chemistry of the Benzimidazoles. *Chem. Rev.* **1951,** *48* (3), 397-541, https://doi.org/10.1021/cr60151a002.

(47) Preston, P. N. Synthesis, Reactions, and Spectroscopic Properties of Benzimidazoles. *Chem. Rev.* **1974,** *74* (3), 279-314, https://doi.org/10.1021/cr60289a001.

(48) Singh, V. K.; Parle, A. The Intriguing Benzimidazole: A Review. *Int. J. Pharm. Sci. Res.* **2019,** *10* (4), 1540-1552, DOI: 10.13040/IJPSR.0975-8232.10(4).1540-52.

(49) Pardeshi, V. A. S.; Chundawat, N. S.; Pathan, S. I.; Sukhwal, P.; Chundawat, T. P. S.; Singh, G. P. A review on synthetic approaches of benzimidazoles. *Synth. Commun.* **2021,** *51* (4), 485-513, https://doi.org/10.1080/00397911.2020.1841239.

(50) Skolia, E.; Apostolopoulou, M. K.; Nikitas, N. F.; Kokotos, C. G. Photochemical Synthesis of Benzimidazoles from Diamines and Aldehydes. *Eur. J. Org. Chem.* **2021,** *2021* (3), 422-428, DOI: 10.1002/ejoc.202001357.

(51) Qi, Z. L.; Yang, Y.; Miao, T.; Li, L. F.; Fu, X. L. Progress in Photocatalytic Synthesis of Benzimidazoles. *ChemistrySelect* **2021,** *6* (45), 12628-12643, DOI: 10.1002/slct.202103475.

(52) Vijayan, N.; Babu, R. R.; Gopalakrishnan, R.; Ramasamy, P.; Harrison, W. T. A. Growth and characterization of benzimidazole single crystals: a nonlinear optical material. *J. Cryst. Growth* **2004,** *262* (1-4), 490-498, https://doi.org/10.1016/j.jcrysgro.2003.08.082.

(53) Vijayan, N.; Balamurugan, N.; Babu, R. R.; Gopalakrishnan, R.; Ramasamy, P.; Harrison, W. T. A. Bulk growth of benzimidazole single crystals by vertical Bridgman technique (VBT). *J. Cryst. Growth* **2004,** *267* (1-2), 218-222, https://doi.org/10.1016/j.jcrysgro.2004.03.064.

(54) Vijayan, N.; Bhagavannarayana, G.; Babu, R. R.; Gopalakrishnan, R.; Maurya, K. K.; Ramasamy, P. A comparative study on solution-and Bridgman-grown single crystals of benzimidazole by high-resolution X-ray diffractometry, Fourier transform infrared, microhardness, laser damage threshold, and second-harmonic generation measurements. *Cryst. Growth Des.* **2006,** *6* (6), 1542-1546, https://doi.org/10.1021/cg060002g.

(55) Vijayan, N.; Bhagavannarayana, G.; Balamurugan, N.; Babu, R. R.; Maurya, K. K.; Gopalakrishnan, R.; Ramasamy, P. Studies on the growth and characterization of benzimidazole single crystals - vertical Bridgman technique. *J. Cryst. Growth* **2006,** *293* (2), 318-323, DOI: 10.1016/j.jcrysgro.2006.04.115.

(56) Vijayan, N.; Nagarajan, K.; Slawin, A. M. Z.; Nair, C. K. S.; Bhagavannarayana, G. Growth of benzimidazole single crystal by Sankaranarayanan-Ramasamy method and its characterization by high-resolution X-ray diffraction, thermogravimetric/differential thermal analysis, and birefringence studies. *Cryst. Growth Des.* **2007,** *7* (2), 445-448, https://doi.org/10.1021/cg0605180.

(57) Kanagasekaran, T.; Mythili, P.; Srinivasan, P.; Vijayan, N.; Bhagavannarayana, G.; Kulriya, P. K.; Kanjilal, D.; Gopalakrishnan, R.; Ramasamy, P. Effects of 50 MeV Si ion irradiation on nonlinear optical benzimidazole single crystals. *Cryst. Res. Technol.* **2007,** *42* (12), 1376-1381, https://doi.org/10.1002/crat.200711035.

(58) Vijayan, N.; Bhagavannarayana, G.; Budakoti, G. C.; Kumar, B.; Upadhyaya, V.; Das, S. Optical, dielectric and surface studies on solution grown benzimidazole single crystals. *Mater. Lett.* **2008,** *62* (8-9), 1252-1254, https://doi.org/10.1016/j.matlet.2007.08.023.

(59) Vijayan, N.; Bhagavannarayana, G.; Halder, S. K.; Verma, S.; Philip, J.; Philip, R.; Rathi, B. X-ray topography, photopyroelectric and two-photon absorption studies on solution grown benzimidazole single crystal. *Appl. Phys. A-Mater. Sci. Process.* **2013,** *110* (1), 55-58, https://doi.org/10.1007/s00339-012-7448-7.

(60) Derkosch, J.; Polansky, O. E.; Rieger, E.; Derflinger, G. Über Benzazole. 2. Die UV-Spektren der Benzazole. *Mon. Chem.* **1961,** *92* (6), 1131-1141, https://doi.org/10.1007/BF00914977.

(61) Schütt, H. U.; Zimmermann, H. Polarisation der Elektronenbanden von Aromaten. 7. Mitteilung: Indol, Indazol, Benzimidazol, Benztriazol, Carbazol. *Ber. Bunsen Ges. Phys. Chem.* **1963,** *67* (1), 54-62, https://doi.org/10.1002/bbpc.19630670110.

(62) Morgan, K. J. Infrared Spectra of Some Simple Benzimidazoles. *J. Chem. Soc.* **1961,** (JUN), 2343-2347, https://doi.org/10.1039/JR9610002343.

(63) Cordes, M. M.; Walter, J. L. Infrared and Raman Studies of Heterocyclic Compounds. II. Infrared Spectra and Normal Vibrations of Benzimidazole and Bis-(Benzimidazolato)-Metal Complexes. *Spectroc. Acta Pt. A-Molec. Spectr.* **1968,** *24* (9), 1421-1435, DOI: 10.1016/0584-8539(68)80165-5.

(64) Escande, A.; Galigné, J. L. Structure cristalline du benzimidazole, $C_7N_2H_6$: comparaison des résultats de édeux tudes indépendantes. *Acta Crystallogr. Sect. B-Struct. Commun.* **1974,** *B30* (JUN15), 1647-1648, https://doi.org/10.1107/S0567740874005528.

(65) Krawczyk, S.; Gdaniec, M. Polymorph β of 1*H*-benzimidazole. *Acta Crystallogr. Sect. E.-Crystallogr. Commun.* **2005,** *61*, o4116-o4118, https://doi.org/10.1107/S1600536805036536.

(66) Zielinski, W.; Katrusiak, A. Hydrogen Bonds NH ... N in Compressed Benzimidazole Polymorphs. *Cryst. Growth Des.* **2013,** *13* (2), 696-700, https://doi.org/10.1021/cg301374z.

(67) Muthuraja, A.; Kalainathan, S. Growth of organic benzimidazole (BMZ) single crystal by vertical Bridgman technique and its structural, spectral, thermal, optical, mechanical and dielectric properties. *Opt. Mater.* **2015,** *47*, 354-360, https://doi.org/10.1016/j.optmat.2015.06.001.

(68) Mohan, S.; Sundaraganesan, N.; Mink, J. FTIR and Raman Studies on Benzimidazole. *Spectroc. Acta Pt. A-Molec. Biomol. Spectr.* **1991,** *47* (8), 1111-1115, https://doi.org/10.1016/0584-8539(91)80042-H.

(69) Morsy, M. A.; Al-Khaldi, M. A.; Suwaiyan, A. Normal vibrational mode analysis and assignment of benzimidazole by ab initio and density functional calculations and polarized infrared and Raman spectroscopy. *J. Phys. Chem. A* **2002,** *106* (40), 9196-9203, https://doi.org/10.1021/jp0256948.

(70) Sundaraganesan, N.; Ilakiamani, S.; Subramani, P.; Joshua, B. D. Comparison of experimental and ab initio HF and DFT vibrational spectra of benzimidazole. *Spectroc. Acta Pt. A-Molec. Biomolec. Spectr.* **2007,** *67* (3-4), 628-635, https://doi.org/10.1016/j.saa.2006.08.020.

(71) Tomkinson, J. Reassignment of the vibrational spectrum of benzimidazole. *J. Phys. Chem. A* **2008,** *112* (27), 6115-6119, https://doi.org/10.1021/jp8008783.

(72) Nieto, C. I.; Cabildo, P.; Garcia, M. A.; Claramunt, R. M.; Alkorta, I.; Elguero, J. An experimental and theoretical NMR study of NH-benzimidazoles in solution and in the solid state: proton transfer and tautomerism. *Beilstein J. Org. Chem.* **2014,** *10*, 1620-1629, https://doi.org/10.3762/bjoc.10.168.

(73) Larina, L. I., Tautomerism and Structure of Azoles: Nuclear Magnetic Resonance Spectroscopy. In *Advances in Heterocyclic Chemistry*, Scriven, E. F. V.; Ramsden, C. A., Eds. Elsevier Academic Press Inc: San Diego, 2018; Vol. 124, pp 233-321, http://dx.doi.org/10.1016/bs.aihch.2017.06.003.

(74) Klots, T. D.; Devlin, P.; Collier, W. B. Heteroatom derivatives of indene V. Vibrational spectra of benzimidazole. *Spectroc. Acta Pt. A-Molec. Biomolec. Spectr.* **1997,** *53* (14), 2445-2456, https://doi.org/10.1016/S1386-1425(97)00154-6.

(75) Schoone, K.; Smets, J.; Houben, L.; Van Bael, M. K.; Adamowicz, L.; Maes, G. Matrix-isolation FT-IR studies and theoretical calculations of hydrogen-bonded complexes of molecules modeling adenine tautomers. 1. H-bonding of benzimidazoles with H_2O in Ar matrices. *J. Phys. Chem. A* **1998,** *102* (25), 4863-4877, https://doi.org/10.1021/jp980373a.

(76) Cané, E.; Trombetti, A.; Velino, B.; Caminati, W. Assignment of the 278-nm Electronic Band System of Benzimidazole [1,3-Benzodiazole] as $\pi^*-\pi$ by Rotational Band Contour Analysis. *J. Mol. Spectrosc.* **1991,** *150* (1), 222-228, https://doi.org/10.1016/0022-2852(91)90205-O.

(77) Velino, B.; Trombetti, A.; Canè, E.; Corbelli, G.; Caminati, W. Microwave Spectrum of Benzimidazole. *J. Mol. Spectrosc.* **1992,** *152* (2), 434-440, https://doi.org/10.1016/0022-2852(92)90080-8.

(78) Gordon, R. D.; Yang, R. F. Vapor Absorption Spectra of Benzoxazole, Benzimidazole, and Benzothiazole near 2850 Å. *Can. J. Chem.* **1970,** *48* (11), 1722-1729, https://doi.org/10.1139/v70-283.

(79) Gordon, R. D.; Chan, W. H. W. Origin Band Shifts Upon Deuteration in the 280 nm Absorption System of Benzimidazole. *Spectr. Lett.* **1977,** *10* (7), 571-586, https://doi.org/10.1080/00387017708064989.

(80) Jalviste, E.; Treshchalov, A. Spectroscopy of Jet-Cooled Benzimidazole and Benzotriazole. *Chem. Phys.* **1993,** *172* (2-3), 325-338, https://doi.org/10.1016/0301-0104(93)80127-U.

(81) Berden, G.; Meerts, W. L.; Jalviste, E. Rotationally Resolved Ultraviolet Spectroscopy of Indole, Indazole, and Benzimidazole: Inertial Axis Reorientation in the $S_1(^1L_b)$<-S_0 Transitions. *J. Chem. Phys.* **1995,** *103* (22), 9596-9606, https://doi.org/10.1063/1.469974.

(82) Schmitt, M.; Krügler, D.; Böhm, M.; Ratzer, C.; Bednarska, V.; Kalkman, I.; Meerts, W. L. A genetic algorithm based determination of the ground and excited (^1L_b) state structure and the orientation of the transition dipole moment of benzimidazole. *Phys. Chem. Chem. Phys.* **2006,** *8* (2), 228-235, https://doi.org/10.1039/B512686J.

(83) Lin, J. L.; Li, Y. C.; Tzeng, W. B. Mass analyzed threshold ionization spectroscopy of aza-aromatic bicyclic molecules: Benzimidazole and benzotriazole. *Chem. Phys.* **2007,** *334* (1-3), 189-195, https://doi.org/10.1016/j.chemphys.2007.03.002.

(84) Brand, C.; Rolf, J.; Wilke, M.; Schmitt, M. High Resolution Electronic Spectroscopy of Vibrationally Hot Bands of Benzimidazole. *J. Phys. Chem. A* **2013,** *117* (48), 12812-12820, https://doi.org/10.1021/jp408755q.

(85) Stuhlmann, B.; Gmerek, F.; Krügler, D.; Schmitt, M. Determination of the geometry change of benzimidazole upon electronic excitation from a combined Franck-Condon/rotational constants fit. *J. Mol. Struct.* **2014,** *1072*, 45-52, https://doi.org/10.1016/j.molstruc.2014.04.005.

(86) Serrano-Andrés, L.; Borin, A. C. A theoretical study of the emission spectra of indole and its analogs: indene, benzimidazole, and 7-azaindole. *Chem. Phys.* **2000,** *262* (2-3), 267-283, https://doi.org/10.1016/S0301-0104(00)00335-9.

(87) Borin, A. C.; Serrano-Andrés, L. A theoretical study of the absorption spectra of indole and its analogs: indene, benzimidazole, and 7-azaindole. *Chem. Phys.* **2000,** *262* (2-3), 253-265, https://doi.org/10.1016/S0301-0104(00)00336-0.

(88) Arulmozhiraja, S.; Coote, M. L. ^1L_a and ^1L_b States of Indole and Azaindole: Is Density Functional Theory Inadequate? *J. Chem. Theory Comput.* **2012,** *8* (2), 575-584, https://doi.org/10.1021/ct200768b.

(89) Yang, P.; Pang, M.; Li, M.; Shen, W.; He, R. X. Vibrationally resolved ^1L_b ($^1A'$) $\leftrightarrow$ S_0 ($^1A'$) electronic spectra of benzimidazole and indene: Influence of Duschinsky and Herzberg-Teller effects on weak dipole-allowed transitions. *Spectroc. Acta Pt. A-Molec. Biomolec. Spectr.* **2015,** *151*, 375-384, https://doi.org/10.1016/j.saa.2015.06.101.
